# Supplementary material for: A Palladium-Catalyzed 4CzIPN-Mediated Decarboxylative Acylation Reaction of O-Methyl Ketoximes with α-Keto Acids under Visible Light
Source: Molecules. 2022 Mar 18;27(6):1980. doi: 10.3390/molecules27061980 (PMC8955727; doi:10.3390/molecules27061980)

Supporting Information

A palladium-catalyzed 4CzIPN mediated decarboxylative acylation reaction of O-methyl ketoximes with α-keto acids under visible light

Cheng Wang [1], Shan Yang [1], Zhibin Huang [1,*] and Yingsheng Zhao [1,2,*]

[1] Key Laboratory of Organic Synthesis of Jiangsu Province, College of Chemistry, Chemical Engineering and Materials Science.

Soochow University, Suzhou 215123 (PR China)

E-mail: zbhuang@suda.edu.cn

E-mail: yszhao@suda.edu.cn

[2] School of Chemistry and Chemical Engineering,

Henan Normal University, Xinxiang 453000, P.R.China

Table of Contents

1. **General Information** **S2**
2. **Procedure for Preparation of 2b-2l** **S2**
3. **Procedure for Preparation of 1a-1w** **S2**
4. **General Procedures for Ortho-Benzoylation of Acetophenone Oxime Ether** **S3**
5. **Gram-Scale Reaction** **S3**
6. **Further Transformation** **S3**
7. **Mechanism Exploration Experiment** **S5**
8. **Spectroscopic Data of Compounds** **S7**
9. **NMR spctra** **S21**

1. General Information

Unless otherwise noted, all reagents were purchased from Acros, Alfa and Adamas and used without further purification. Column chromatography purifications were performed using 200–300 mesh silica gel. NMR spectra were recorded on Varian Inova-400 MHz, Inova-300 MHz, Bruker DRX-400 or Bruker DRX-500 instruments and cal-ibrated using residual solvent peaks as internal references. All heating reactions were conducted in an oil bath. Multiplicities are recorded as follows: s = singlet, d = doublet, t = triplet, dd = doublet of doublets, m = multiplet. HRMS analyses were carried out using a TOF-MS instrument with an EI source. ESR: JES-X320 electron spin resonance spec-trometer.

2. Procedure for Preparation of 2b–2l

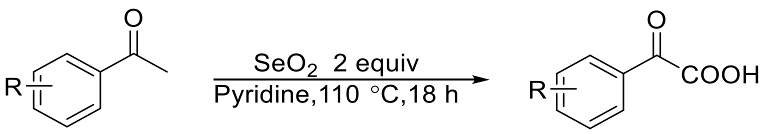

A flame-dried pressure tube was charged with the specified acetophenone (4.0 mmol, 1.0 equiv.) and selenium dioxide (0.89 g, 8.0 mmol, 2.0 equiv.). Dry pyridine (5 mL) was added, the tube was sealed, and the reaction mixture was heated at 110 °C for 18 h. Afterwards, the reaction was cooled to room temperature and filtered. The filtrate was acidified with 1 M HCl solution and extracted three times with EtOAc. Subsequently, the combined organic layers were extracted three times with 1 M NaOH solution. The combined aqueous layers were acidified with conc. HCl and extracted three times with EtOAc. The combined organic layers were dried over Na_2SO_4 and concentrated in vacuo. Purification by column chromatography yielded the products **2b–2l**. The characteriza-tion results are consistent with the references.

3. Preparation for Preparation of 1a–1w

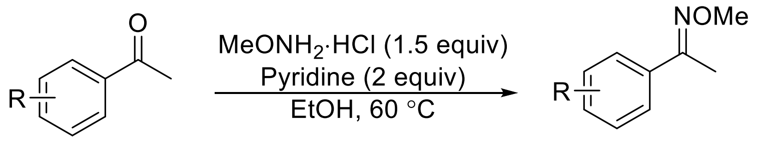

To a solution of ketones (22.0 mmol) and pyridine (5.0 mL, 61.8 mmol) in EtOH (10 mL), NH_2OMe•HCl (2.29 g, 33.0 mmol) was added in one portion, and the reaction

mixture was stirred at 60 °C for 6 h. The reaction was quenched by adding water and extracted twice with ethyl acetate. The combined extracts were washed with aqueous HCl and brine and dried over $MgSO_4$. The solvents were removed under reduced pressure. Further recrystallization was conducted from ethyl acetate-hexane to provide O-methyl ketoximes.

4. General Procedures for Ortho-Benzoylation of Acetophenone Oxime Ether

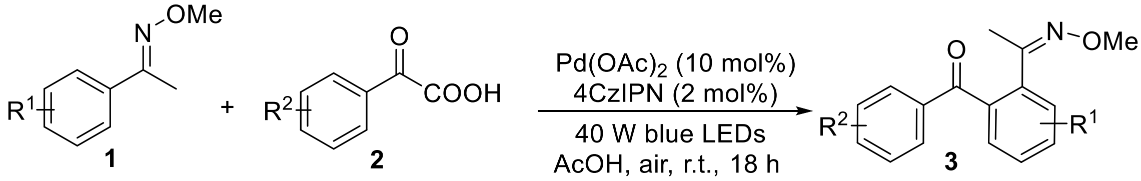

Ketoxime ether (**1**, 0.10 mmol), aroylformic acid (**2**, 0.30 mmol), $Pd(OAc)_2$ (10 mol%), 4CzIPN (2 mol%) and AcOH (0.2 M) were added to an oven-dried reaction vessel equipped with a magnetic stirring bar, and the reaction vessel was irradiated using 40W blue LEDs at r.t. for 18 h. After the reaction was completed, the reaction solution was concentrated under reduced pressure to yield crude product **3**, which was purified by flash chromatography (silica gel, petroleum ether/ethyl acetate).

5. Gram-Scale Reaction

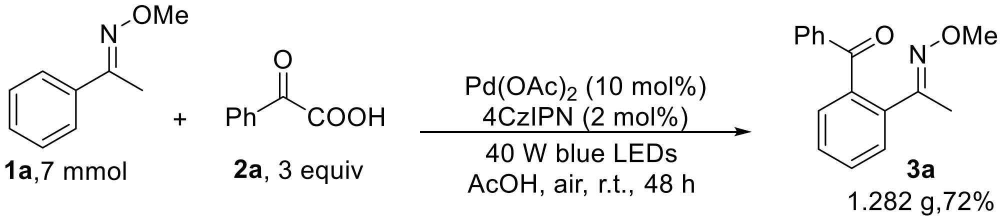

Acetophenone oxime ether (**1a**, 7 mmol), benzoylformic acid (**2a**, 21 mmol), $Pd(OAc)_2$ (10 mol%), 4CzIPN (2 mol%) and AcOH (0.2 M) were added to an oven-dried reaction vessel equipped with a magnetic stirring bar, and the reaction vessel was irra-diated using 40W blue LEDs at r.t. for 48 h. After the reaction was completed, the reaction solution was concentrated under reduced pressure to yield crude product **3a**, which was purified by flash chromatography (silica gel, petroleum ether/ethyl acetate = 50:1).

6. Further Transformation

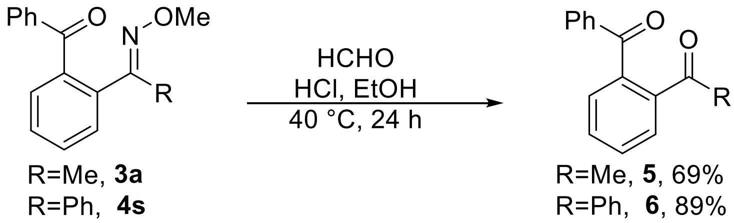

A 15-milliliter vial equipped with a magnetic stirrer was charged with **3a** (or **4s**) (0.2 mmol) and EtOH (1 mL). Then, 37 wt.% formaldehyde solution (1 mL) and concentrated hydrochloric acid (160 μL) were added, and the mixture was stirred and heated at 40 °C for 24 h. The reaction was diluted with ethyl acetate. The mixture was concentrated under reduced pressure and then purified by flash column chromatography to give product **5** (or **6**).

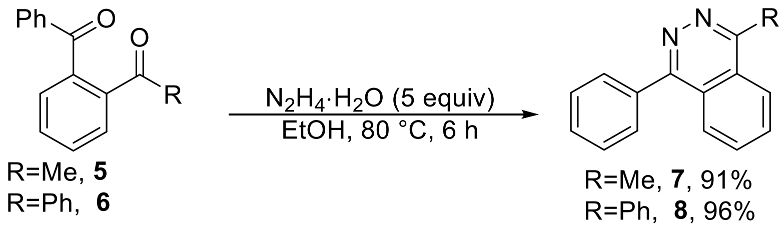

To the solution of the 1,2-diacylbenzenes (**5** or **6**) (0.2 mmol) in ethanol (2 mL) was added hydrazine hydrate (1 mmol), and the resulting mixture was heated at reflux for 6 h under nitrogen. After cooling to room temperature, ethanol was removed under vacuum to give the crude product, which was purified by silica gel column chromatography (ethyl acetate, hexane and methanol) to give the phthalazines (**7** or **8**).

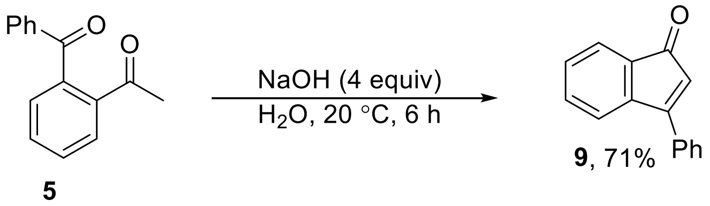

To the solution of the 1-(2-benzoylphenyl)ethan-1-one **5** (0.2 mmol) in H_2O (1 mL) was added sodium hydroxide (1 mmol), and the resulting mixture was heated at 20 °C for 6 h. The reaction was diluted with ethyl acetate. The mixture was concentrated under reduced pressure and then purified by flash column chromatography to give product **9**.

7. Mechanism Exploration Experiment

7.1 Radical Capture Experiment

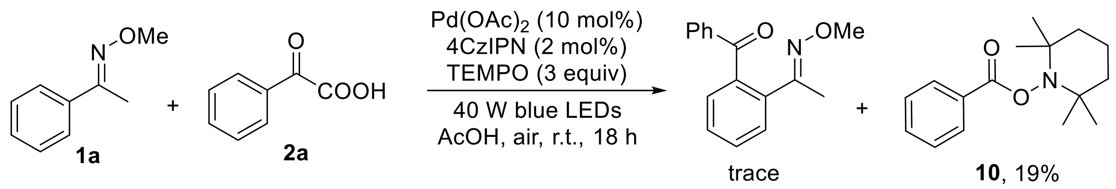

Acetophenone oxime ether (**1a**, 0.1 mmol), benzoylformic acid (**2a**, 0.3 mmol), $Pd(OAc)_2$ (10 mol%), 4CzIPN (2 mol%), 2,2,6,6-tetramethyl-1-piperinedinyloxy(TEMPO) (0.3 mmol) and AcOH (0.2 M) were added to an oven-dried reaction vessel equipped with a magnetic stirring bar, and the reaction vessel was irradiated using 40W blue LEDs at r.t. for 18 h. After the reaction was completed, the reaction solution was concentrated under reduced pressure to yield crude product **10**, which was purified by flash chro-matography (silica gel, petroleum ether/ethyl acetate = 30:1).

7.2 Experimental Procedure of Kinetic Isotope Effect

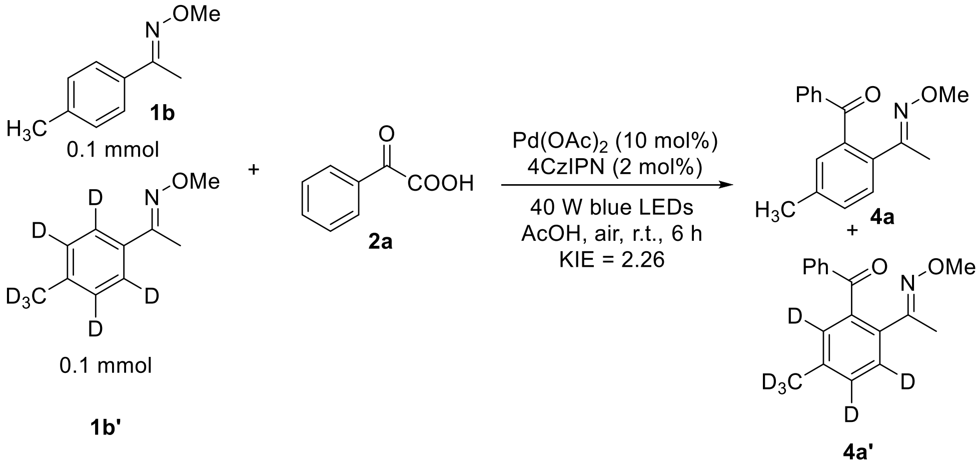

$Pd(OAc)_2$ (0.01 mmol, 2.3 mg), 4CzIPN (0.002 mmol, 1.6 mg), **1b** (0.1 mmol,) **1b’** (0.1 mmol), benzoylformic acid (0.3 mmol) and acetic acid (0.5 mL) were added in sequence to a 5-milliliter glass reaction flask with a magnetic stir bar, and the reaction flask was irradiated under 40W blue LEDs and stirred at r.t. for 6 h. After the reaction, the reaction solution was diluted with ethyl acetate and filtered through Celite. The filtrate was concentrated in vacuo and purified by silica gel column chromatography to obtain the target products **4a** and **4a’**. According to the 1H NMR results, we calculated KH:KD = 2.26 (2.08÷(3−2.08) = 2.26).

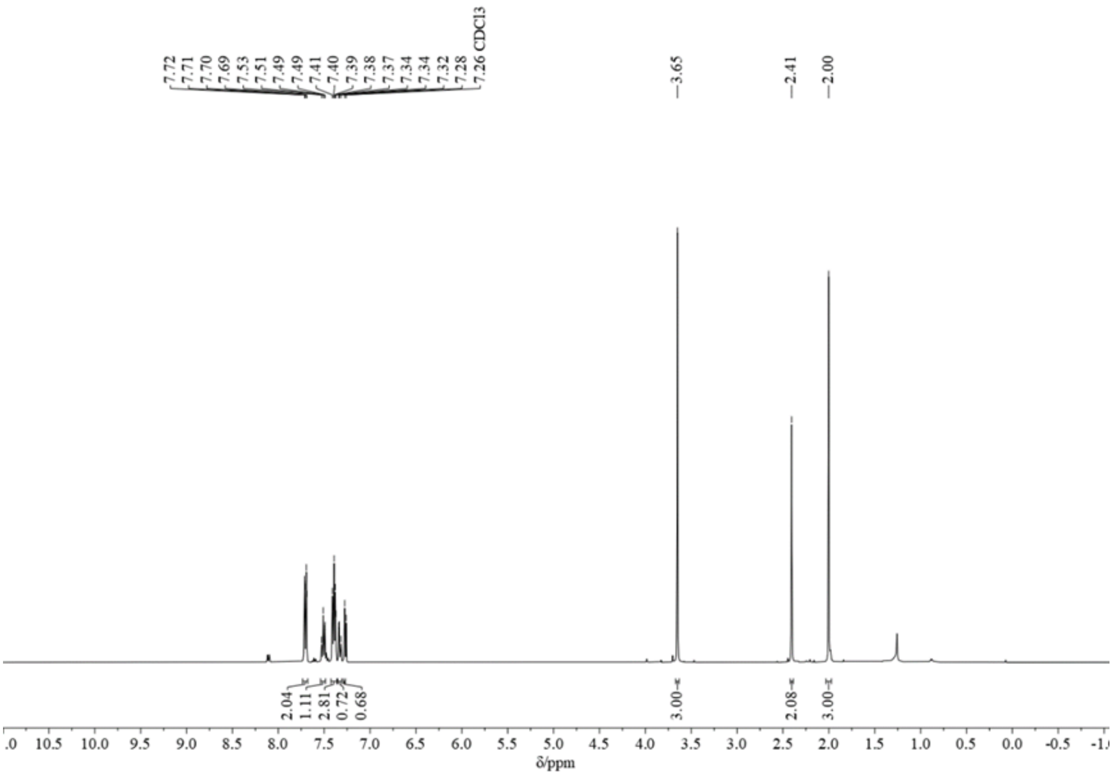

7.3 Determination of Superoxide Radical Anion

A superoxide radical anion ($O_2^{\bullet -}$) was generated from molecular oxygen by single electron transfer (SET). We used 5,5-dimethyl-1-pyrroline-N-oxide (DMPO) as a probe to capture the active species $O_2^{\bullet -}$. As shown in Figure 2, when the solution of DMPO, 2a and 4CzIPN was in chlorobenzene solution without irradiation, no signal was detected. In contrast, when the same solution was irradiated with blue LEDs, the signal showing that $O_2^{\bullet -}$ was trapped by DMPO was observed.

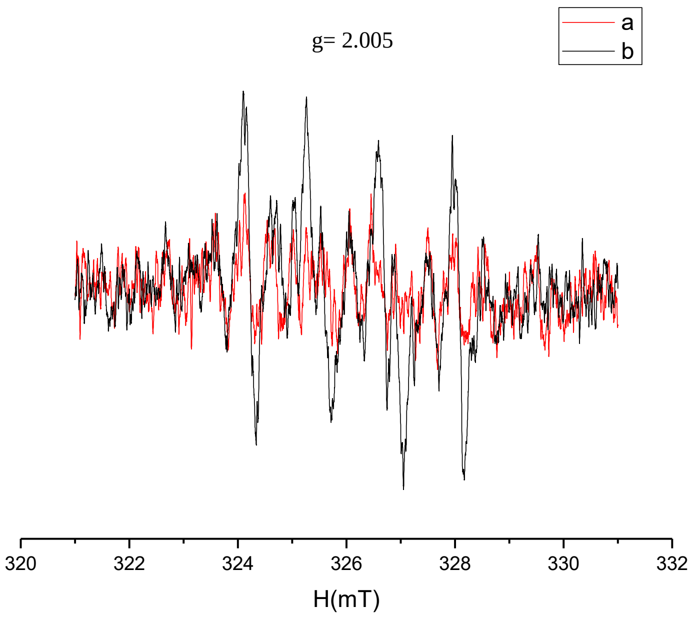

Figure 2. ESR spectra of air-saturated chlorobenzene solution of **2a** (1.0×10^{-3} M), 4CzIPN (2.0×10^{-5} M) and DMPO (2.0×10^{-2} M). (a) Without blue LEDs irradiation; (b) under blue LEDs irradiation for 60 s. ESR setting parameters (cf: 326.000; st: 30; rmfirst fq: 100.00; md: 0.3 × 0.1; am: 8.00 × 100; tc: 0.03; uF: 9150.577000 MHz; uP: 0.99800 mW; accumu = 3).

8. Spectroscopic Data of Compounds

(*E*)-(2-(1-(methoxyimino)ethyl)phenyl)(phenyl)methanone **(3a)**

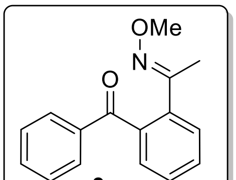

3a

white solid, m.p. 93-95 ºC. 21.5 mg, 85%. ^{1}H NMR (400 MHz, $CDCl_3$) δ 7.72-7.69 (m, 2H, ArH), 7.54-7.45 (m, 5H, ArH), 7.42-7.38 (m, 2H, ArH), 3.67 (s, 3H, CH_3,), 2.03 (s, 3H, CH_3). ^{13}C NMR (100 MHz, $CDCl_3$) δ 197.7, 154.1, 138.9, 138.3, 136.5, 132.7, 130.3, 129.4, 129.1, 128.7, 128.3, 127.8, 61.8, 14.5. HRMS Calcd for $C_{16}H_{16}NNaO_2$ $[M+H]^+$: 254.1176; Found: 254.1168.

(*E*)-(4-fluorophenyl)(2-(1-(methoxyimino)ethyl)phenyl)methanone **(3b)**

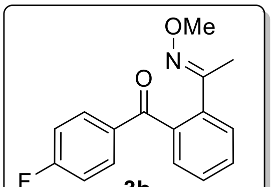

3b

Colorless liquid. 16.5 mg, 61%. ^{1}H NMR (400 MHz, $CDCl_3$) δ 7.75-7.72 (m, 2H, ArH), 7.56-7.48, (m, 2H, ArH), 7.47-7.42 (m, 2H, ArH), 7.09-7.05 (m, 2H, ArH), 3.67 (s, 3H, CH_3), 2.05 (s, 3H, CH_3). ^{19}F NMR (377 MHz, $CDCl_3$) δ -106.02. ^{13}C NMR

(100 MHz, $CDCl_3$) δ 196.2, 166.8, 164.2, 153.9, 137.5 (d, $J_{C\text{-}F}$= 242 Hz), 134.7 (d, $J_{C\text{-}F}$= 3 Hz), 133.0 (d, $J_{C\text{-}F}$= 9 Hz), 132.0, 131.9, 130.4, 128.9, 128.8, 127.8, 115.5 (d, $J_{C\text{-}F}$= 22 Hz), 61.8, 14.4. HRMS Calcd for $C_{16}H_{14}FNNaO_2$ $[M+Na]^+$: 294.0901; Found: 294.0890.

(*E*)-(4-chlorophenyl)(2-(1-(methoxyimino)ethyl)phenyl)methanone **(3c)**

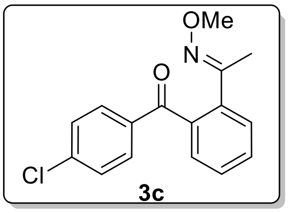

Colorless liquid. 16.1 mg, 56%. ^{1}H NMR (400 MHz, $CDCl_3$) δ 7.65-7.63 (m, 2H, ArH), 7.56-7.42 (m, 4H ArH), 7.38-7.36 (m, 2H, ArH), 3.67 (s, 3H, CH_3), 2.05 (s, 3H, CH_3). ^{13}C NMR (100 MHz, $CDCl_3$) δ 196.5, 153.8, 139.0, 138.6, 136.8, 136.3, 130.7, 130.5, 129.0, 128.8, 128.7, 127.8, 61.9, 14.3. HRMS Calcd for $C_{16}H_{14}ClNNaO_2$ $[M+Na]^+$: 310.0605; Found: 310.0619.

(*E*)-(4-bromophenyl)(2-(1-(methoxyimino)ethyl)phenyl)methanone **(3d)**

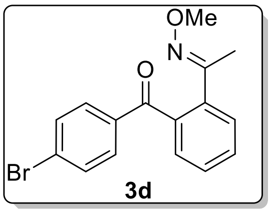

Colorless liquid. 25.2 mg, 76%. ^{1}H NMR (400 MHz, $CDCl_3$) δ 7.58- 7.51 (m, 6H, ArH), 7.49-7.42 (m, 2H, ArH,), 3.66 (s, 3H, CH_3), 2.05 (s, 3H, CH_3). ^{13}C NMR (100 MHz, $CDCl_3$) δ 196.6, 153.7, 138.5, 137.2, 136.2, 131.7, 130.8, 130.5, 128.9, 128.8, 127.7, 127.6, 61.8, 14.3. HRMS Calcd for $C_{16}H_{14}BrNNaO_2$ $[M+Na]^+$: 354.0100; Found: 354.0104.

(*E*)-(2-(1-(methoxyimino)ethyl)phenyl)(4-methoxyphenyl)methanone **(3e)**

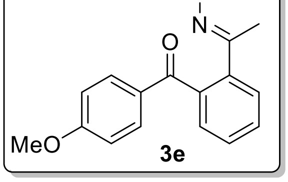

Colorless liquid. 13 mg, 46%. ^{1}H NMR (400 MHz, $CDCl_3$) δ 7.71 (d, J = 8 Hz, 2H, ArH), 7.50-7.49 (m, 2H, ArH), 7.44-7.43 (m, 2H, ArH), 6.89 (d, J = 8 Hz, 2H, ArH), 3.86 (s, 3H, CH_3), 3.70 (s, 3H, CH_3), 2.04 (s, 3H CH_3). ^{13}C NMR (100 MHz, $CDCl_3$) δ 196.5, 163.3, 154.4, 139.3, 136.5, 131.9, 131.1, 130.0, 128.9, 128.6, 127.9, 113.6, 61.8, 55.6, 14.8. HRMS Calcd for $C_{17}H_{18}NO_3$ $[M+H]^+$: 284.1281; Found: 284.1287.

(E)-(2-(1-(methoxyimino)ethyl)phenyl)(3-methoxyphenyl)methanone **(3f)**

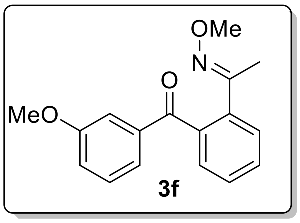

Colorless liquid. 11.9 mg, 42%. ^{1}H NMR (400 MHz, $CDCl_3$) δ 7.53-7.45 (m, 4H, ArH), 7.34-7.33 (m, 1H, ArH), 7.29-7.25 (m, 1H, ArH), 7.17 (d, J = 8 Hz, 1H ArH,), 7.07-7.05 (m, 1H, ArH), 3.82 (s, 3H, CH_3), 3.70 (s, 3H, CH_3), 2.03 (s, 3H, CH_3). ^{13}C NMR (100 MHz, $CDCl_3$) δ 197.4, 159.7, 154.2, 139.7, 138.9, 136.6, 130.3, 129.3, 129.1, 128.7, 127.8, 122.4, 119.2, 113.4, 61.8, 55.5, 14.6. HRMS Calcd for $C_{17}H_{18}NO_3$ $[M+H]^+$: 284.1281; Found: 284.1289.

(E)-(3-fluorophenyl)(2-(1-(methoxyimino)ethyl)phenyl)methanone **(3g)**

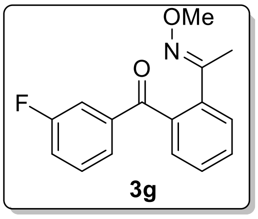

Colorless liquid. 17.0 mg, 63%. ^{1}H NMR (400 MHz, $CDCl_3$) δ 7.57-7.33 (m, 7H, ArH), 7.24-7.19 (m, 1H ArH), 3.67 (s, 3H, CH_3), 2.05 (s, 3H, CH_3). ^{19}F NMR (377 MHz, $CDCl_3$) δ -112.40. ^{13}C NMR (100 MHz, $CDCl_3$) δ 196.3, 196.3, 164.0, 161.6, 153.8, 140.7 (d, $J_{C\text{-}F}$= 6 Hz), 138.4, 136.3, 130.6, 130.0 (d, $J_{C\text{-}F}$= 8 Hz), 129.1, 128.9, 127.7 (d, $J_{C\text{-}F}$= 267 Hz), 125.1 (d, $J_{C\text{-}F}$= 3 Hz), 119.6 (d, $J_{C\text{-}F}$= 22 Hz), 115.8 (d, $J_{C\text{-}F}$= 22 Hz), 61.9, 14.2. HRMS Calcd for $C_{16}H_{14}FNNaO_2$ $[M+Na]^+$: 294.0901; Found: 294.0910.

(E)-(3-chlorophenyl)(2-(1-(methoxyimino)ethyl)phenyl)methanone **(3h)**

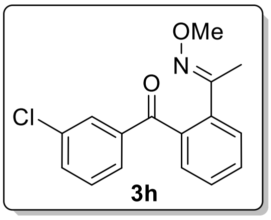

Colorless liquid. 13.2 mg, 46%. ^{1}H NMR (400 MHz, $CDCl_3$) δ 7.67-7.66 (m, 1H, ArH), 7.57-7.54 (m, 2H, ArH), 7.52-7.31 (m, 4H, ArH), 7.35-7.31 (m, 1H, ArH), 3.68 (s, 3H, CH_3), 2.04 (s, 3H CH_3). ^{13}C NMR (100 MHz, $CDCl_3$) δ 196.3, 153.8, 140.1, 138.3, 136.4, 134.7, 132.5, 130.7, 129.7, 129.2, 129.1, 128.9, 127.8, 127.3, 61.9, 14.3. HRMS Calcd for $C_{16}H_{14}ClNNaO_2$ $[M+Na]^+$: 310.0605; Found: 310.0596.

(E)-(2-(1-(methoxyimino)ethyl)phenyl)(o-tolyl)methanone **(3i)**

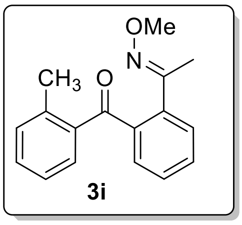

Colorless liquid. 16 mg, 60%. ^{1}H NMR (400 MHz, $CDCl_3$) δ 7.54-7.51 (m, 2H, ArH), 7.46-7.41 (m, 2H, ArH), 7.37-7.33 (m, 1H, ArH), 7.28 (s, 1H, ArH), 7.21-7.19 (m, 1H, ArH), 7.12 (t, J = 8 Hz, 1H ArH,), 3.80 (s, 3H, CH_3), 2.55 (s, 3H, CH_3), 1.95 (s, 3H, CH_3). ^{13}C NMR (100 MHz, $CDCl_3$) δ 199.3, 155.9, 140.0, 139.2, 138.2, 137.7, 131.7, 131.4, 130.9, 130.5, 130.1, 128.7, 128.4, 125.2, 61.8, 21.2, 15.6. HRMS Calcd for $C_{17}H_{17}NNaO_2$ $[M+Na]^+$: 290.1151; Found: 290.1152.

(*E*)-(2-bromophenyl)(2-(1-(methoxyimino)ethyl)phenyl)methanone **(3j)**

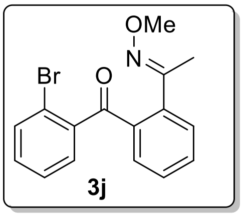

Colorless liquid. 6.9 mg, 21%. ^{1}H NMR (400 MHz, $CDCl_3$) δ 7.64-7.62 (m, 1H, ArH), 7.57-7.54 (m, 2H, ArH), 7.43 (t, J = 8 Hz, 2H, ArH), 7.38-7.36 (m, 1H, ArH), 7.35-7.27 (m, 2H, ArH), 3.90 (s, 3H, CH_3), 2.01 (s, 3H, CH_3). ^{13}C NMR (100 MHz, $CDCl_3$) δ 197.9, 172.6, 154.2, 133.9, 132.7, 130.3, 129.5, 129.4, 129.1, 128.6, 128.4, 127.8, 61.8, 14.5. HRMS Calcd for $C_{16}H_{14}BrNNaO_2$ $[M+Na]^+$: 354.0100; Found: 354.0113.

(*E*)-benzo[d][1,3]dioxol-5-yl(2-(1-(methoxyimino)ethyl)phenyl)methanone **(3k)**

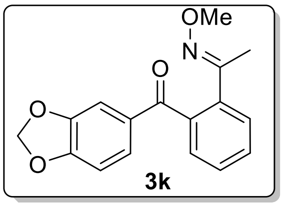

Colorless liquid. 13.9 mg, 47%. ^{1}H NMR (400 MHz, $CDCl_3$) δ 7.51-7.40 (m, 4H, ArH), 7.32 (s, 1H, ArH), 7.21-7.18 (m, 1H, ArH), 6.77-6.75 (d, 1H, ArH), 6.04 (s, 2H, CH_2), 3.72 (s, 3H, CH_3), 2.05 (s, 3H, CH_3). ^{13}C NMR (100 MHz, $CDCl_3$) δ 196.1, 154.2, 151.6, 148.1, 139.1, 136.4, 133.0, 130.1, 128.9, 128.6, 127.9, 126.3, 109.0, 107.8, 101.9, 61.9, 14.7. HRMS Calcd for $C_{17}H_{15}NNaO_4$ $[M+Na]^+$: 320.0893; Found: 320.0890.

(*E*)-(2-(1-(methoxyimino)ethyl)-5-methylphenyl)(phenyl)methanone **(4a)**

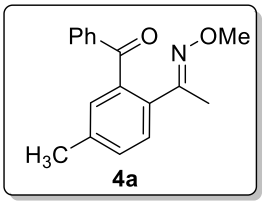

white solid, m.p. 98-100 ºC. 21.1 mg, 79%. ^{1}H NMR (400 MHz, $CDCl_3$) δ 7.71-7.69 (m, 2H, ArH), 7.53-7.49 (m, 1H, ArH), 7.41-7.37 (m, 3H, ArH), 7.34-7.31 (m, 1H, ArH), 7.28-

7.27 (m, 1H, ArH,), 3.65 (s, 3H, CH_3), 2.40 (s, 3H, CH_3), 2.00 (s, 3H, CH_3). ^{13}C NMR (100 MHz, $CDCl_3$) δ 197.9, 153.9, 138.9, 138.4, 133.6, 132.5, 130.9, 129.6, 129.3, 128.3, 127.6, 61.7, 21.3, 14.3. HRMS Calcd for $C_{17}H_{17}NNaO_2$ $[M+Na]^+$: 290.1151; Found: 290.1158.

(*E*)-(5-ethyl-2-(1-(methoxyimino)ethyl)phenyl)(phenyl)methanone **(4b)**

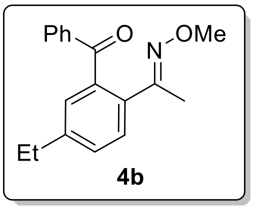

yellow liquid. 23.6 mg, 84%. ^{1}H NMR (400 MHz, $CDCl_3$) δ 7.72-7.70 (m, 2H, ArH), 7.53-7.49 (m, 1H, ArH), 7.42-7.30 (m, 5H, ArH), 3.66 (s, 3H, CH_3), 2.71 (q, J = 8 Hz, 2H, CH_2), 2.01 (s, 3H, CH_3), 1.26 (t, J = 8 Hz, 3H, CH_3). ^{13}C NMR (100 MHz, $CDCl_3$) δ 197.8, 155.1, 153.9, 138.5, 136.8, 136.1, 132.6, 129.5, 129.3, 128.3, 125.8, 125.0, 61.8, 35.1, 31.3, 15.2. HRMS Calcd for $C_{18}H_{19}NNaO_2$ $[M+Na]^+$: 304.1308; Found: 304.1316.

(*E*)-(5-isopropyl-2-(1-(methoxyimino)ethyl)phenyl)(phenyl)methanone **(4c)**

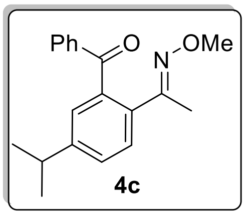

Colorless liquid. 25.9 mg, 88%. ^{1}H NMR (400 MHz, $CDCl_3$) δ 7.72- 7.70 (m, 2H, ArH), 7.53-7.49 (m, 1H, ArH), 7.43-7.37 (m, 4H, ArH), 7.34 (d, J = 2 Hz, 1H, ArH), 3.66 (s, 3H, CH_3), 3.00-2.93 (m, 1H, CH_3), 2.00 (s, 3H, CH_3), 1.27 (d, J = 8 Hz, 6H, 2×CH_3). ^{13}C NMR (100 MHz, $CDCl_3$) δ 198.1, 154.0, 149.8, 138.9, 138.4, 134.0, 132.6, 129.4, 128.4, 128.3, 127.7, 127.3, 61.7, 34.0, 23.9, 14.4. HRMS Calcd for $C_{19}H_{21}NNaO_2$ $[M+Na]^+$: 318.1465; Found: 318.1469.

(*E*)-(5-(*tert*-butyl)-2-(1-(methoxyimino)ethyl)phenyl)(phenyl)methanone **(4d)**

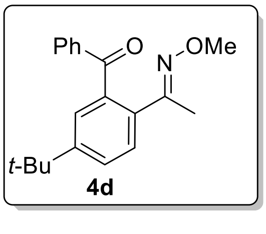

yellow liquid. 21.6 mg, 70%. ^{1}H NMR (400 MHz, $CDCl_3$) δ 7.72-7.70 (m, 2H, ArH), 7.56-7.53 (m, 1H, ArH), 7.52-7.49 (m, 2H, ArH), 7.42-7.38 (m, 3H, ArH), 3.66 (s, 3H, CH_3), 2.00 (s, 3H, CH_3), 1.34 (s, 9H, 3×CH_3). ^{13}C NMR (100 MHz, $CDCl_3$) δ 198.2, 154.0, 152.2, 138.6, 133.7, 132.6, 129.4, 128.3, 127.5, 127.3, 126.2, 61.7, 35.0,

31.3, 14.4. HRMS Calcd for $C_{20}H_{23}NNaO_2$ $[M+Na]^+$: 332.1621; Found: 332.1630.

(*E*)-(5-methoxy-2-(1-(methoxyimino)ethyl)phenyl)(phenyl)methanone **(4e)**

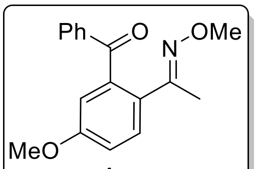

4e

Colorless liquid. 25.5 mg, 90%. ^{1}H NMR (400 MHz, $CDCl_3$) δ 7.72-7.69 (m, 2H, ArH), 7.53-7.49 (m, 1H, ArH), 7.44-7.37 (m, 3H, ArH), 7.06-7.03 (m, 1H, ArH), 6.99-6.98 (m, 1H, ArH), 3.85 (s, 3H, CH_3), 3.63 (s, 3H, CH_3), 1.99 (s, 3H, CH_3). ^{13}C NMR (100 MHz, $CDCl_3$) δ 197.4, 159.9, 153.5, 140.4, 138.2, 132.7, 129.3, 129.1, 128.8, 128.4, 116.1, 114.1, 61.7, 55.7, 14.3. HRMS Calcd for $C_{17}H_{17}NNaO_3$ $[M+Na]^+$: 306.1101; Found: 306.1113.

(*E*)-(2-(1-(methoxyimino)ethyl)-4-methylphenyl)(phenyl)methanone **(4f)**

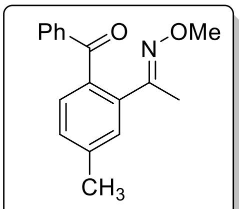

4f

white solid, m.p. 96-98 ºC. 18.9 mg, 71%. ^{1}H NMR (400 MHz, $CDCl_3$) δ 8.12 (d, J = 8 Hz, 1H, ArH), 7.71 (d, J = 8 Hz, 2H, ArH), 7.51-7.48 (m, 2H, ArH), 7.41-7.38 (m, 2H, ArH), 7.29 (s, 1H, ArH), 3.70 (s, 3H, CH_3), 2.45 (s, 3H, CH_3), 2.00 (s, 3H, CH_3). ^{13}C NMR (101 MHz, CDCl3) δ 197.75, 154.74, 140.83, 138.55, 136.97, 136.10, 133.88, 132.56, 130.34, 129.51, 128.63, 128.32, 61.80, 21.60, 14.96. HRMS Calcd for $C_{17}H_{18}NO_2$ $[M+H]^+$: 268.1332; Found: 268.1337.

(*E*)-(4-(tert-butyl)-2-(1-(methoxyimino)ethyl)phenyl)(phenyl)methanone **(4g)**

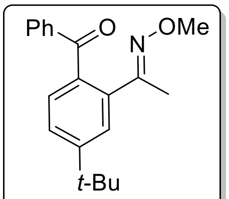

4g

yellow liquid. 24.7 mg, 80%. ^{1}H NMR (400 MHz, $CDCl_3$) δ 7.74-7.72 (m, 2H, ArH), 7.54-7.49 (m, 3H, ArH), 7.44-7.38 (m, 3H, ArH,), 3.70 (s, 3H, CH_3), 2.03 (s, 3H, CH_3), 1.38 (s, 9H, 3×CH_3,). ^{13}C NMR (100 MHz, $CDCl_3$) δ 198.0, 154.0, 145.2, 139.0, 138.4, 133.9, 132.6, 129.8, 129.4, 128.6, 128.3, 127.7, 61.7, 28.7, 15.4, 14.4. HRMS Calcd for $C_{20}H_{23}NNaO_2$ $[M+Na]^+$: 332.1621; Found: 332.1632.

(E)-(4-methoxy-2-(1-(methoxyimino)ethyl)phenyl)(phenyl)methanone **(4h)**

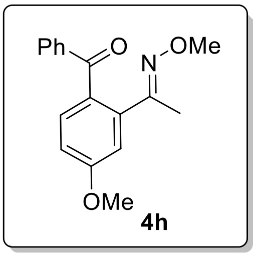

Colorless liquid. 22.9 mg, 81%. ^{1}H NMR (400 MHz, $CDCl_3$) δ 7.70-7.68 (m, 2H, ArH), 7.53-7.48 (m, 2H, ArH), 7.40 (t, J =8 Hz, 2H, ArH), 6.98-6.94 (m, 2H, ArH), 3.89 (s, 3H, CH_3), 3.73 (s, 3H, CH_3), 1.99 (s, 3H, CH_3). ^{13}C NMR (100 MHz, $CDCl_3$) δ 197.0, 161.4, 155.0, 139.4, 138.9, 132.4, 131.8, 131.3, 130.3, 129.5, 128.3, 114.1, 113.5, 61.9, 55.7, 15.3. HRMS Calcd for $C_{17}H_{18}NO_3$ $[M+H]^+$: 284.1281; Found: 284.1289.

(E)-(5-fluoro-2-(1-(methoxyimino)ethyl)phenyl)(phenyl)methanone **(4i)**

yellow solid, m.p. 96-99 ºC. 16.5 mg, 61%. ^{1}H NMR (400 MHz, CDCl3) δ 7.71-7.69

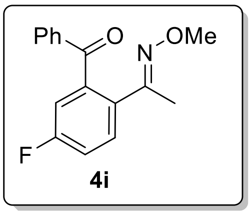

(ArH, m, 2H), 7.56 - 7.51 (ArH, m, 1H), 7.50-7.47 (ArH, m, 1H), 7.43-7.39 (ArH, m, 2H), 7.24- 7.16 (ArH, m, 2H). 3.65 (CH_3, s, 3H), 2.01 (CH_3, s, 3H). ^{19}F NMR (377 MHz, $CDCl_3$) δ -111.72. ^{13}C NMR (100 MHz, $CDCl_3$) δ 196.1, 162.6 (d, J_{C-F}= 250 Hz), 153.1, 141.1 (d, J_{C-F}= 6 Hz), 137.7, 134.5, 133.0, 132.6 (d, J_{C-F}= 3 Hz), 129.8 (d, J_{C-F}= 8 Hz), 129.4, 128.5, 117.1 (d, J_{C-F}= 22 Hz), 116.3 (d, J_{C-F}= 23 Hz), 61.8, 14.5. HRMS Calcd for $C_{16}H_{15}FNO_2$ $[M+H]^+$: 272.1081; Found: 272.1085.

(E)-(5-chloro-2-(1-(methoxyimino)ethyl)phenyl)(phenyl)methanone **(4j)**

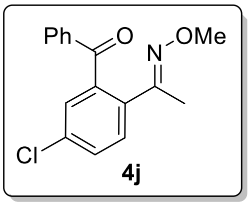

Colorless liquid. 16.1 mg, 56%. ^{1}H NMR (400 MHz, $CDCl_3$) δ 7.71-7.69 (m, 2H, ArH), 7.56-7.48 (m, 2H, ArH), 7.45- 7.39 (m, 4H, ArH), 3.64 (s, 3H, CH_3), 2.01 (s, 3H, CH_3). ^{13}C NMR (100 MHz, $CDCl_3$) δ 196.1, 152.9, 140.5, 137.7, 135.0, 134.8, 133.0, 130.2, 129.4, 129.0, 129.0, 128.5, 61.9, 14.2. HRMS Calcd for $C_{16}H_{15}ClNO_2$ $[M+H]^+$: 288.0786; Found: 288.0787.

(*E*)-(5-bromo-2-(1-(methoxyimino)ethyl)phenyl)(phenyl)methanone (**4k)**

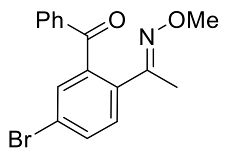

4k

yellow liquid. 15.3 mg, 46%. ^{1}H NMR (400 MHz, $CDCl_3$) δ 7.71-7.68 (m, 2H, ArH), 7.66-7.64 (m, 1H, ArH), 7.59 (d, *J* = 4 Hz, 1H, ArH), 7.55-7.51 (m, 1H, ArH), 7.43-7.36 (m, 3H, ArH), 3.64 (s, 3H, CH_3), 2.01 (s, 3H, CH_3). ^{13}C NMR (100 MHz, $CDCl_3$) δ 196.0, 153.0, 140.7, 137.7, 135.2, 133.2, 133.0, 131.9, 129.4, 129.2, 128.5, 123.0, 61.9, 14.2. HRMS Calcd for $C_{16}H_{14}BrNNaO_2$ $[M+Na]^+$: 354.0100; Found: 354.0113.

(*E*)-(4-bromo-2-(1-(methoxyimino)ethyl)phenyl)(phenyl)methanone **(4l)**

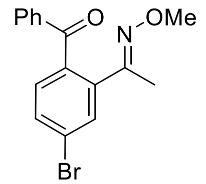

4l

Colorless liquid. 11.9 mg, 36%. ^{1}H NMR (400 MHz, $CDCl_3$) δ 7.70-7.68 (m, 2H, ArH), 7.64 (m, 1H, ArH), 7.61- 7.58 (m, 1H, ArH), 7.53 (ArH, m, 1H), 7.41 (ArH, t, 2H), 7.34 (d, 1H, ArH), 3.68 (s, 3H, CH_3), 2.00 (s, 3H, CH_3). ^{13}C NMR (100 MHz, $CDCl_3$) δ 196.7, 152.9, 138.5, 137.9, 137.7, 132.9, 131.7, 130.9, 130.7, 129.4, 128.5, 124.6, 62.0, 14.4. HRMS Calcd for $C_{16}H_{14}BrNNaO_2$ $[M+Na]^+$: 354.0100; Found: 354.0108.

(*E*)-(3-fluoro-2-(1-(methoxyimino)ethyl)phenyl)(phenyl)methanone **(4m)**

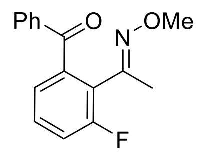

4m

Colorless liquid. 6.2 mg, 23%. ^{1}H NMR (400 MHz, $CDCl_3$) δ 7.71 (d, *J* = 8 Hz, 2H, ArH), 7.56-7.52 (m, 1H, ArH), 7.44-7.40 (m, 3H, ArH), 7.30-7.22 (m, 2H, ArH), 3.68 (s, 3H, CH_3), 2.03-2.04 (s, 3H, CH_3). ^{19}F NMR (377 MHz, $CDCl_3$) δ -114.27. ^{13}C NMR (100 MHz, $CDCl_3$) δ 196.3 (d, $J_{C\text{-}F}$= 2 Hz), 160.8 (d, $J_{C\text{-}F}$= 249 Hz), 151.8, 141.8 (d, $J_{C\text{-}F}$= 2 Hz), 138.0, 133.0, 130.1 (d, $J_{C\text{-}F}$= 9 Hz), 129.6, 128.4, 124.7 (d, $J_{C\text{-}F}$= 3 Hz), 118.1 (d, $J_{C\text{-}F}$= 22 Hz), 61.9, 16.2 (d, $J_{C\text{-}F}$= 4 Hz). HRMS Calcd for $C_{16}H_{15}FNO_2$ $[M+H]^+$: 272.1081; Found: 272.1093.

(*E*)-(3-bromo-2-(1-(methoxyimino)ethyl)phenyl)(phenyl)methanone **(4n)**

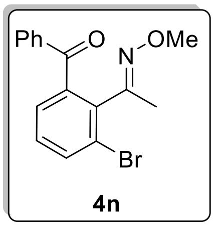

white solid, m.p. 108-110 ºC. 13.9 mg, 42%. ^{1}H NMR (400 MHz, $CDCl_3$) δ 7.71 (d, *J* = 8 Hz, 2H, ArH), 7.54-7.46 (m, 4H, ArH), 7.40 (t, *J* = 8 Hz, 2H ArH), 3.68 (CH_3, s, 3H), 2.03 (CH_3, s, 3H). ^{13}C NMR (100 MHz, $CDCl_3$) δ 197.8, 154.1, 139.0, 138.3, 136.5, 132.7, 130.3, 129.4, 129.1, 128.7, 128.4, 127.8, 61.8, 14.5. HRMS Calcd for $C_{16}H_{14}BrNNaO_2$ $[M+Na]^+$:354.0100; Found: 354.0096.

(*E*)-(4-fluoro-2-(1-(methoxyimino)ethyl)phenyl)(phenyl)methanone **(4o)**

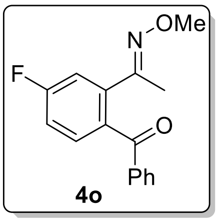

white solid, m.p. 154-155 ºC. 16.3 mg, 60%. ^{1}H NMR (400 MHz, $CDCl_3$) δ 7.70-7.67 (m, 2H, ArH), 7.55-7.47 (m, 2H, ArH), 7.43-7.39 (m, 2H, ArH), 7.21-7.13 (m, 2H, ArH), 3.69 (s, 3H, CH_3), 2.00 (s, 3H, CH_3). ^{19}F NMR (377 MHz, $CDCl_3$) δ -109.35. ^{13}C NMR (100 MHz, $CDCl_3$) δ 196.6, 163.7 (d, $J_{C\text{-}F}$= 249 Hz), 153.3, 139.3 (d, $J_{C\text{-}F}$= 8 Hz), 138.2, 135.0 (d, $J_{C\text{-}F}$= 3 Hz), 132.8, 131.5 (d, $J_{C\text{-}F}$= 8 Hz), 129.4, 128.5, 115.6 (d, $J_{C\text{-}F}$= 21 Hz), 115.1 (d, $J_{C\text{-}F}$= 23 Hz), 62.0, 14.5. HRMS Calcd for $C_{16}H_{15}FNO_2$ $[M+H]^+$: 272.1081; Found: 272.1091.

(*E*)-(2-fluoro-6-(1-(methoxyimino)ethyl)phenyl)(phenyl)methanone **(4o')**

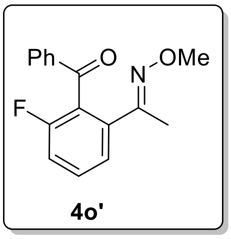

white solid, m.p. 146-148 ºC. 4.9 mg, 18%. ^{1}H NMR (400 MHz, $CDCl_3$) δ 7.81-7.79 (m, 2H, ArH), 7.56-7.52 (m, 1H, ArH), 7.48-7.41 (m, 3H, ArH), 7.33-7.31 (m, 1H, ArH), 7.18-7.14 (m, 1H, ArH), 3.56 (s, 3H, CH_3,), 2.08 (s, 3H, CH_3). ^{19}F NMR (377 MHz, $CDCl_3$) δ -114.54. ^{13}C NMR (100 MHz, $CDCl_3$) δ 193.4, 159.8 (d, $J_{C\text{-}F}$= 246 Hz), 152.4, 138.2, 137.6, 133.1, 130.9 (d, $J_{C\text{-}F}$= 9 Hz), 129.2, 128.6, 123.5 (d, $J_{C\text{-}F}$= 3 Hz), 116.4 (d, $J_{C\text{-}F}$= 22Hz), 61.8, 13.8. HRMS Calcd for $C_{16}H_{15}FNO_2$ $[M+H]^+$: 272.1081; Found: 272.1092.

(*E*)-(2-(1-(methoxyimino)ethyl)-5-(trifluoromethyl)phenyl)(phenyl)methanone **(4p)**

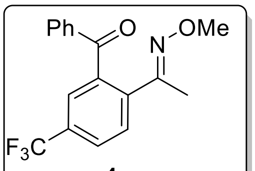

4p

Colorless liquid. 12.8 mg, 40%. ^{1}H NMR (400 MHz, CDCl3) δ 7.78 (d, *J* = 8 Hz, 1H, ArH), 7.72- 7.68 (m, 3H, ArH), 7.63 (d, *J* = 8 Hz, 1H, ArH), 7.57-7.54 (m, 1H, ArH), 7.45-7.41 (m, 2H, ArH), 3.67 (s, 3H, CH_3), 2.05 (s, 3H, CH_3). ^{19}F NMR (377 MHz, $CDCl_3$) δ -62.76. ^{13}C NMR (100 MHz, $CDCl_3$) δ 196.3, 152.8, 139.7 (q, $J_{C\text{-}F}$= 10 Hz) 137.6, 133.2, 131.4, 130.7, 129.4, 128.4 (q, $J_{C\text{-}F}$= 34 Hz), 127.0 (q, $J_{C\text{-}F}$= 3 Hz), 125.9 (q, $J_{C\text{-}F}$= 4 Hz), 122.5 (q, $J_{C\text{-}F}$= 263 Hz), 62.1, 14.2. HRMS Calcd for $C_{17}H_{14}F_3NNaO_2$ $[M+Na]^+$: 344.0869; Found: 344.0860.

(*E*)-(8-(methoxyimino)-5,6,7,8-tetrahydronaphthalen-1-yl)(phenyl)methanone **(4q)**

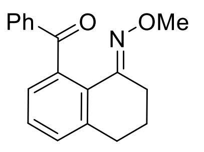

4q

Colorless liquid. 16.5 mg, 59%. ^{1}H NMR (400 MHz, $CDCl_3$) δ 7.72-7.70 (m, 2H, ArH), 7.50-7.46 (m, 1H, ArH), 7.39-7.33 (m, 3H, ArH), 7.28 (m,1H, ArH), 7.21-7.19 (m, 1H, ArH), 3.54 (s, 3H, CH_3), 2.80 (t, *J* =8 Hz, 2H, CH_2), 2.57 (t, *J* =8 Hz, 2H, CH_2), 1.88-1.82 (m, 2H, CH_3). ^{13}C NMR (100 MHz, $CDCl_3$) δ 198.0, 151.8, 140.7, 138.8, 138.4, 132.2, 129.7, 129.1, 128.9, 128.6, 128.3, 126.5, 61.7, 30.5, 24.3, 21.2. HRMS Calcd for $C_{18}H_{17}NNaO_2$ $[M+Na]^+$: 302.1151; Found: 302.1147.

(*E*)-(4-(methoxyimino)chroman-5-yl)(phenyl)methanone (**4r)**

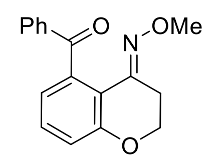

4r

Colorless liquid. 20 mg, 71%. ^{1}H NMR (400 MHz, CDCl3) δ 7.76-7.74 (m, 2H, ArH), 7.52-7.48 (m, 1H, ArH), 7.41-7.37 (m, 2H, ArH), 7.35-7.31 (m, 1H, ArH), 7.03-7.01 (m, 1H, ArH), 6.91-6.89 (m, 1H, ArH), 4.22 (t, *J* = 8 Hz, 2H, CH_2), 3.52 (s, 3H, CH_3), 2.80 (t, *J* = 8 Hz, 2H, CH_2). ^{13}C NMR (100 MHz, $CDCl_3$) δ 197.4, 157.0, 146.1, 139.0, 138.0, 132.5, 130.5, 129.2, 128.3, 121.1, 118.9, 116.4, 65.1, 61.9, 24.1. HRMS Calcd for $C_{17}H_{16}NO_3$ $[M+H]^+$: 282.1125; Found: 282.1135.

(*E*)-(2-((methoxyimino)(phenyl)methyl)phenyl)(phenyl)methanone **(4s)**

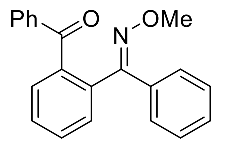
4s

white solid, m.p. 79-81 ºC. 22.1 mg, 70%. ^{1}H NMR (400 MHz, $CDCl_3$) δ 7.74-7.72 (m, 2H, ArH), 7.51-7.43 (m, 4H, ArH), 7.40-7.35 (m, 2H, ArH), 7.32-7.27 (m, 6H, ArH), 3.69 (s, 3H, CH_3). ^{13}C NMR (100 MHz, $CDCl_3$) δ 197.6, 154.9, 140.1, 138.1, 136.0, 132.8, 132.7, 130.3, 129.9, 129.7, 129.5, 129.3, 129.0, 129.0, 128.3, 128.0, 62.3. HRMS Calcd for $C_{21}H_{17}NNaO_2$ $[M+Na]^+$: 338.1151; Found: 338.1163.

(*E*)-(2-((methoxyimino)(*p*-tolyl)methyl)-5-methylphenyl)(phenyl)methanone **(4t)**

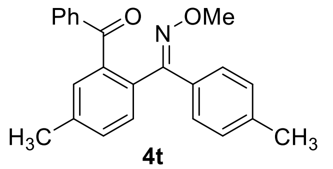
4t

white solid, m.p. 106-108 ºC. 19.9 mg, 58%. ^{1}H NMR (400 MHz, $CDCl_3$) δ 7.66-7.63 (m, 2H, ArH), 7.41-7.37 (m, 1H, ArH), 7.30-7.26 (m, 2H, ArH), 7.21 (s, 1H, ArH), 7.18-7.15 (m, 1H, ArH), 7.09-7.08 (m, 3H, ArH), 7.03 (d, *J* = 8 Hz, 2H, ArH,), 3.59 (s, 3H, CH_3), 2.33 (s, 3H, CH_3), 2.24 (s, 3H, CH_3). ^{13}C NMR (100 MHz, $CDCl_3$) δ 197.9, 154.8, 140.1, 139.4, 139.3, 138.2, 133.3, 132.6, 130.6, 130.2, 130.0, 129.7, 129.6, 129.5, 128.7, 128.2, 62.2, 21.5, 21.3. HRMS Calcd for $C_{23}H_{21}NNaO_2$ $[M+Na]^+$: 366.1465; Found: 366.1460.

(*E*)-(5-methoxy-2-((methoxyimino)(4-methoxyphenyl)methyl)phenyl)(phenyl)methanone (**4u)**

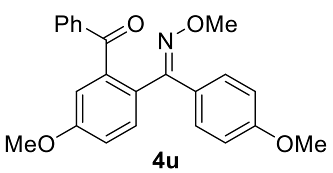
4u

Colorless liquid. 19.1 mg, 51%. ^{1}H NMR (400 MHz, $CDCl_3$) δ 7.72 (d, *J* = 2 Hz, 2H, ArH), 7.48 (m, *J* = 8 Hz, 1H, ArH), 7.36 (m, *J* = 8 Hz, 2H ArH,), 7.26-7.21 (m, 3H, ArH), 7.03-6.96 (m, 2H, ArH), 6.81 (d, *J* = 8 Hz, 2H, ArH), 3.85 (s, 3H, CH_3), 3.79 (s, 3H, CH_3,), 3.68 (s, 3H, CH_3). ^{13}C NMR (100 MHz, $CDCl_3$) δ 197.3, 160.2, 154.3, 141.6, 137.9, 132.8, 131.8, 131.6, 129.5, 128.8, 128.6, 128.2, 125.3, 115.8, 114.2, 113.3, 62.1, 55.7, 55.4. HRMS Calcd for $C_{23}H_{21}NNaO_4$ $[M+Na]^+$: 398.1363; Found: 398.1352.

(*E*)-(5-fluoro-2-((4-fluorophenyl)(methoxyimino)methyl)phenyl)(phenyl)methanone **(4v)**

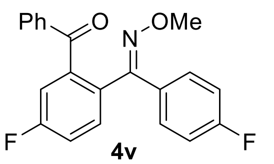

4v

Colorless liquid. 20 mg, 57%. ^{1}H NMR (400 MHz, $CDCl_3$) δ 7.63 (d, J = 2 Hz, 2H, ArH), 7.45 (t, J = 2 Hz, 1H, ArH,), 7.32 (t, J = 2 Hz, 2H, ArH), 7.24-7.17 (m, 3H, ArH), 7.14-7.06 (m, 2H, ArH), 6.91 (t, J = 2 Hz, 2H, ArH), 3.62 (s, 3H, CH_3). ^{19}F NMR (377 MHz, $CDCl_3$) δ -110.39, -110.93. ^{13}C NMR (100 MHz, $CDCl_3$) δ 195.8, 163.0 (d, J_{C-F}= 249 Hz), 162.8 (d, J_{C-F}= 251 Hz), 153.2, 142.2 (d, J_{C-F}= 6 Hz), 137.3, 133.3, 132.3 (d, J_{C-F}= 8 Hz), 132.1 (d, J_{C-F}= 4 Hz), 132.0 (d, J_{C-F}= 9 Hz), 130.4, 129.6, 128.4, 117.0 (d, J_{C-F}= 21 Hz), 116.3 (d, J_{C-F}= 23 Hz), 115.2 (d, J_{C-F}= 22 Hz), 62.4. HRMS Calcd for $C_{21}H_{15}F_2NNaO_2$ $[M+Na]^+$: 374.0963; Found: 374.0971.

methyl (*E*)-2-(4-benzoyl-3-((methoxyimino)(phenyl)methyl)phenyl)propanoate **(4w)** and methyl (*E*)-2-(3-((2-benzoylphenyl)(methoxyimino)methyl)phenyl)propanoate **(4w')**

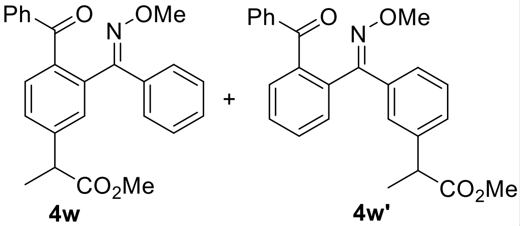

4w **4w'**

Colorless liquid. 23.3 mg, 58%. ^{1}H NMR (400 MHz, $CDCl_3$) δ 7.65-7.62 (m, 2H, ArH), 7.42-7.35 (m, 4H, ArH), 7.31-7.26 (m, 2H, ArH), 7.23-7.17 (m, 4H, ArH), 7.14-7.10 (m, 1H, ArH), 3.68-3.63 (m, 1H, CH), 3.62 (s, 3H, CH_3), 3.57 (d, J = 4 Hz, 3H, CH_3), 1.41-1.35 (m, 3H, CH_3). ^{13}C NMR (100 MHz, $CDCl_3$) δ 197.5, 197.2, 174.7, 174.4, 154.8, 154.6, 140.2, 140.1, 138.1, 137.9, 132.8, 132.7, 130.2, 129.9, 129.8, 129.7, 129.6, 129.5, 129.4, 129.4, 129.0, 129.0, 128.7, 128.3, 128.3, 128.2, 128.0, 127.9, 62.3, 52.2, 52.1, 45.3, 45.3, 18.6, 18.6. HRMS Calcd for $C_{25}H_{24}NO_4$ $[M+H]^+$: 402.1705; Found: 402.1696.

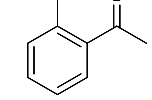

5

1-(2-benzoylphenyl)ethan-1-one **(5)**

white solid, m.p. 95 - 97 ºC. 30.9 mg, 69%. ^{1}H NMR (400 MHz, $CDCl_3$) δ 7.88-7.86 (m, 1H, ArH), 7.74-7.72 (m, 2H, ArH), 7.63-

7.55 (m, 2H, ArH), 7.53-7.49 (m, 1H, ArH), 7.41-7.37 (m, 3H, ArH), 2.49 (s, 3H, CH_3). ^{13}C NMR (100 MHz, $CDCl_3$) δ 198.5, 197.8, 140.8, 137.5, 137.2, 132.9, 132.2, 129.8, 129.3, 129.2, 128.4, 128.2, 27.4. HRMS Calcd for $C_{15}H_{12}NaO_2$ $[M+Na]^+$: 247.0730; Found: 247.0726.

1,2-phenylenebis(phenylmethanone) **(6)**

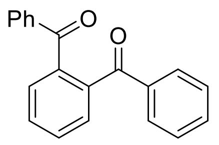

6

white solid, m.p. 144 - 146 ºC. 50.9 mg, 89%. ^1H NMR (400 MHz, $CDCl_3$) δ 7.72-7.70(m, 4H, ArH), 7.60(s, 4H, ArH), 7.51-7.47(m, 2H, ArH), 7.38-7.34(m, 4H, ArH). ^{13}C NMR (100 MHz, $CDCl_3$) δ 196.6, 140.0, 137.1, 133.0, 130.4, 129.8, 129.7, 128.3. HRMS Calcd for $C_{20}H_{14}NaO_2$ $[M+Na]^+$: 309.0886; Found: 309.0889.

1-methyl-4-phenylphthalazine **(7)**

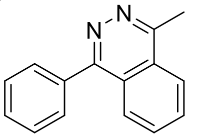

7

white solid, m.p. 125-126 ºC. 40 mg, 91%. ^1H NMR (400 MHz, $CDCl_3$) δ 8.13 (d, J = 8 Hz, 1H, ArH), 8.04 (d, J = 2 Hz, 1H, ArH), 7.92-7.88 (m, 1H, ArH), 7.84-7.80 (m, 1H, ArH), 7.73-7.71 (m, 2H, ArH), 7.55-7.53 (m, 3H, ArH), 3.05 (s, 1H, CH_3). ^{13}C NMR (100 MHz, $CDCl_3$) δ 159.3, 156.6, 136.6, 132.0, 132.0, 130.2, 129.2, 128.6, 126.9, 126.7, 125.2, 124.7, 20.0. HRMS Calcd for $C_{15}H_{12}N_2Na$ $[M+Na]^+$: 243.0893; Found: 243.0885.

1,4-diphenylphthalazine **(8)**

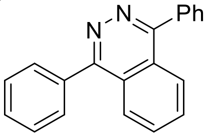

8

white solid, m.p. 195-196 ºC. 54.1 mg, 96%. ^1H NMR (400 MHz, $CDCl_3$) δ 8.15-8.13(m, 2H, ArH), 7.86 - 7.81(m, 6H, ArH), 7.61-7.56(m, 6H, ArH).^{13}C NMR (100 MHz, $CDCl_3$) δ 159.2, 136.5, 132.1, 130.3, 129.4, 128.7, 126.7, 126.0. HRMS Calcd for $C_{20}H_{14}N_2Na$ $[M+Na]^+$: 305.1049; Found: 305.1059.

3-phenyl-1H-inden-1-one **(9)**

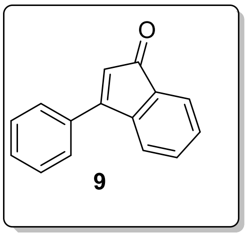

Yellow solid. m.p. 97 - 98 ºC. 29.3 mg, 71%. ^{1}H NMR (400 MHz, $CDCl_3$) δ 7.60-7.58 (m, 2H, ArH), 7.47-7.43 (m, 4H, ArH), 7.32-7.30 (m, 2H, ArH), 7.26-7.22 (m, 1H, ArH), 5.93 (s, 1H, CH). ^{13}C NMR (100 MHz, $CDCl_3$) δ 203.9, 158.4, 145.4, 136.0, 135.9, 129.6, 128.6, 127.5, 125.4, 125.2, 123.3, 78.6, 56.2. HRMS Calcd for $C_{15}H_{10}NaO$ $[M+Na]^+$: 229.0624; Found: 229.0620.

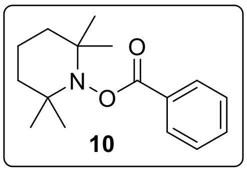

2,2,6,6-tetramethylpiperidin-1-yl benzoate **(10)**

Colorless liquid. ^{1}H NMR (400 MHz, $CDCl_3$) δ 8.09-8.06 (m, 2H, ArH), 7.60-7.55 (m, 1H, ArH), 7.48-7.44 (m, 2H, ArH), 1.78-1.71 (m, 3H, CH_2), 1.61-1.57 (m, 2H, CH_2), 1.48-1.42 (m, 1H, CH_2), 1.28 (s, 6H, 2×CH_3), 1.12 (s, 6H, 2×CH_3). ^{13}C NMR (100 MHz, $CDCl_3$) δ 166.6, 133.0, 129.9, 129.7, 128.6, 60.6, 39.2, 32.1, 21.0, 17.2. HRMS Calcd for $C_{16}H_{24}NO_2$ $[M+H]^+$: 262.1802; Found: 262.1791.

9. NMR spctra

^{1}H NMR of compound **3a**

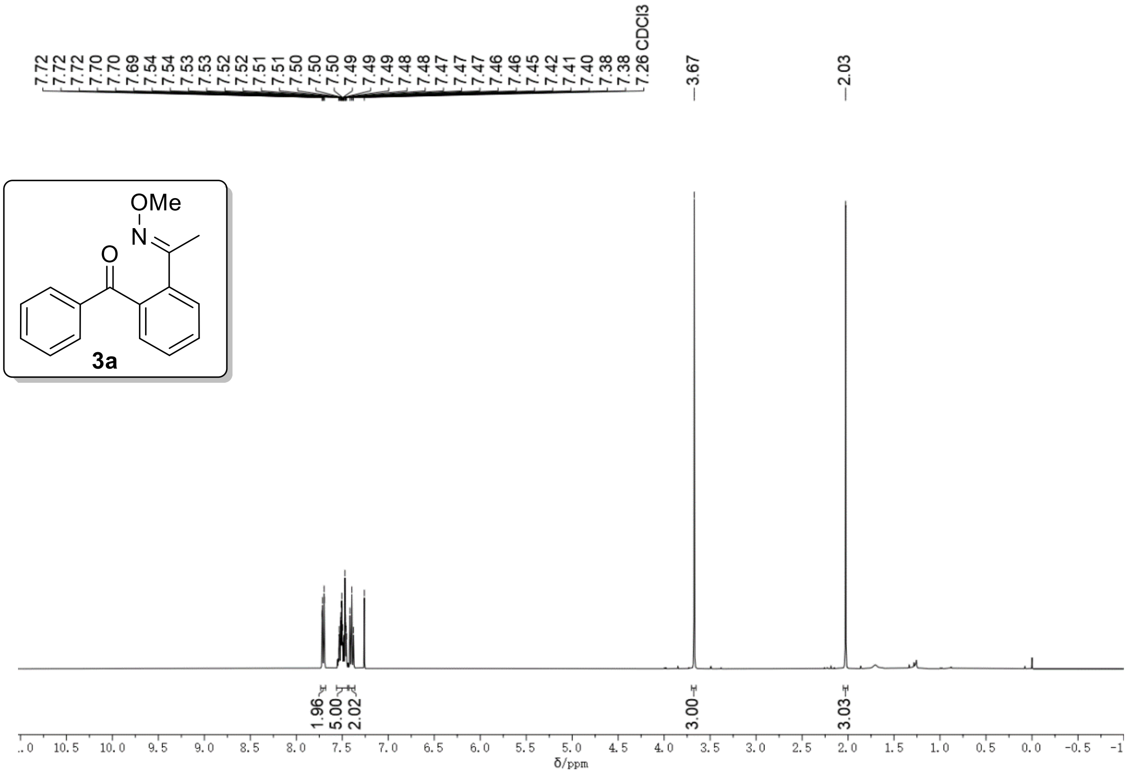

^{13}C NMR of compound **3a**

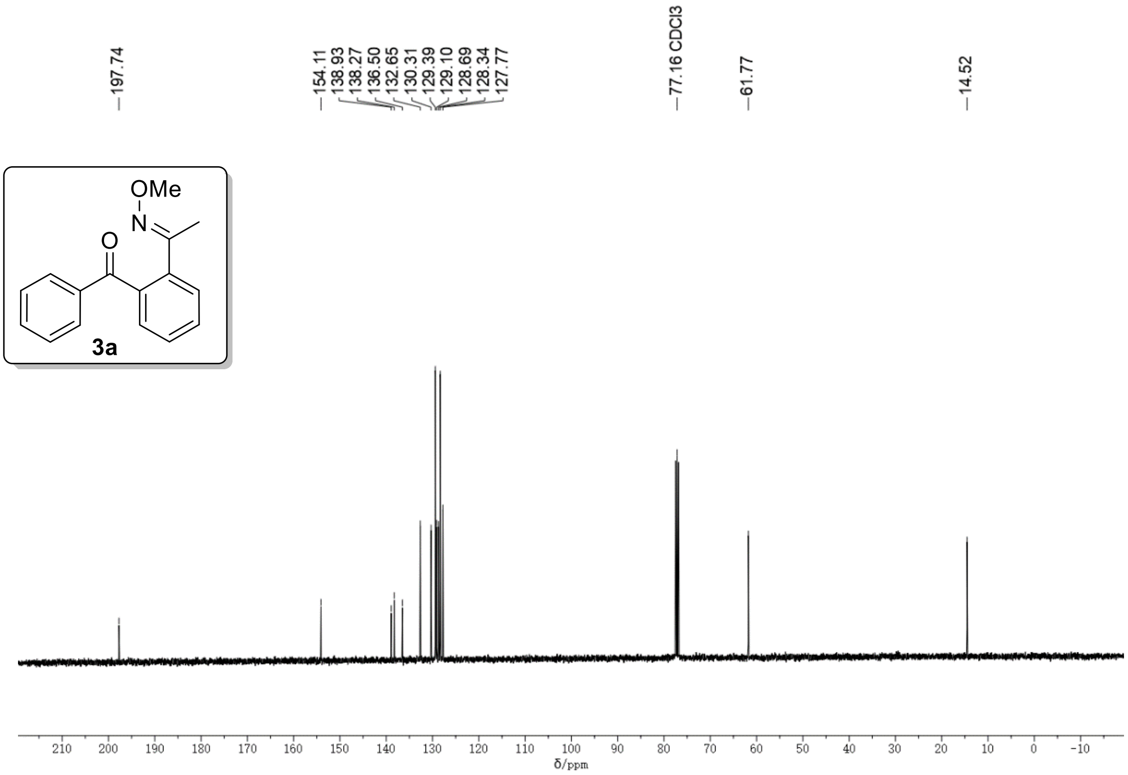

^{1}H NMR of compound **3b**

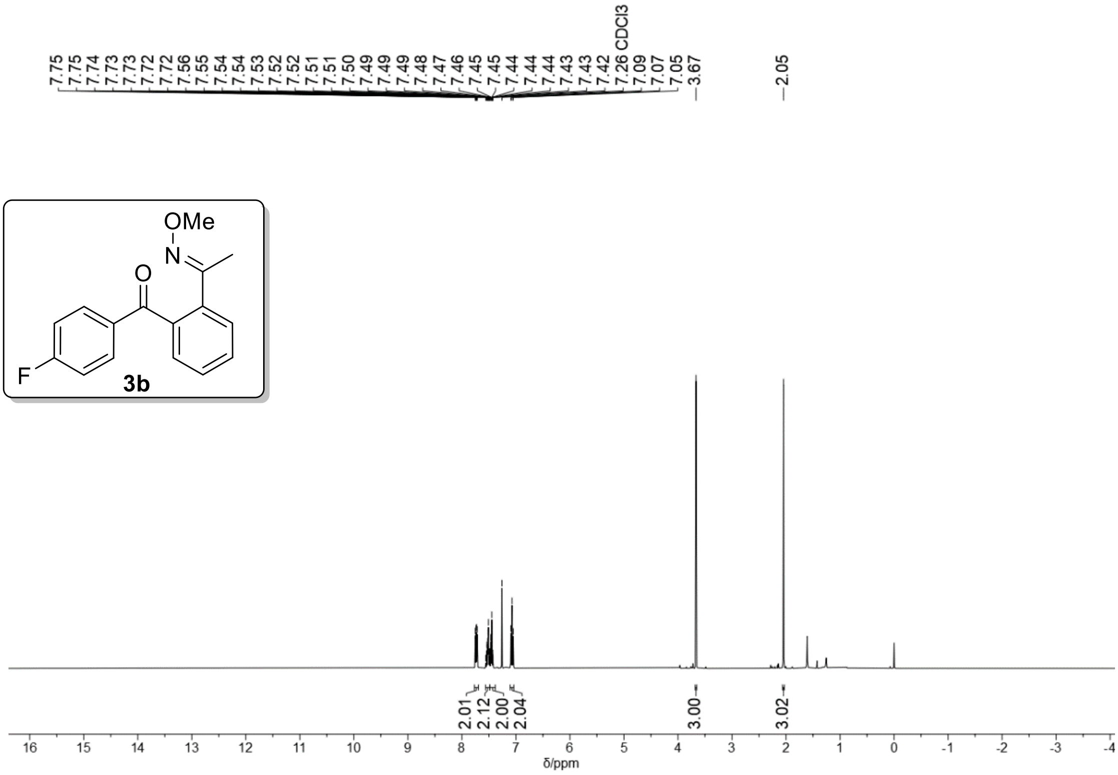

^{19}F NMR of compound **3b**

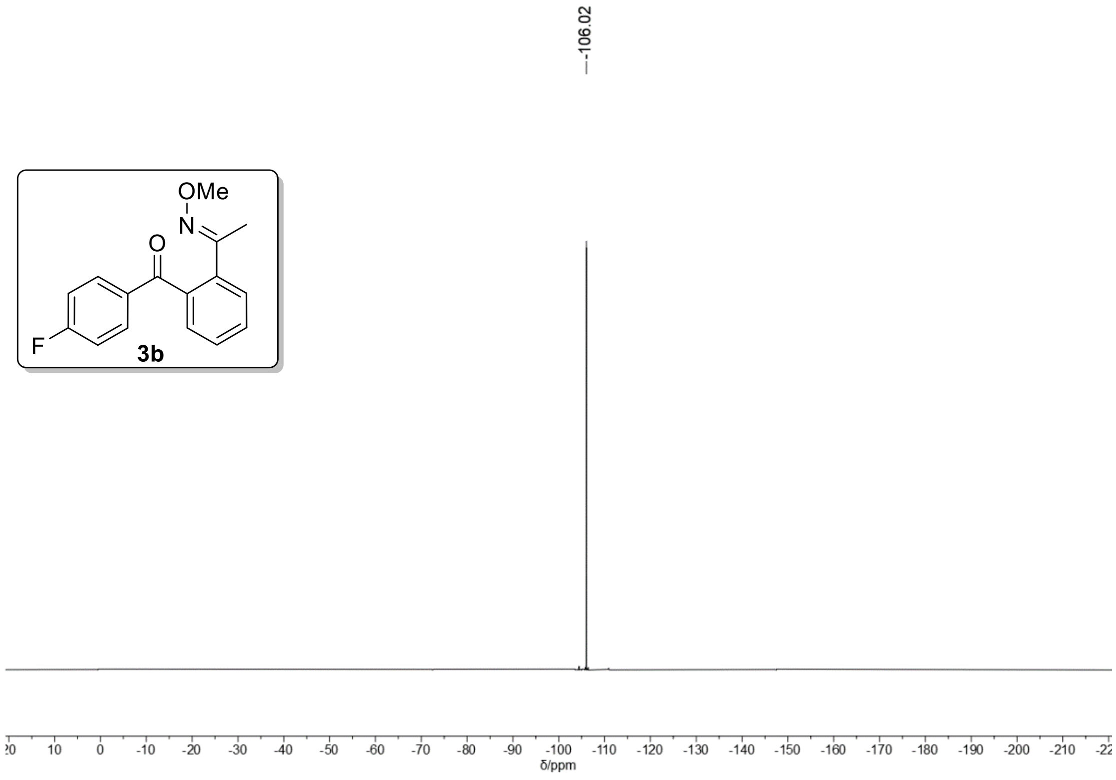

^{13}C NMR of compound **3b**

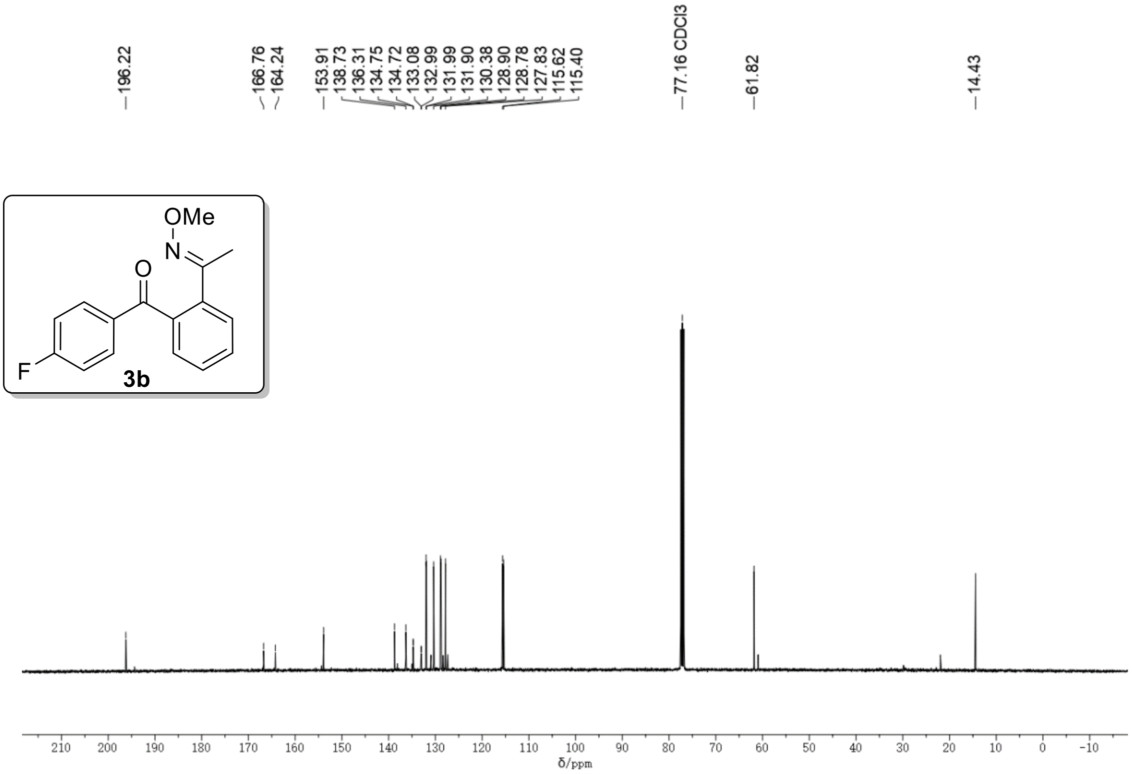

^{1}H NMR of compound **3c**

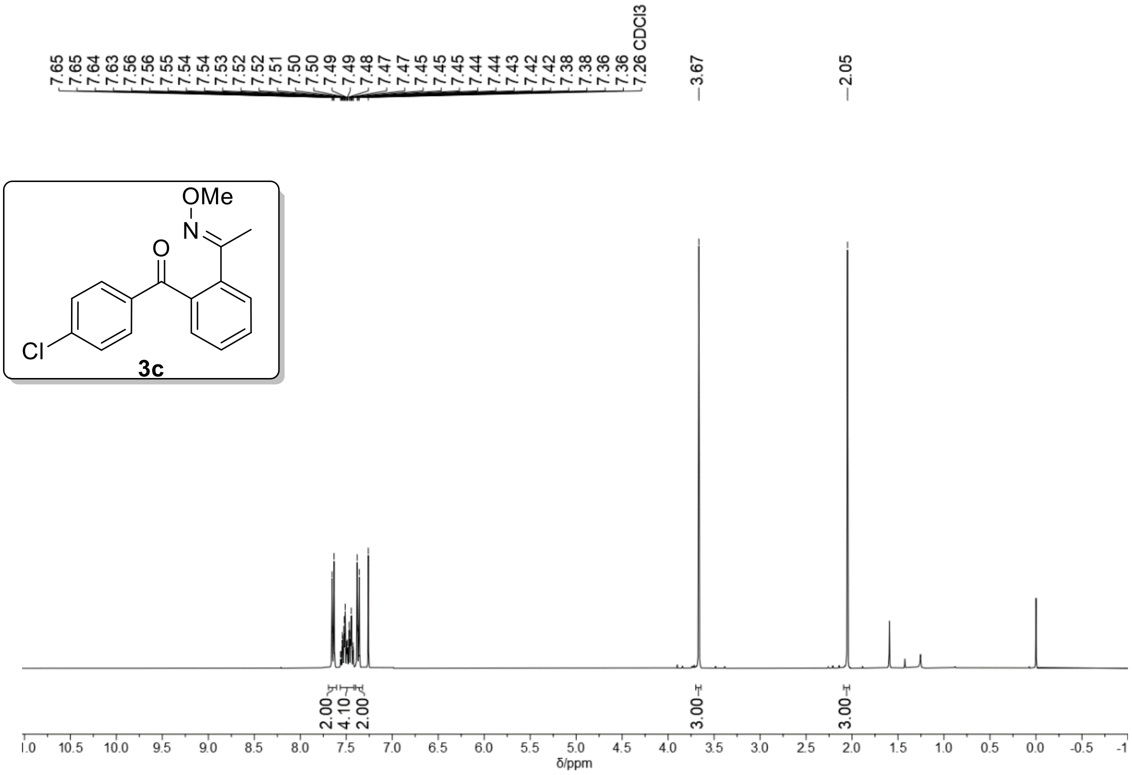

^{13}C NMR of compound **3c**

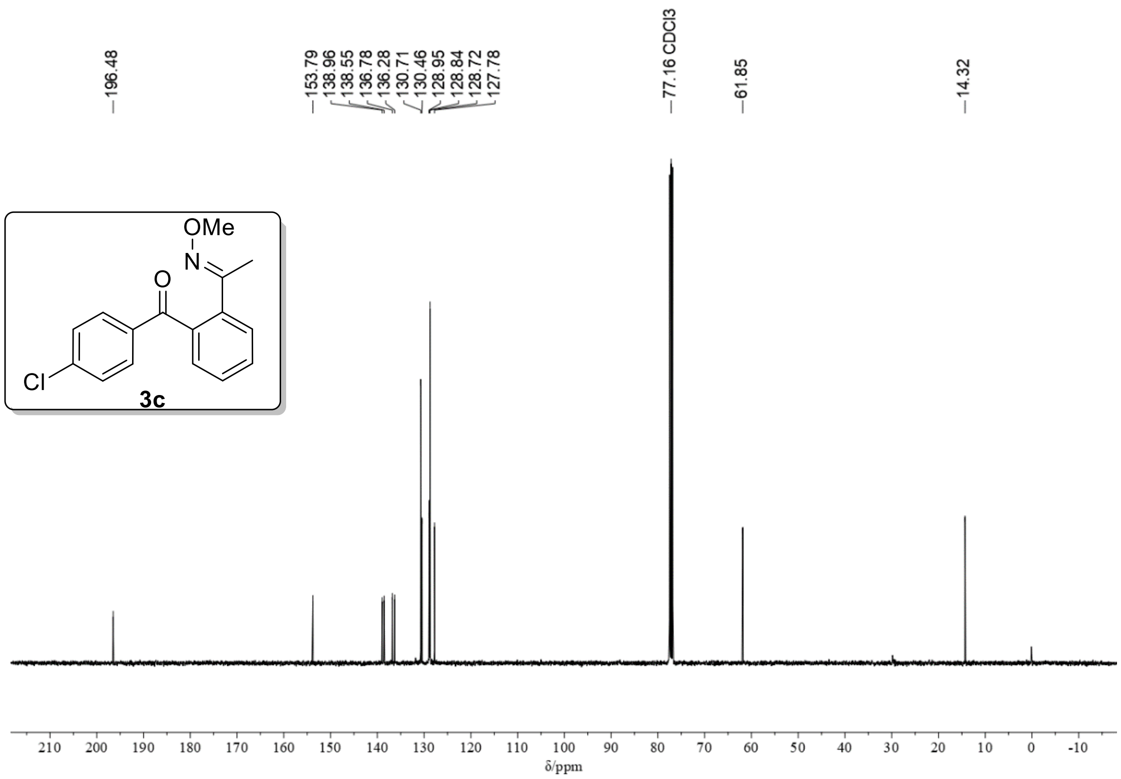

^{1}H NMR of compound **3d**

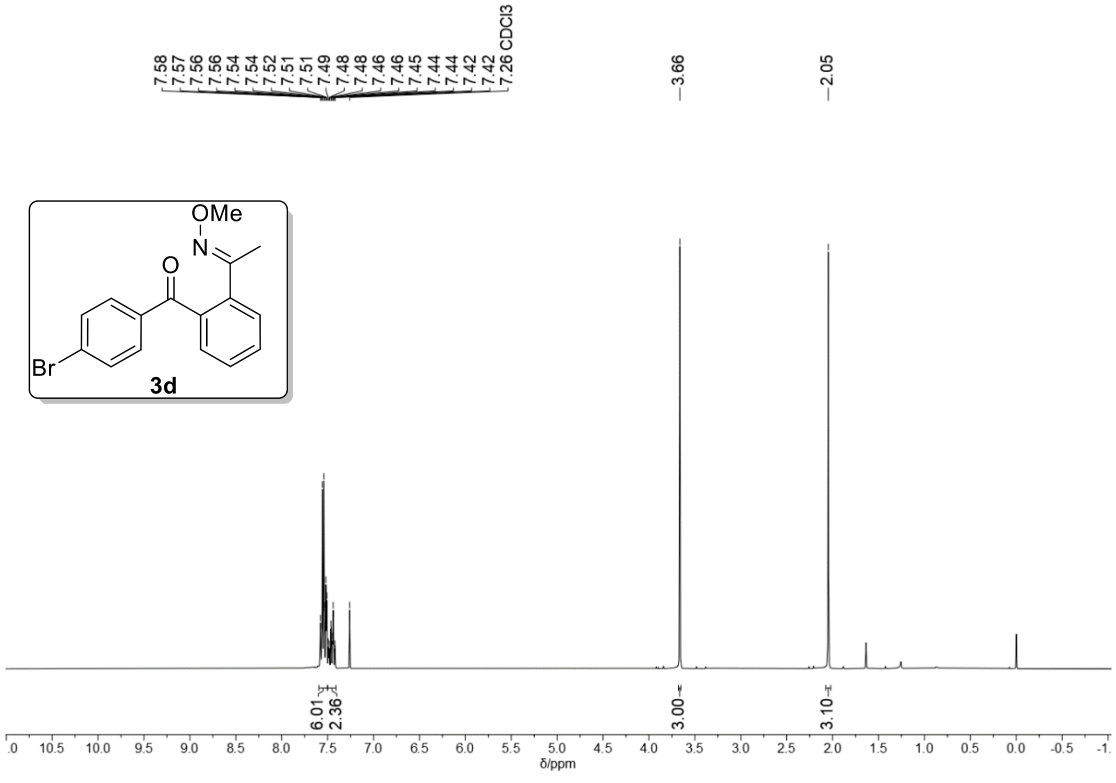

^{13}C NMR of compound **3d**

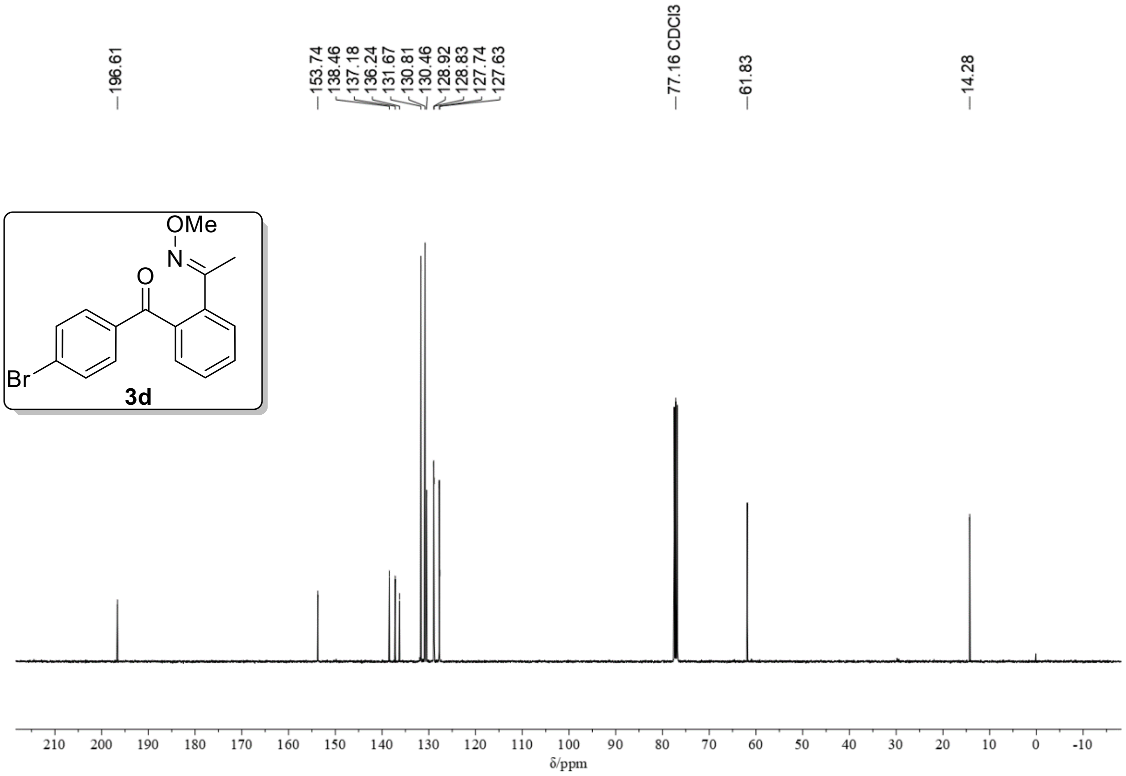

^{1}H NMR of compound **3e**

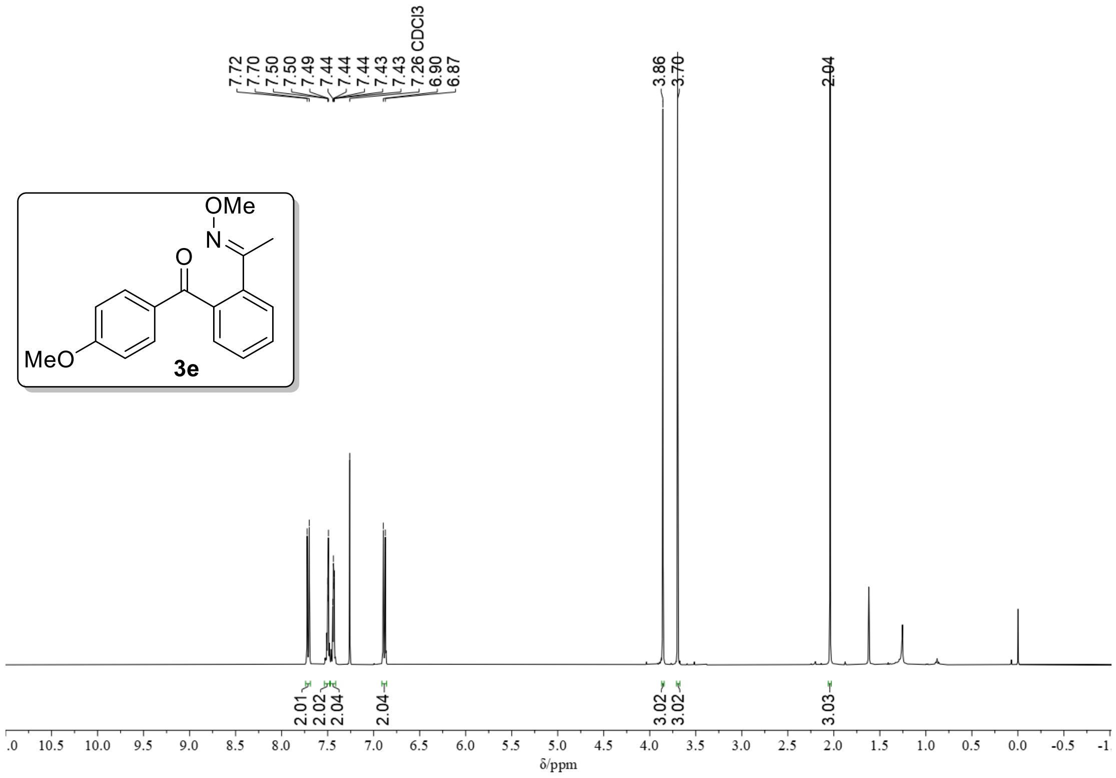

^{13}C NMR of compound **3e**

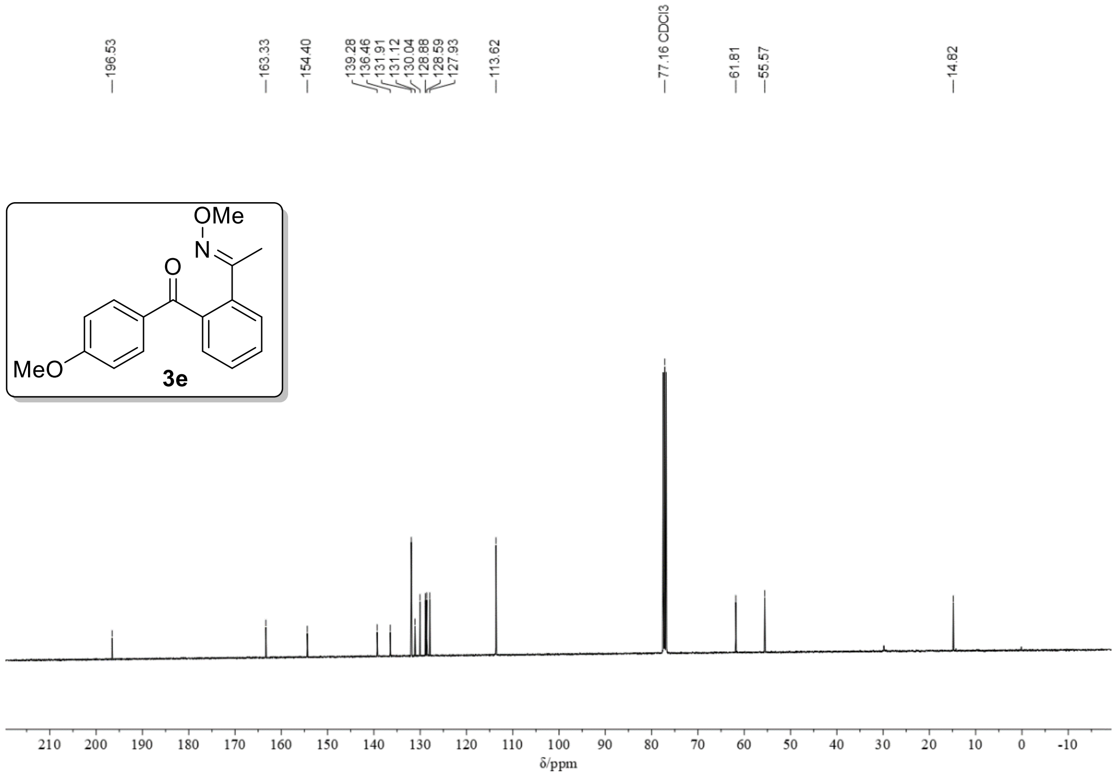

^1H NMR of compound **3f**

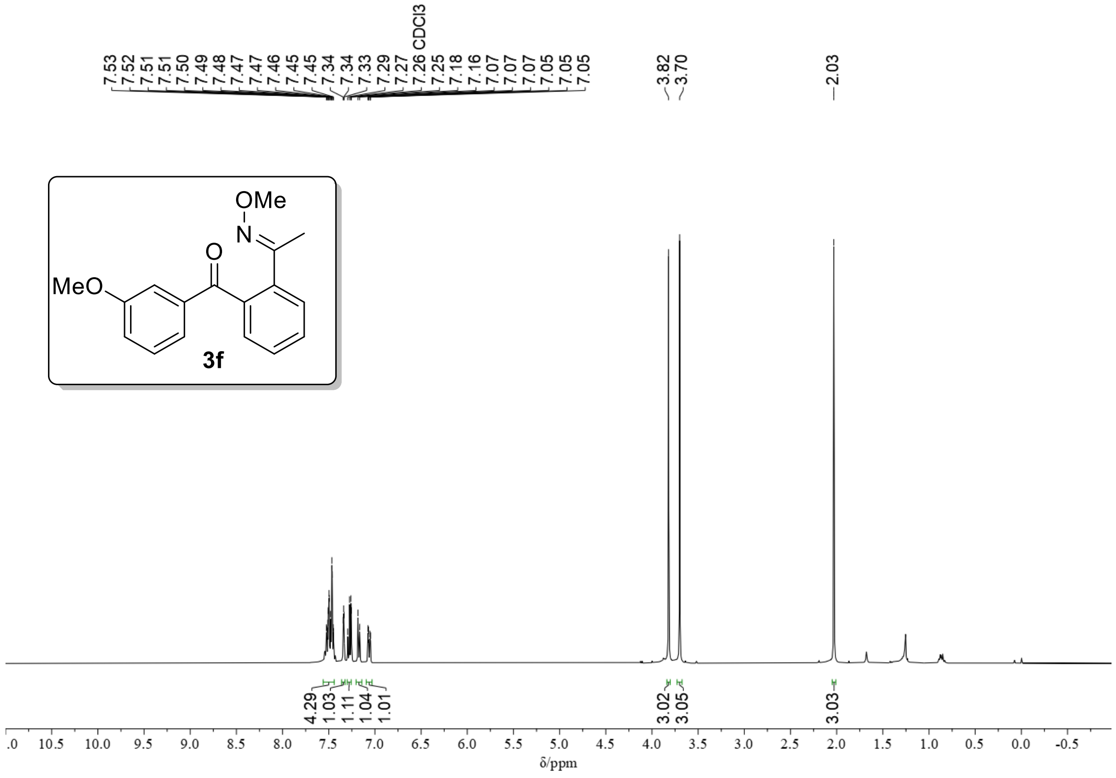

^{13}C NMR of compound **3f**

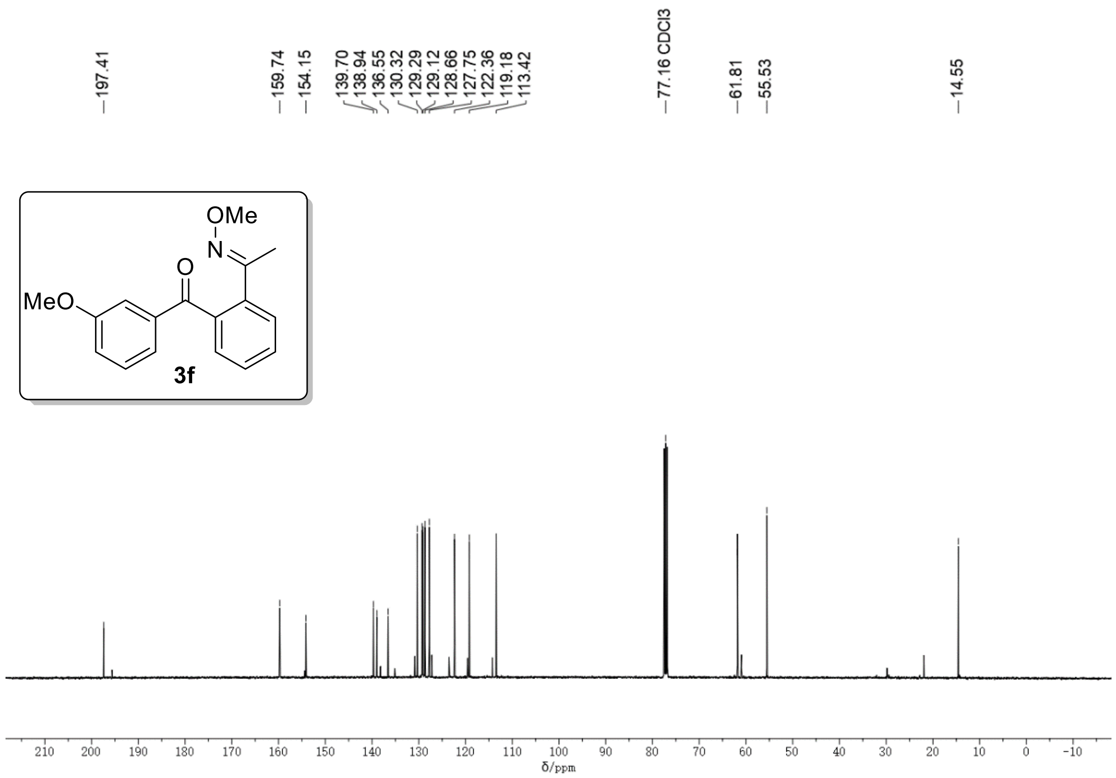

^{1}H NMR of compound **3g**

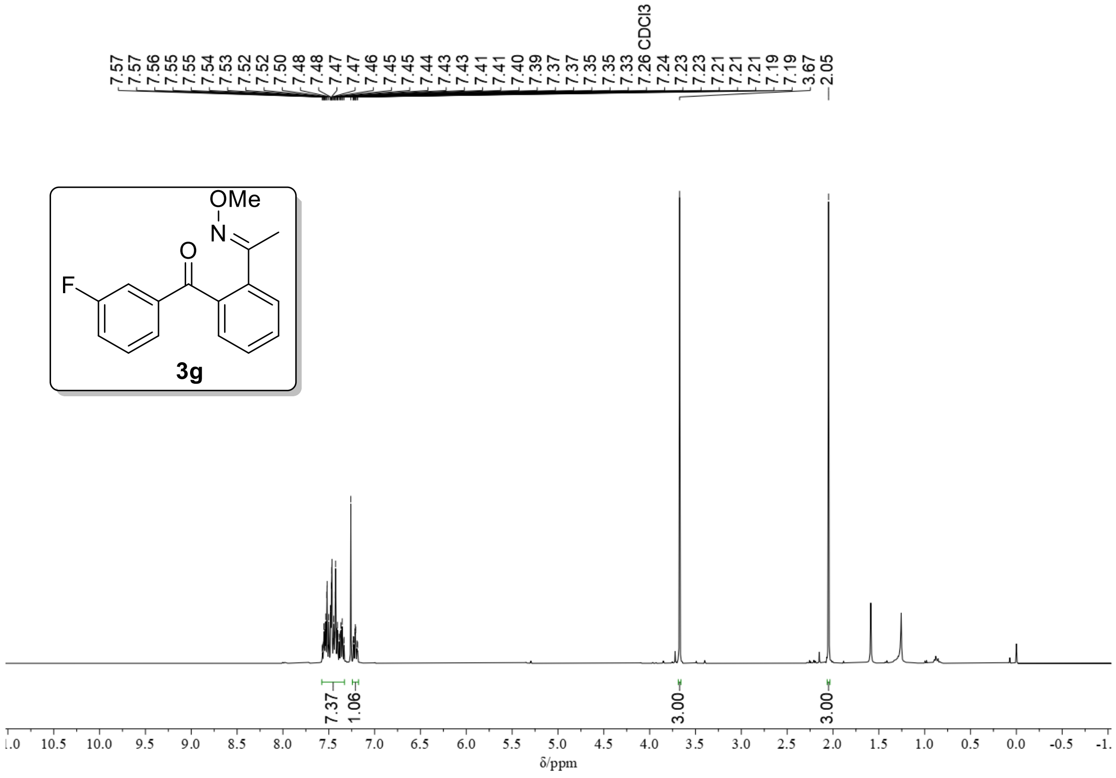

^{19}F NMR of compound **3g**

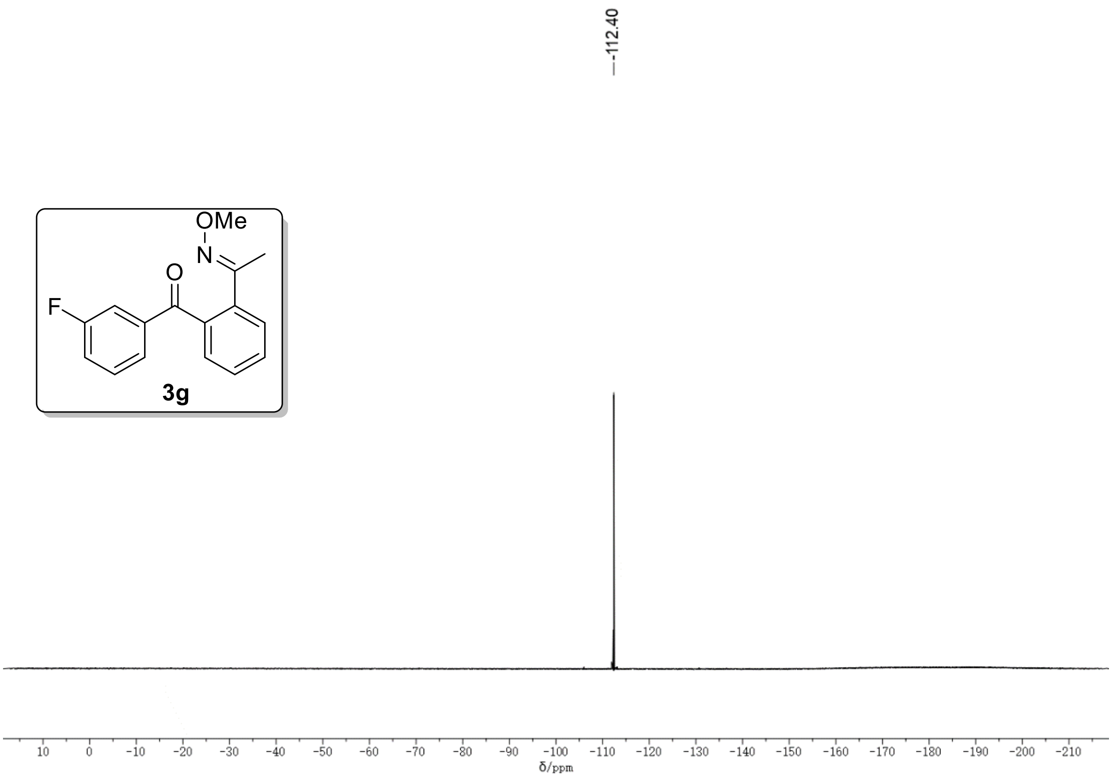

^{13}C NMR of compound **3g**

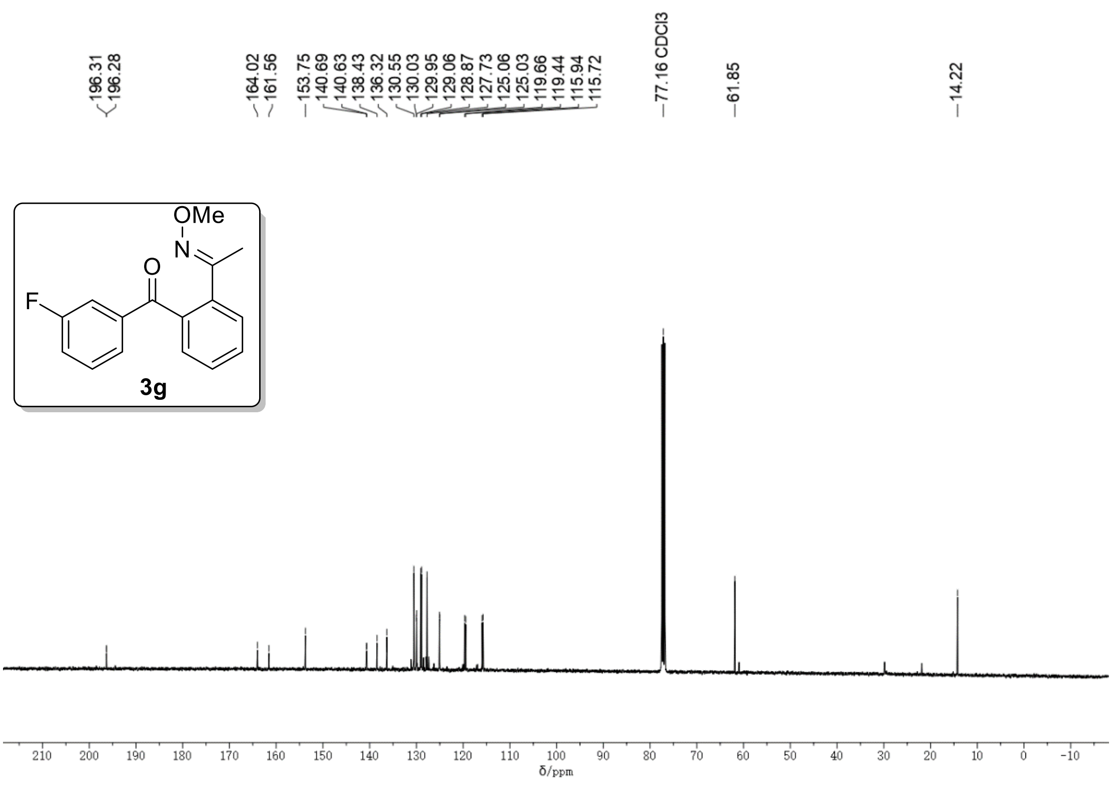

^{1}H NMR of compound **3h**

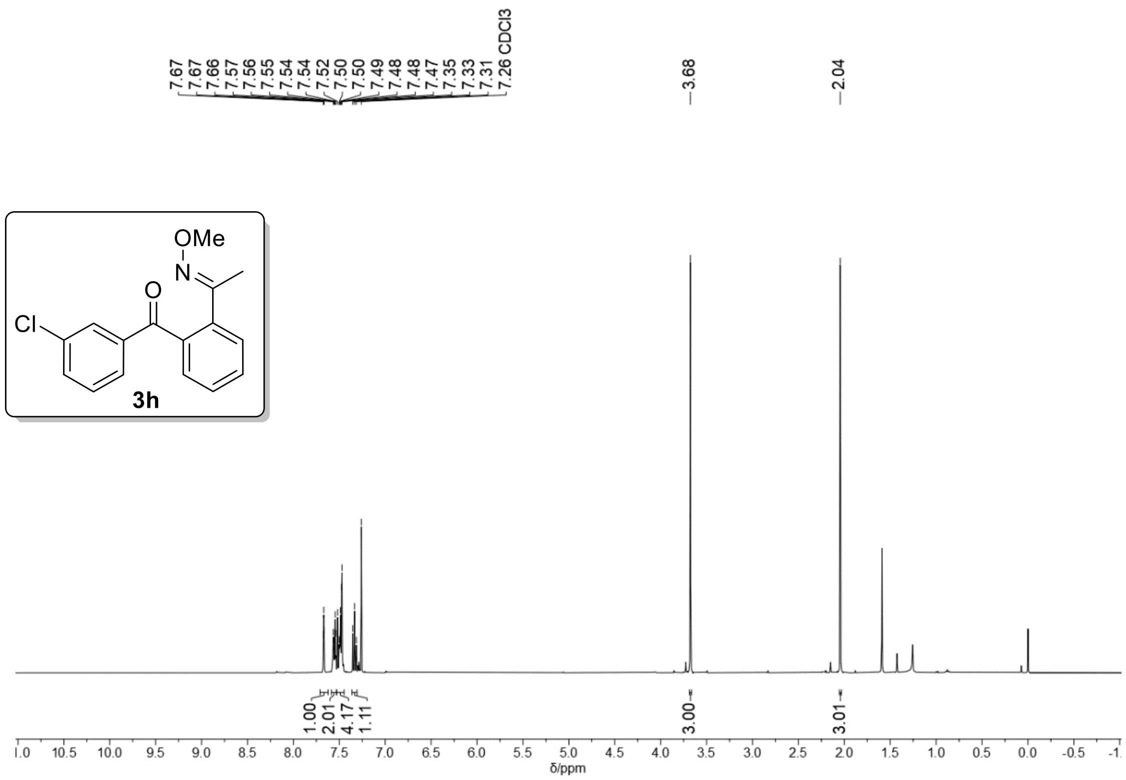

^{13}C NMR of compound **3h**

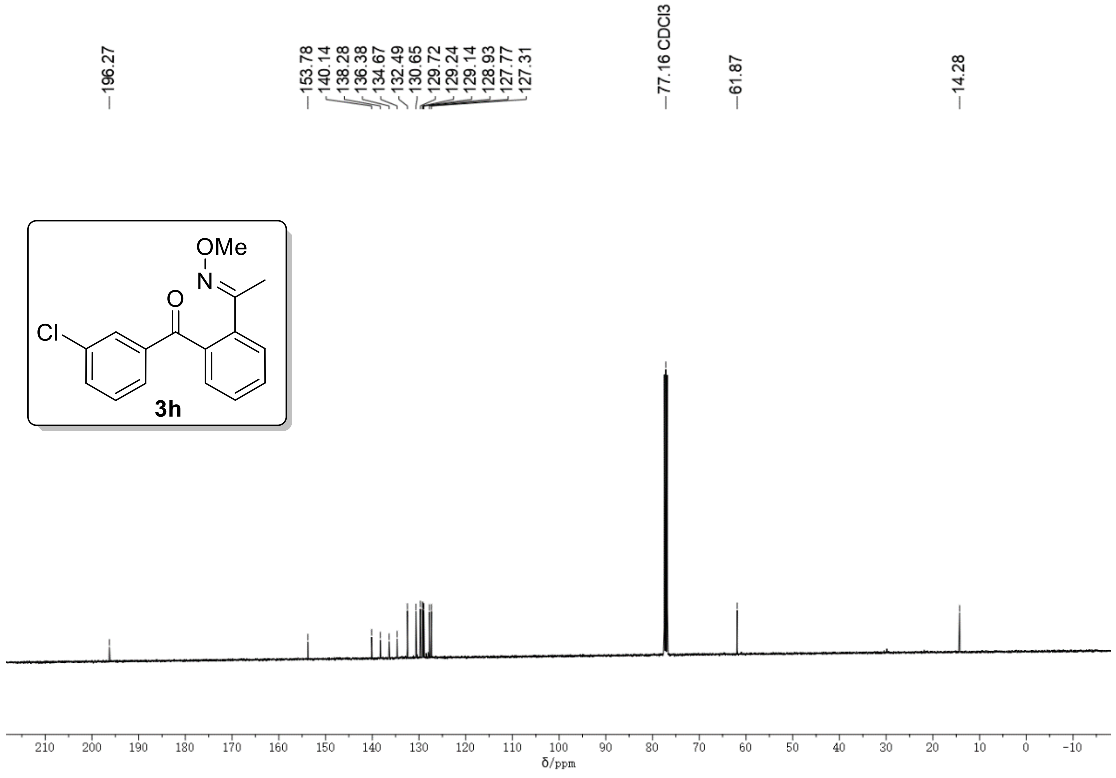

^{1}H NMR of compound **3i**

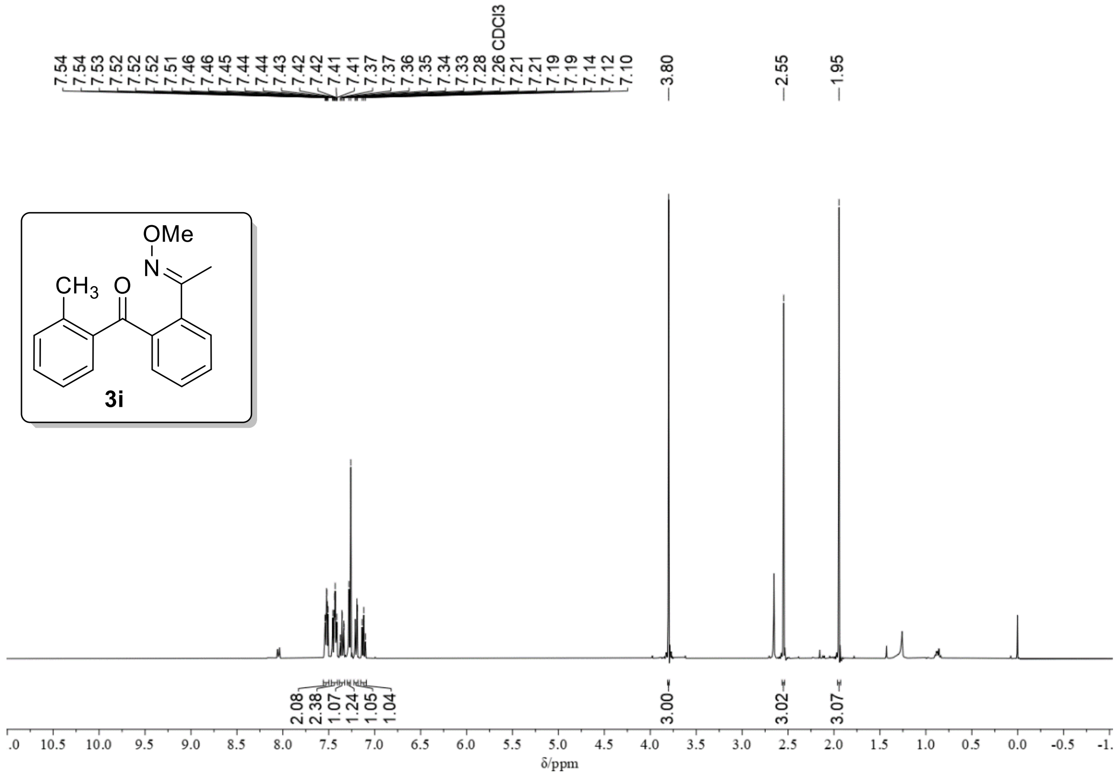

^{13}C NMR of compound **3i**

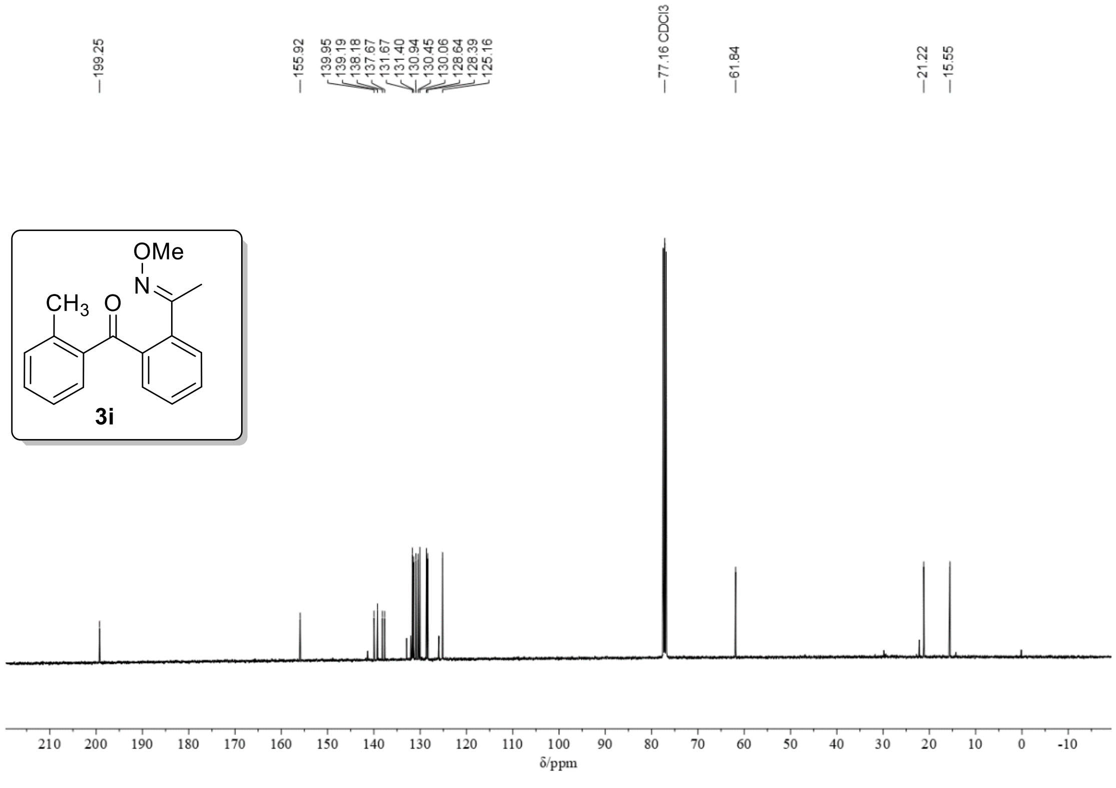

^{1}H NMR of compound **3j**

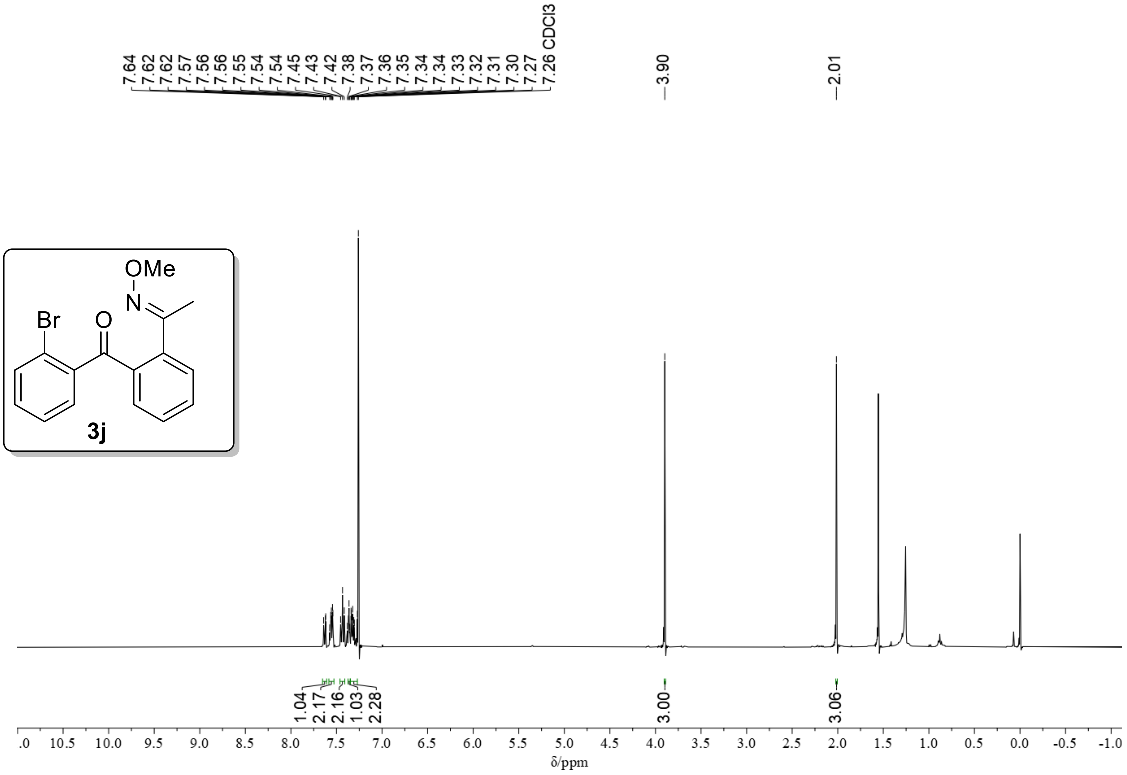

^{13}C NMR of compound **3j**

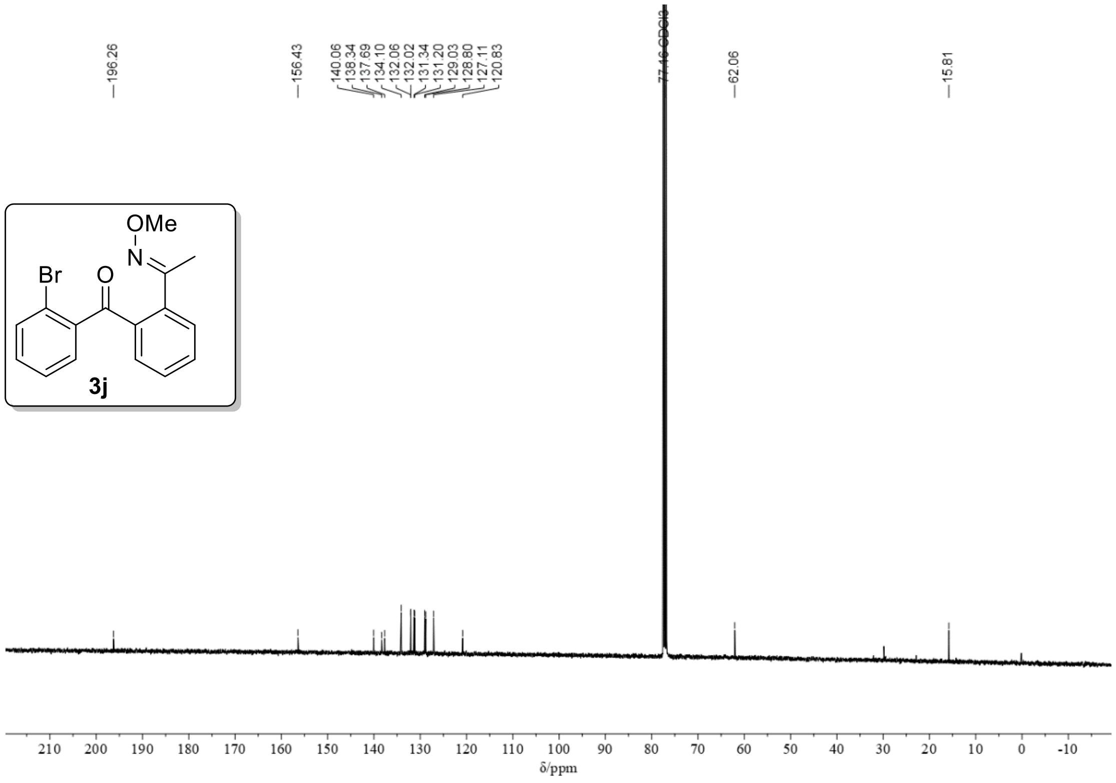

^{1}H NMR of compound **3k**

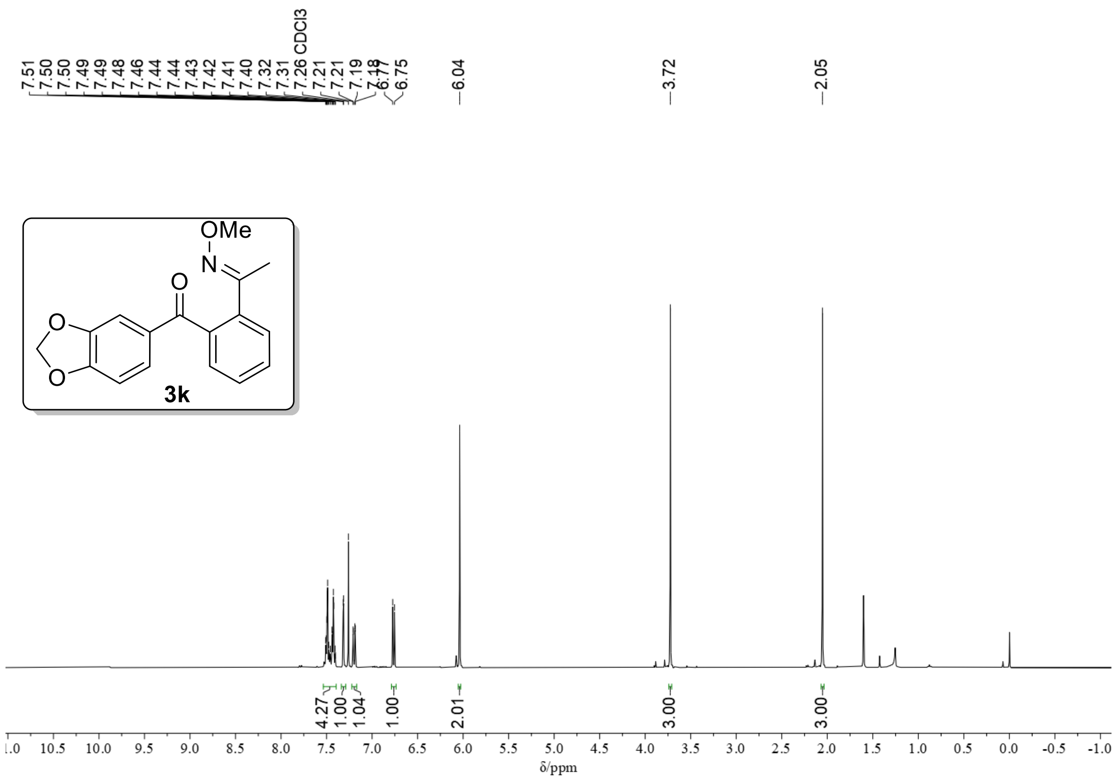

^{13}C NMR of compound **3k**

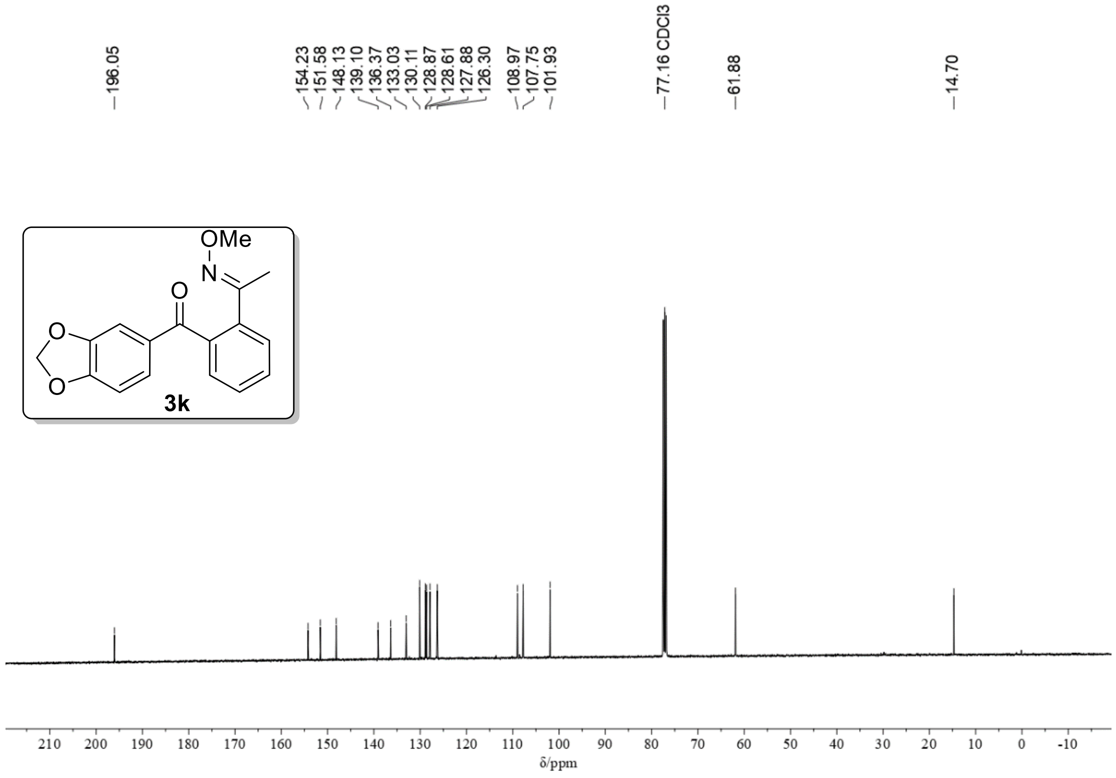

^{1}H NMR of compound **4a**

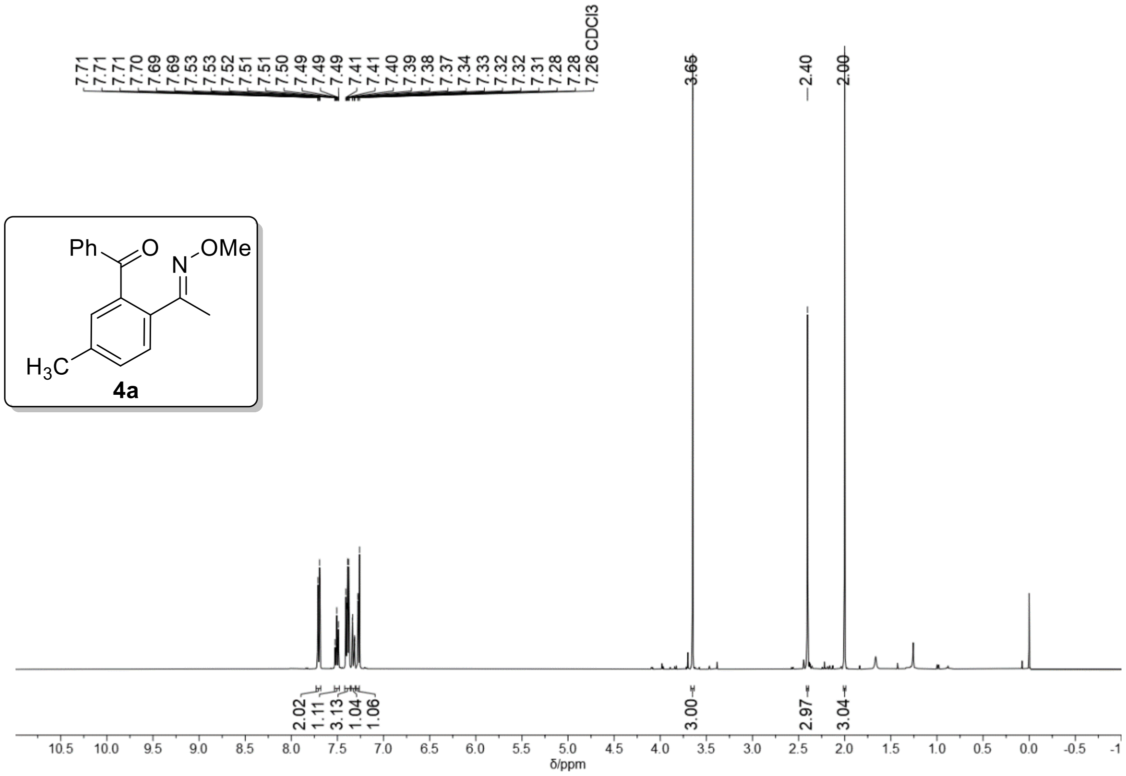

^{13}C NMR of compound **4a**

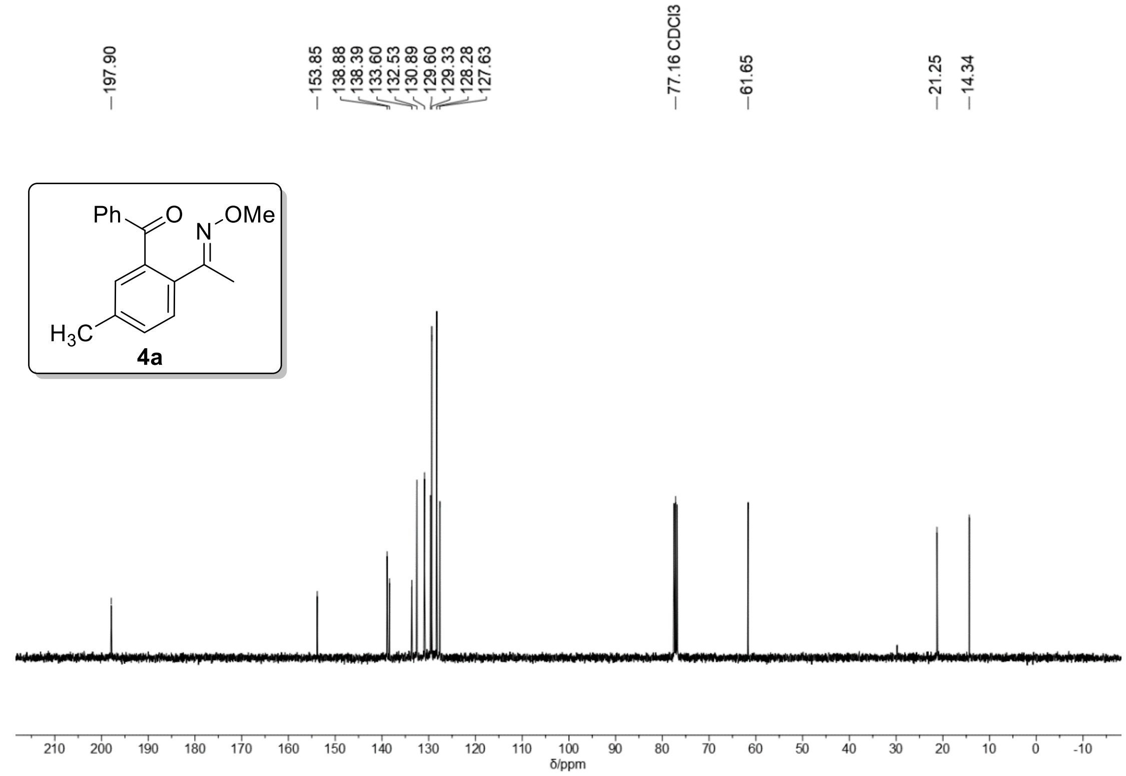

^{1}H NMR of compound **4b**

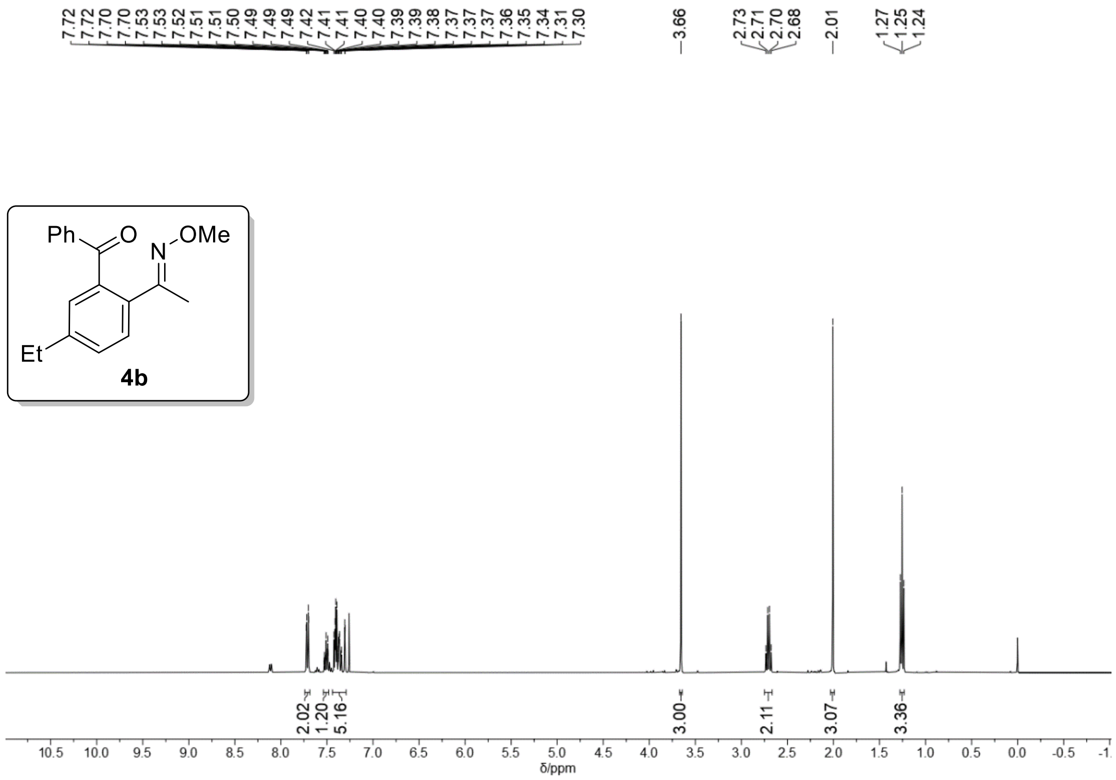

^{13}C NMR of compound **4b**

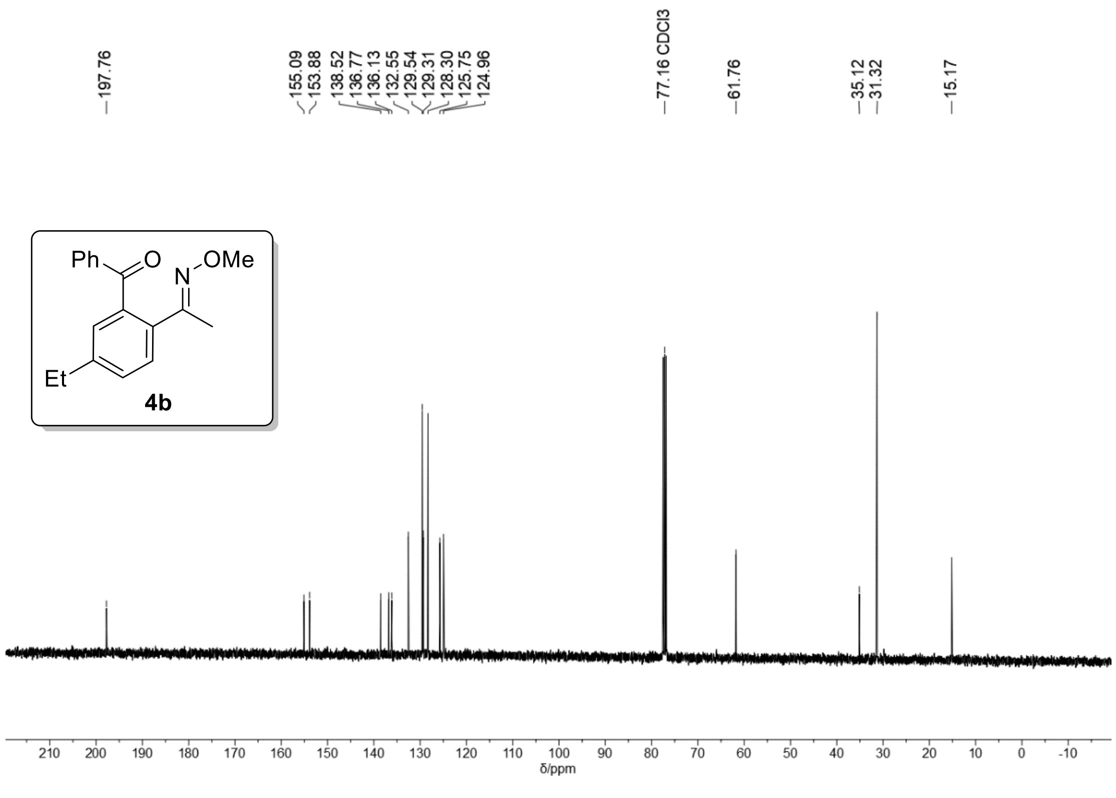

^{1}H NMR of compound **4c**

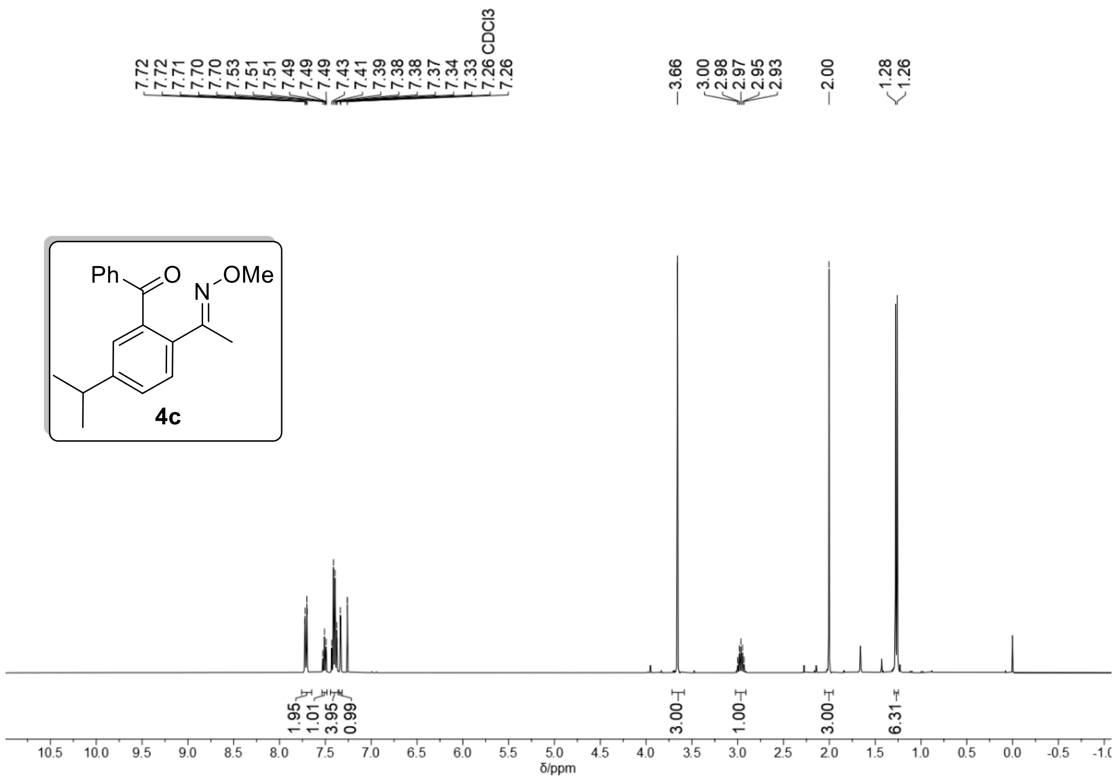

^{13}C NMR of compound **4c**

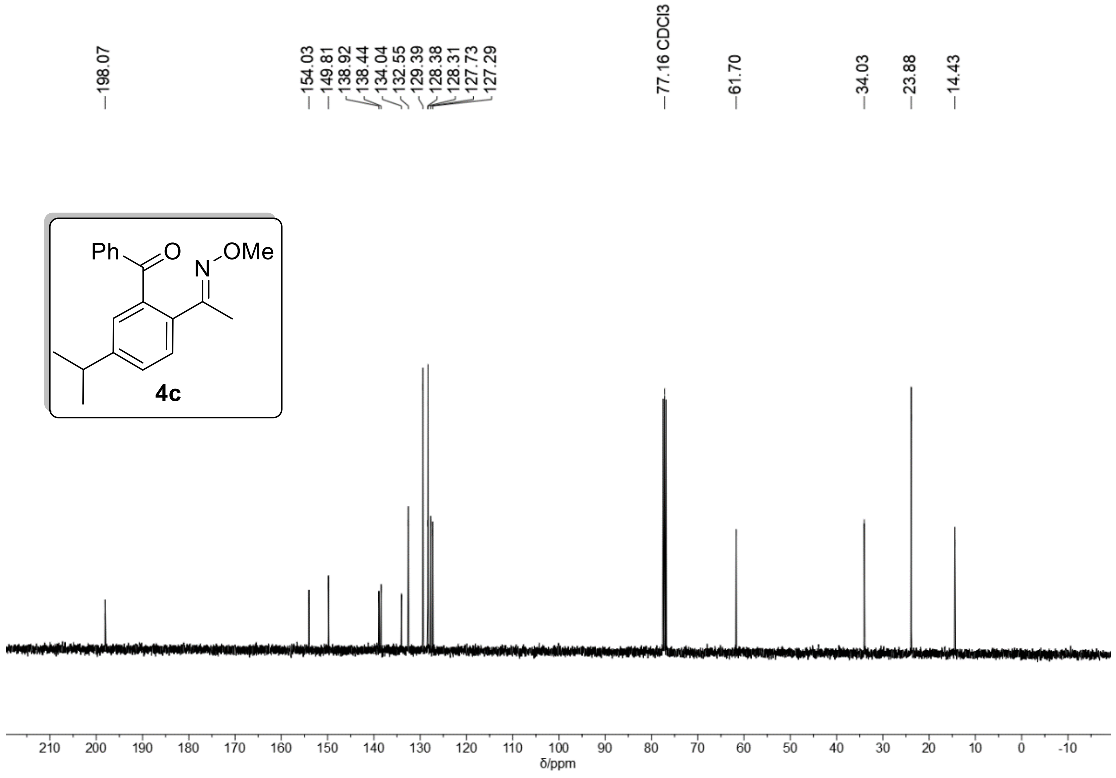

^{1}H NMR of compound **4d**

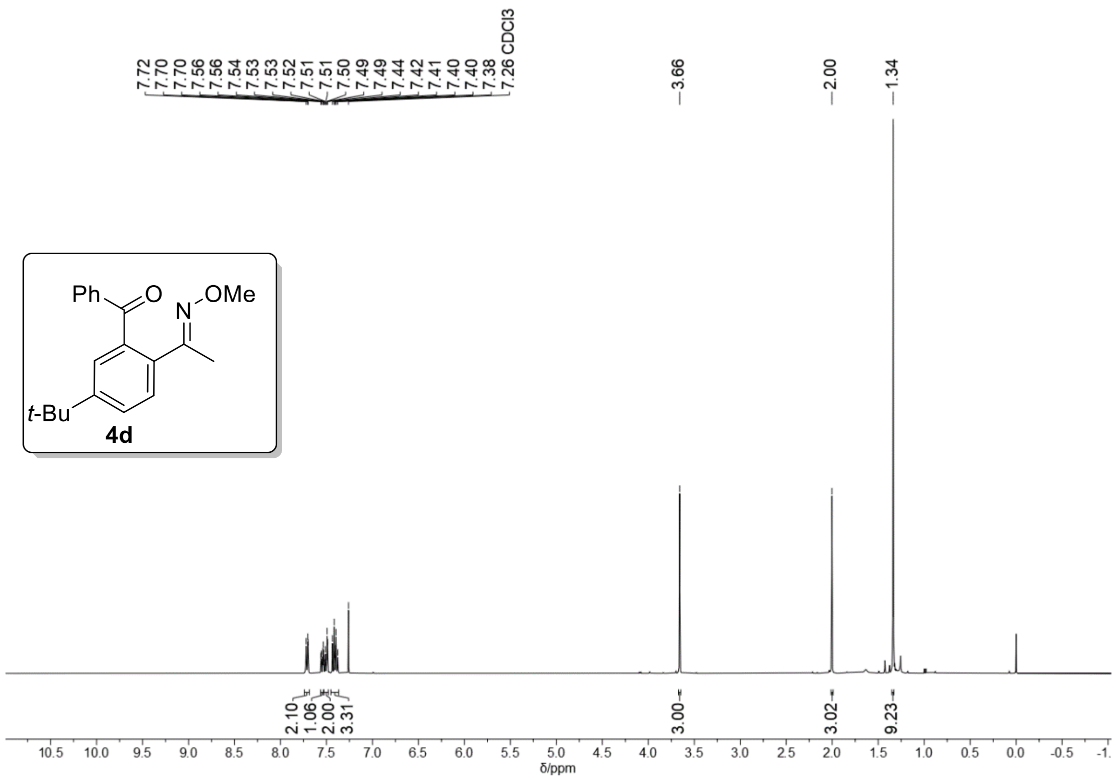

^{13}C NMR of compound **4d**

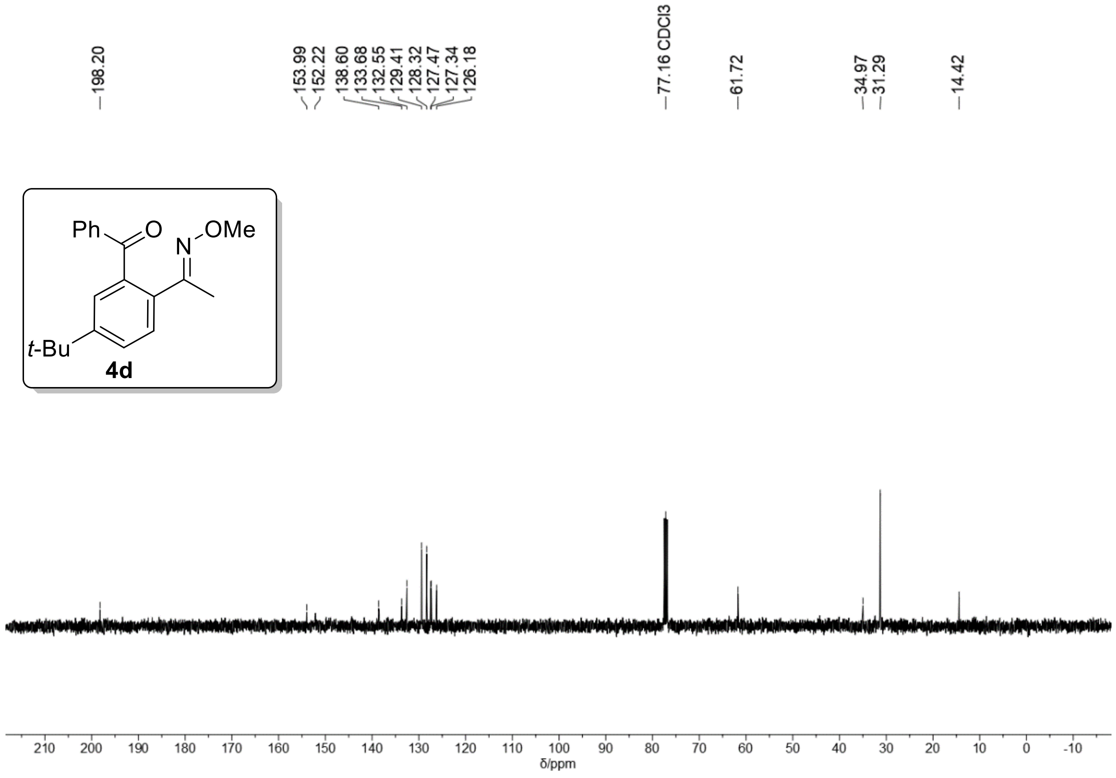

^{1}H NMR of compound **4e**

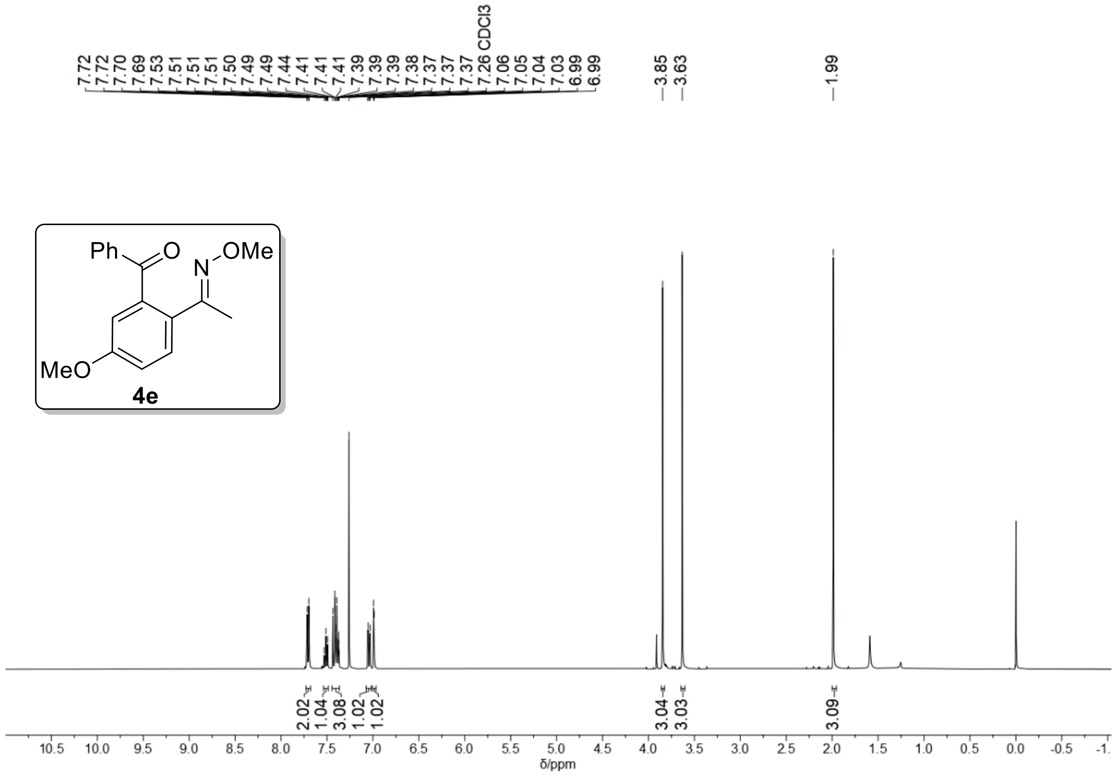

^{13}C NMR of compound **4e**

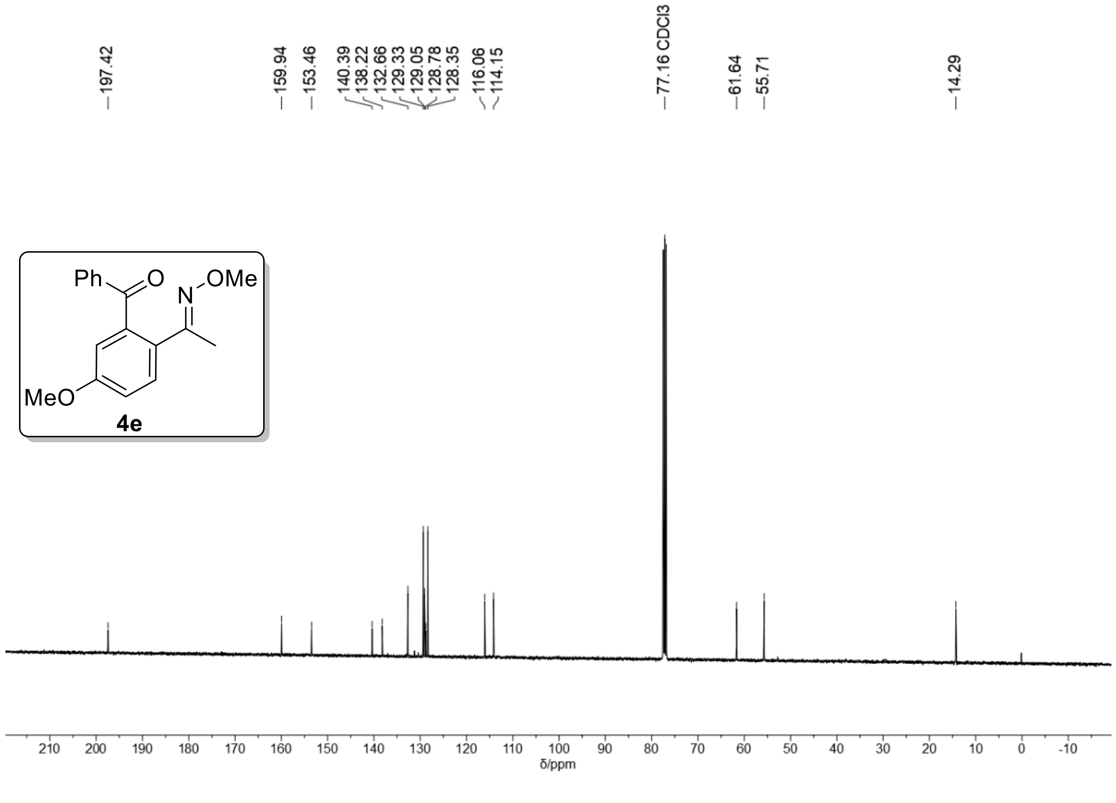

^{1}H NMR of compound **4f**

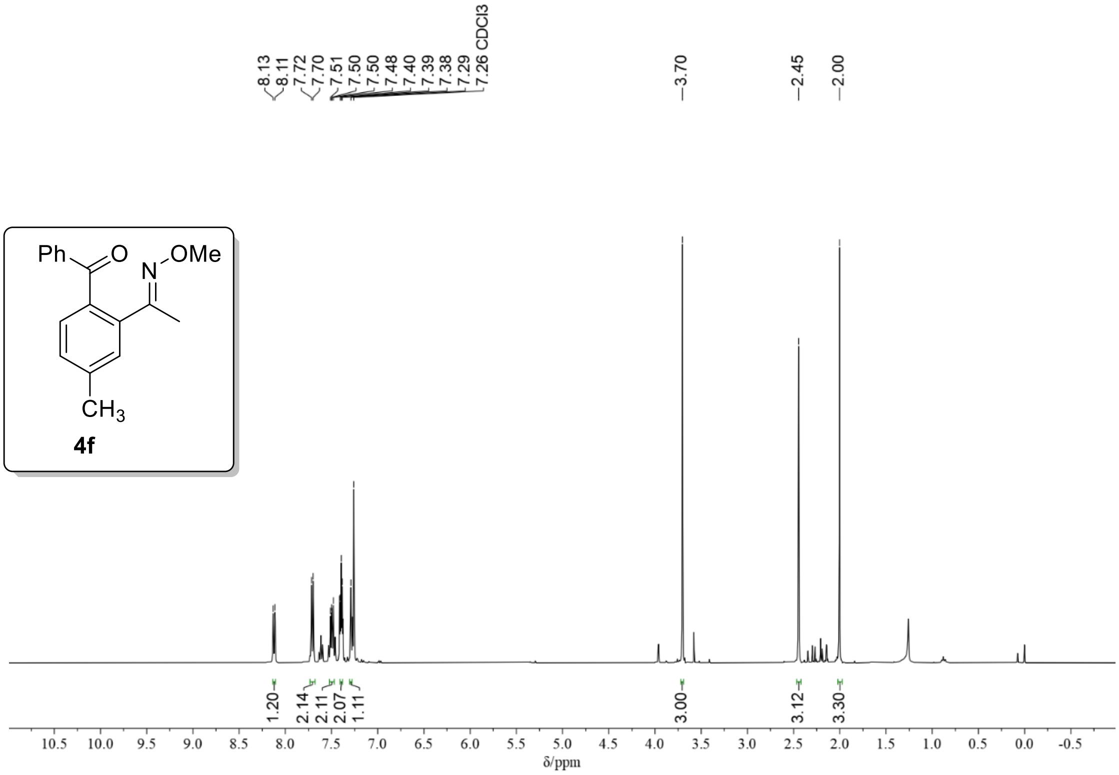

^{13}C NMR of compound **4f**

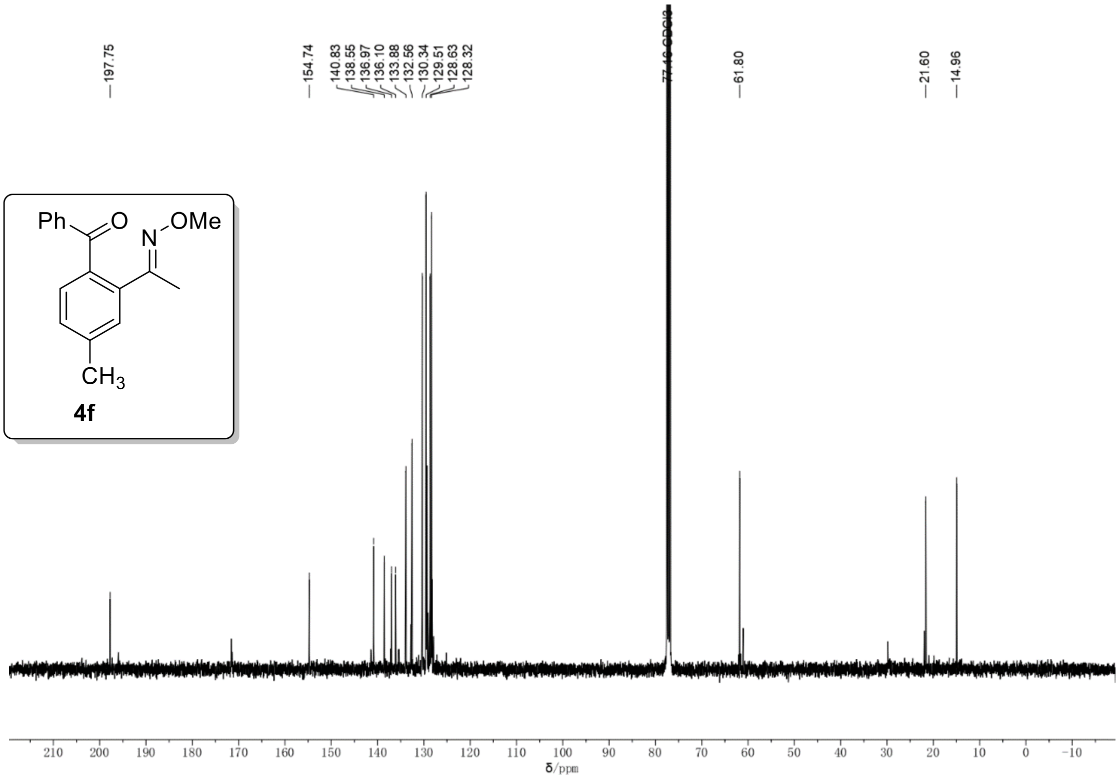

^{1}H NMR of compound **4g**

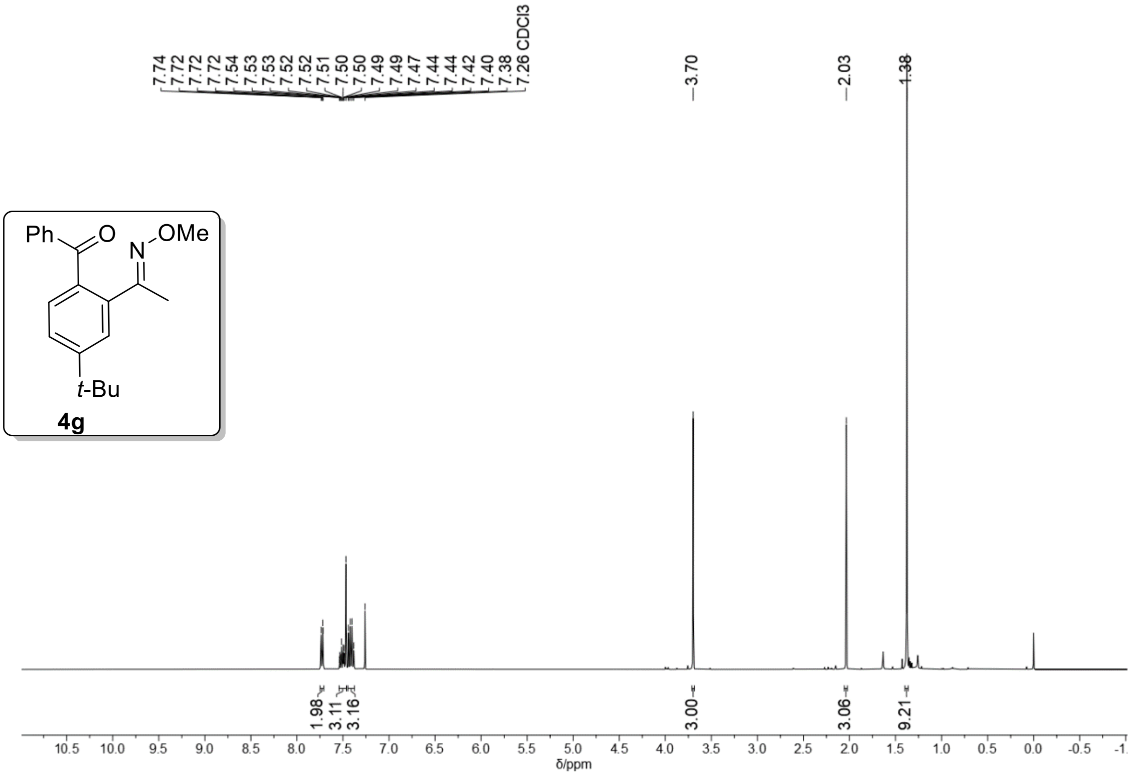

^{13}C NMR of compound **4g**

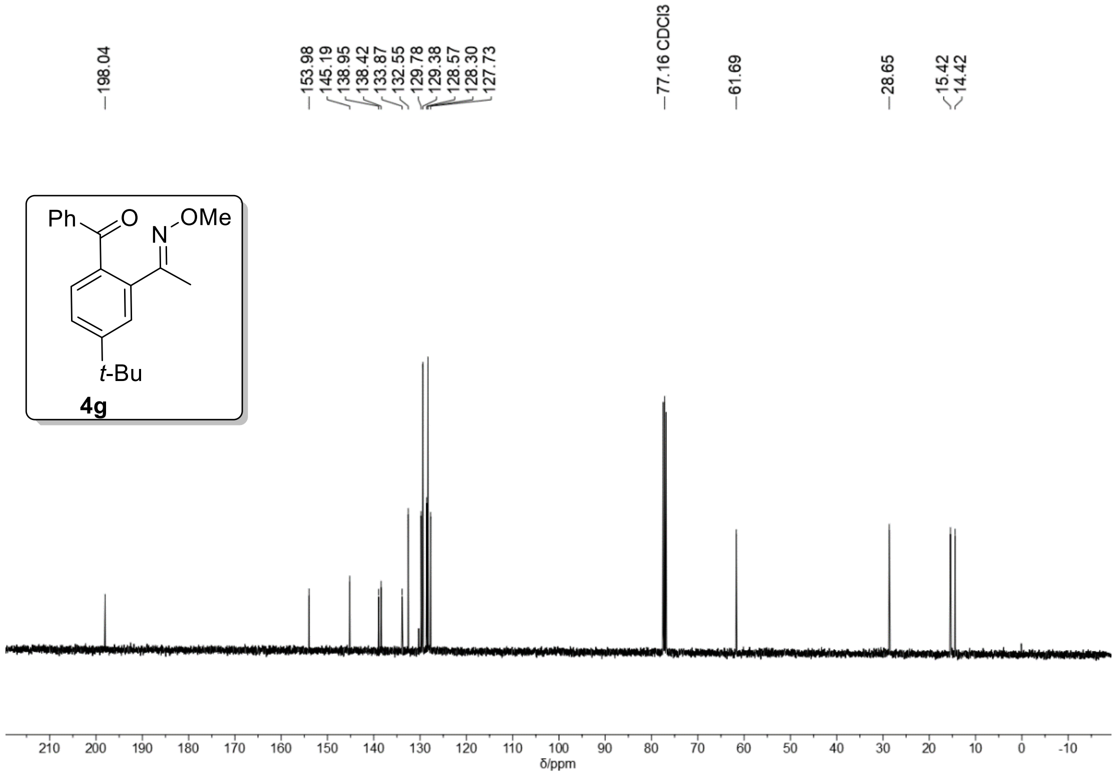

^{1}H NMR of compound **4h**

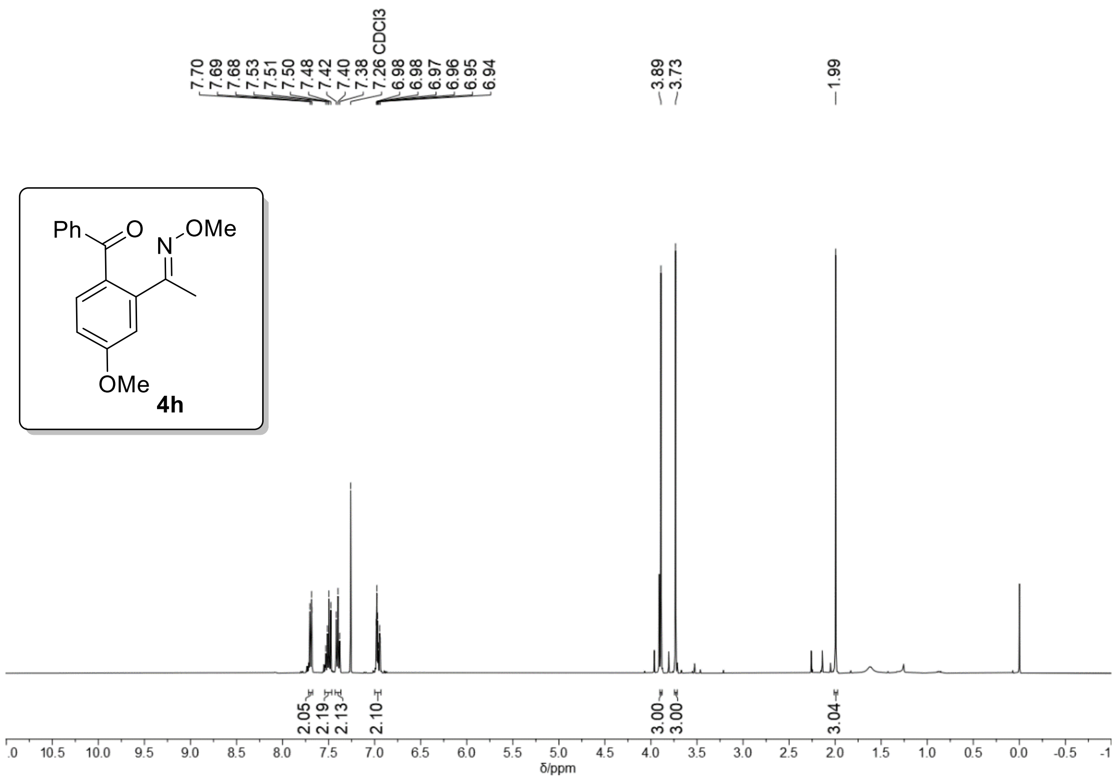

^{13}C NMR of compound **4h**

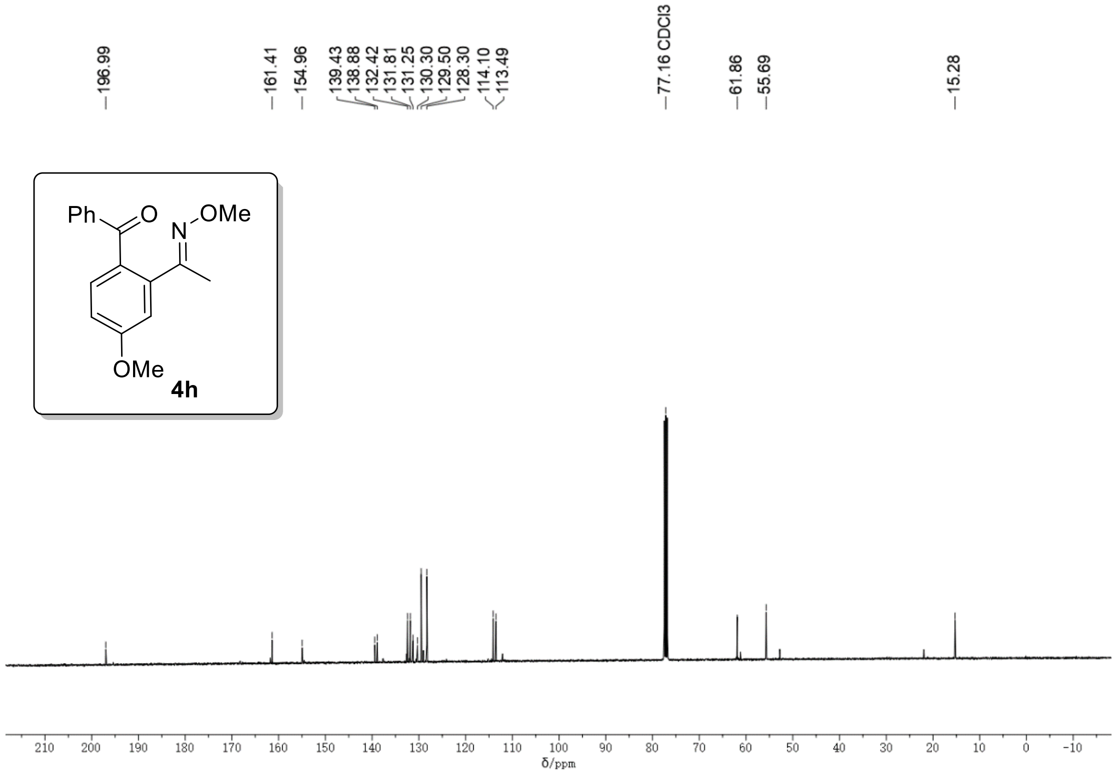

^{1}H NMR of compound **4i**

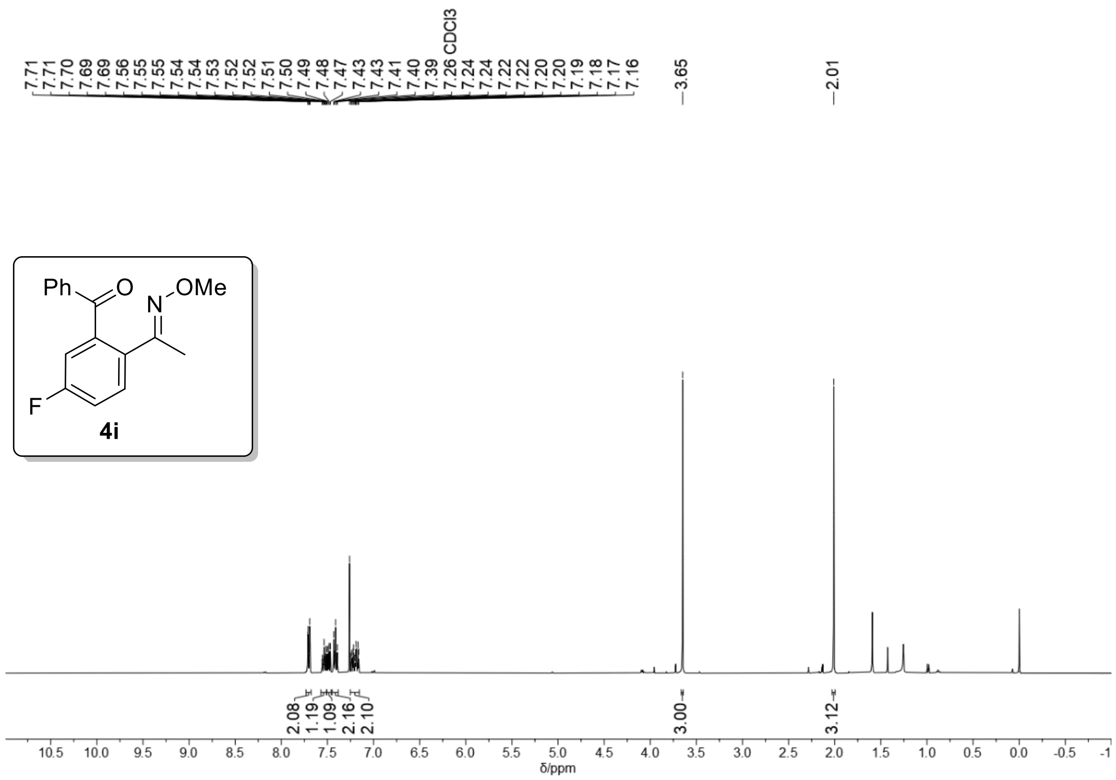

^{19}F NMR of compound **4i**

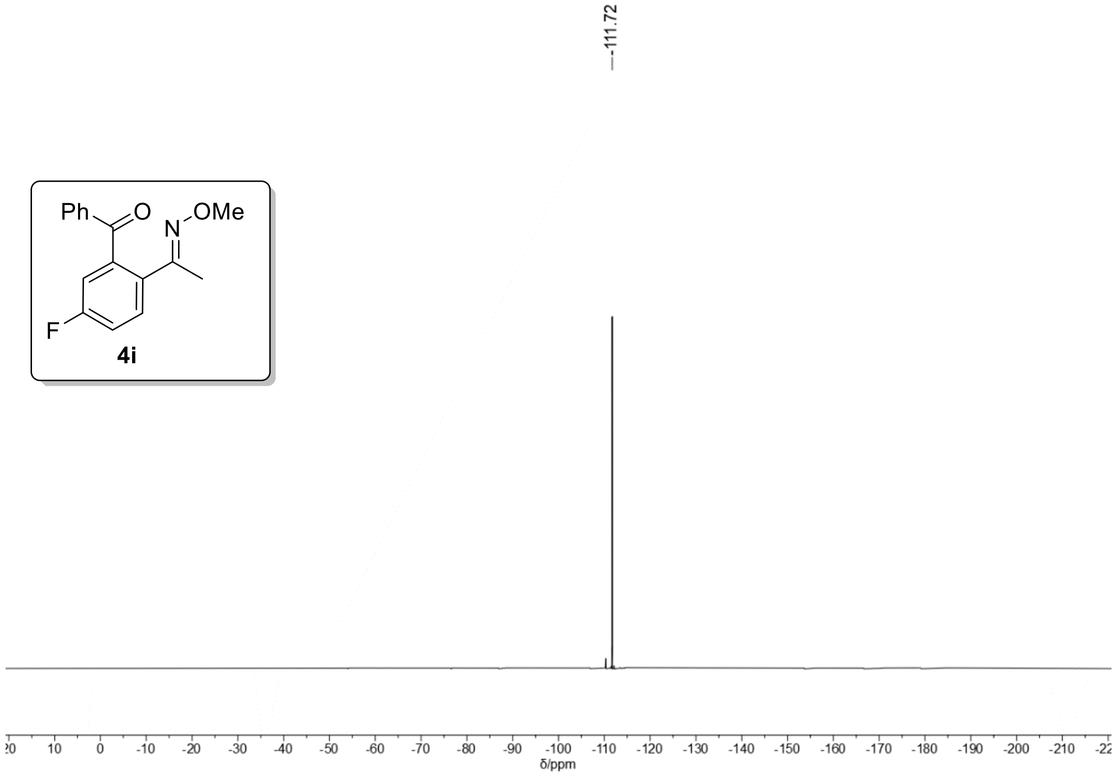

^{13}C NMR of compound **4i**

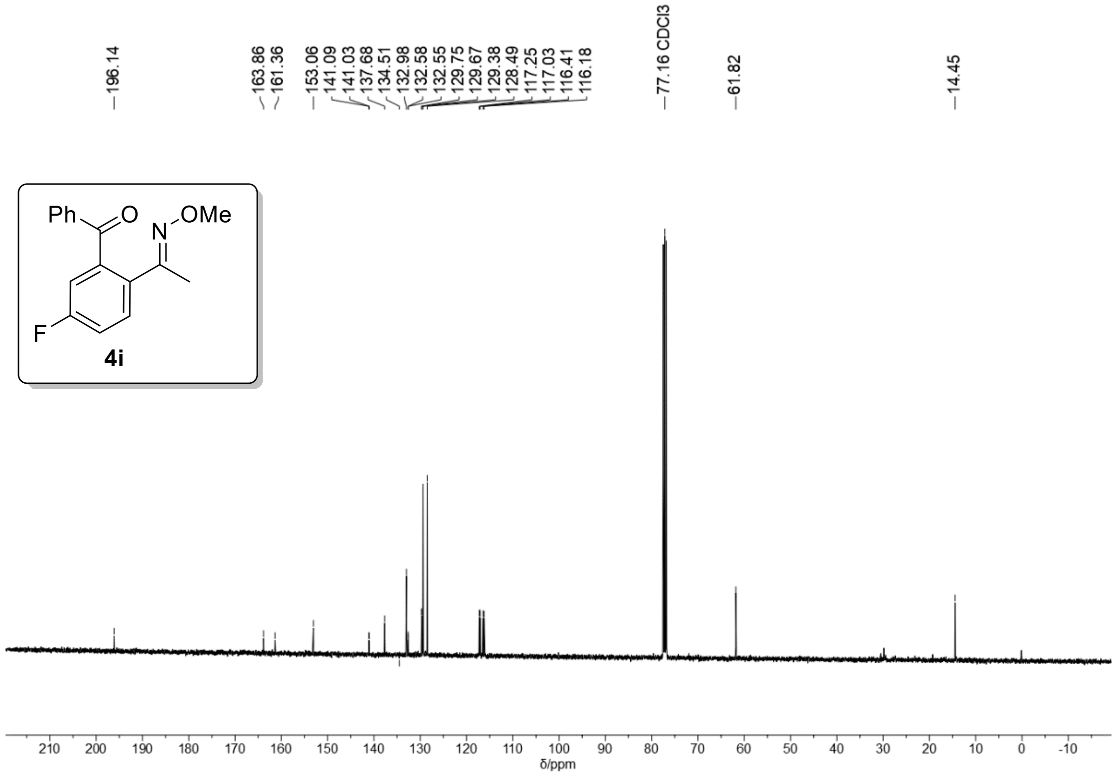

^{1}H NMR of compound **4j**

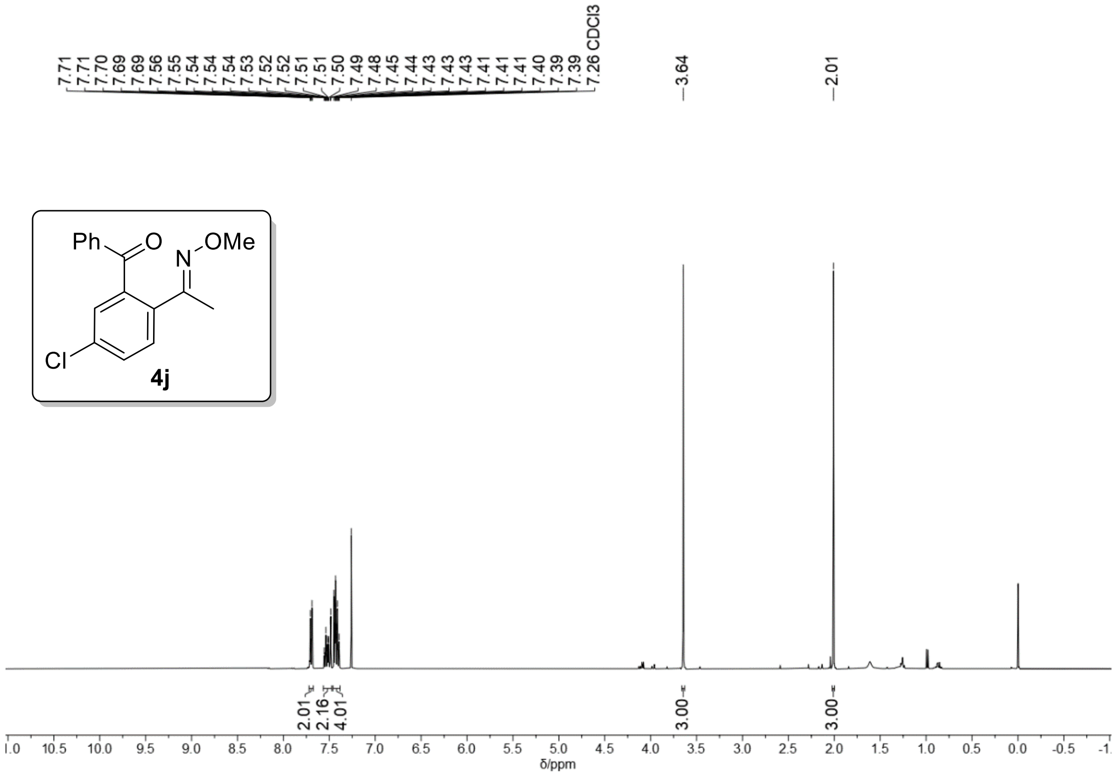

^{13}C NMR of compound **4j**

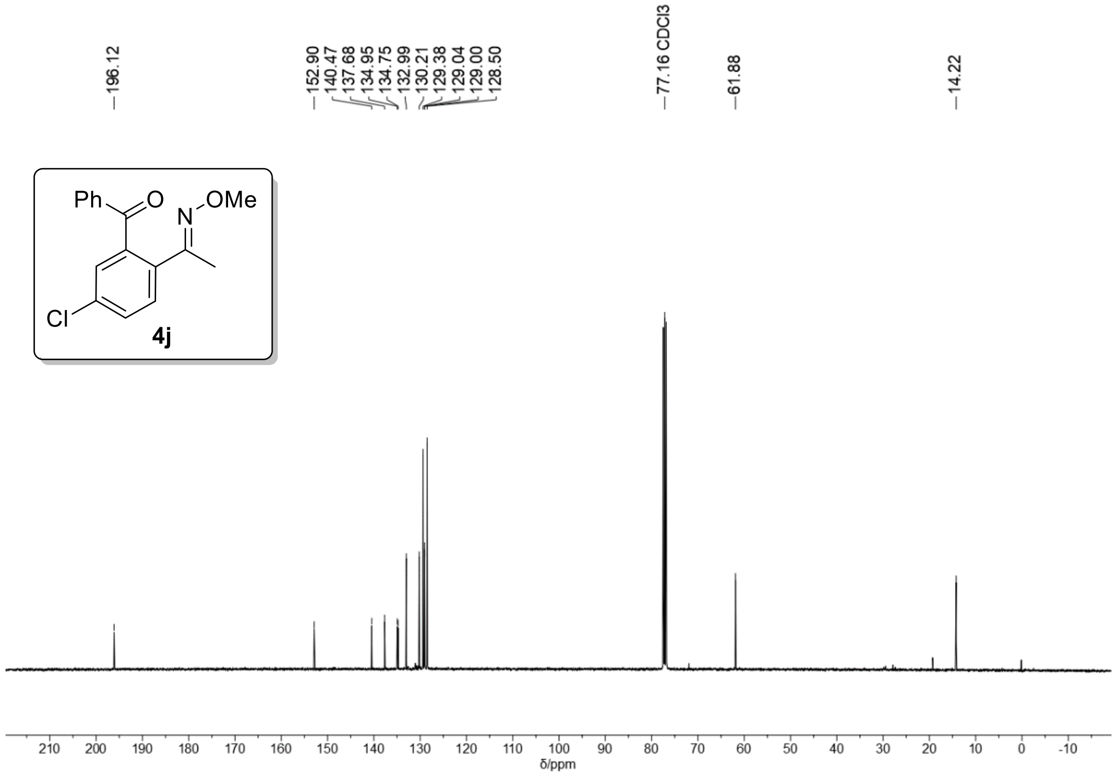

^{1}H NMR of compound **4k**

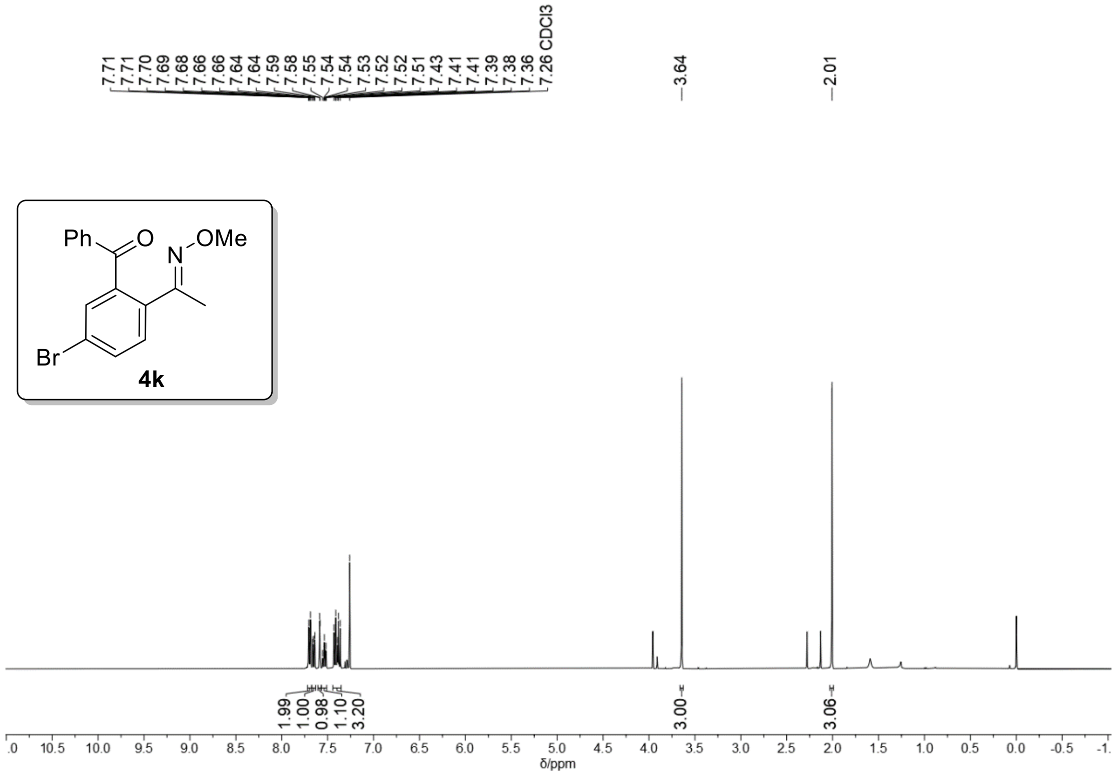

^{13}C NMR of compound **4k**

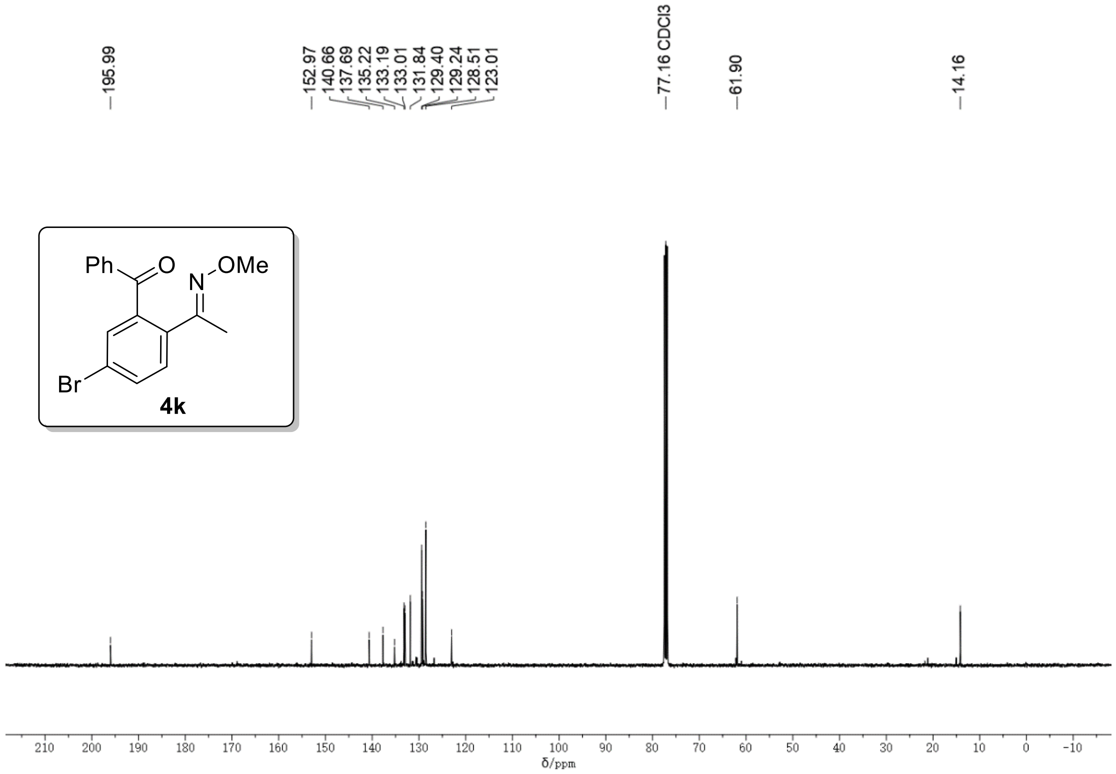

^{1}H NMR of compound **4l**

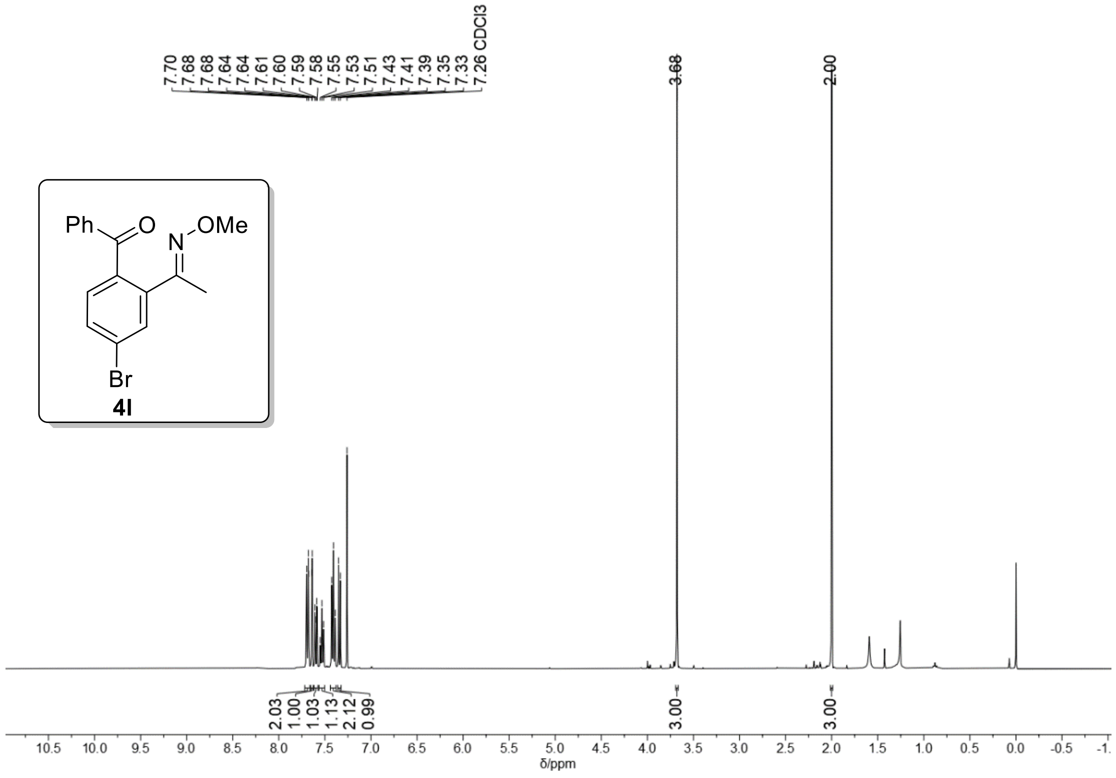

^{13}C NMR of compound **4l**

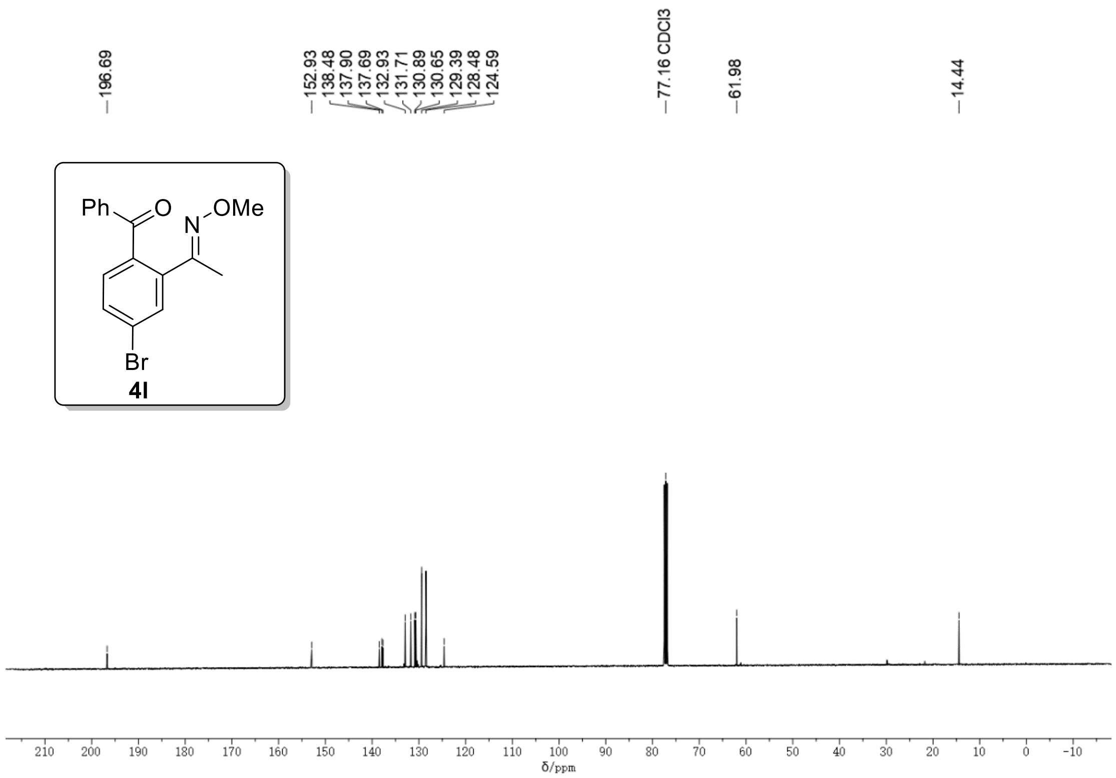

^{1}H NMR of compound **4m**

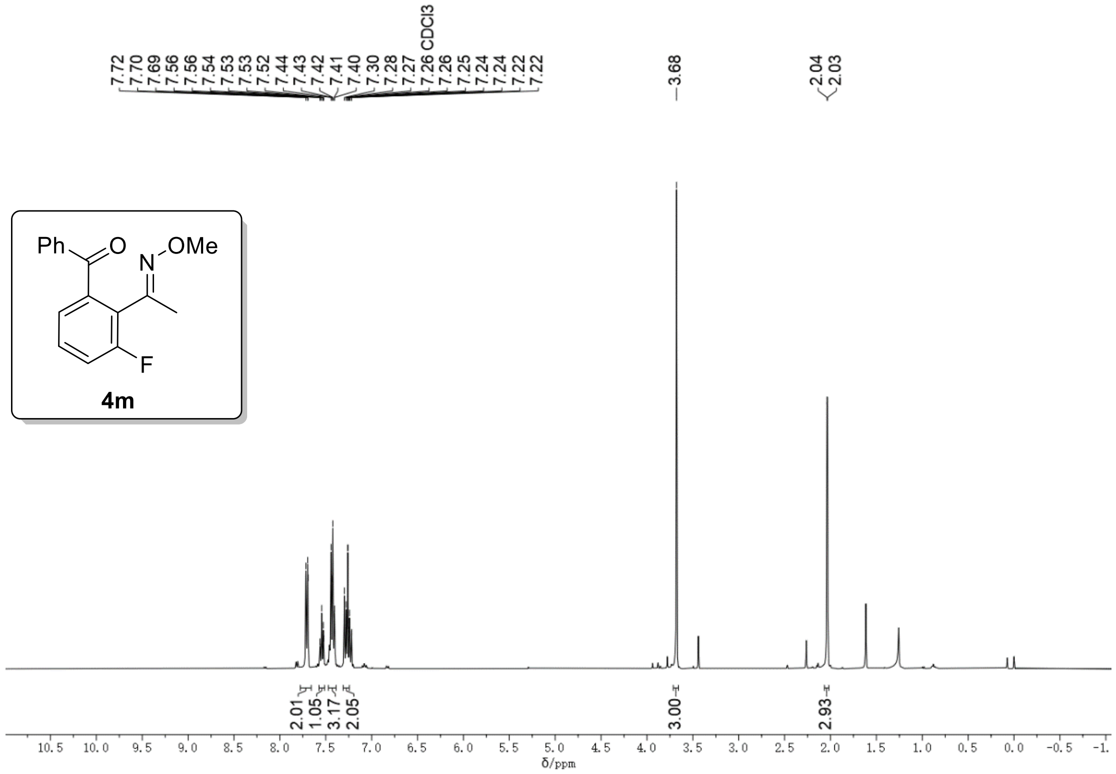

^{19}F NMR of compound **4m**

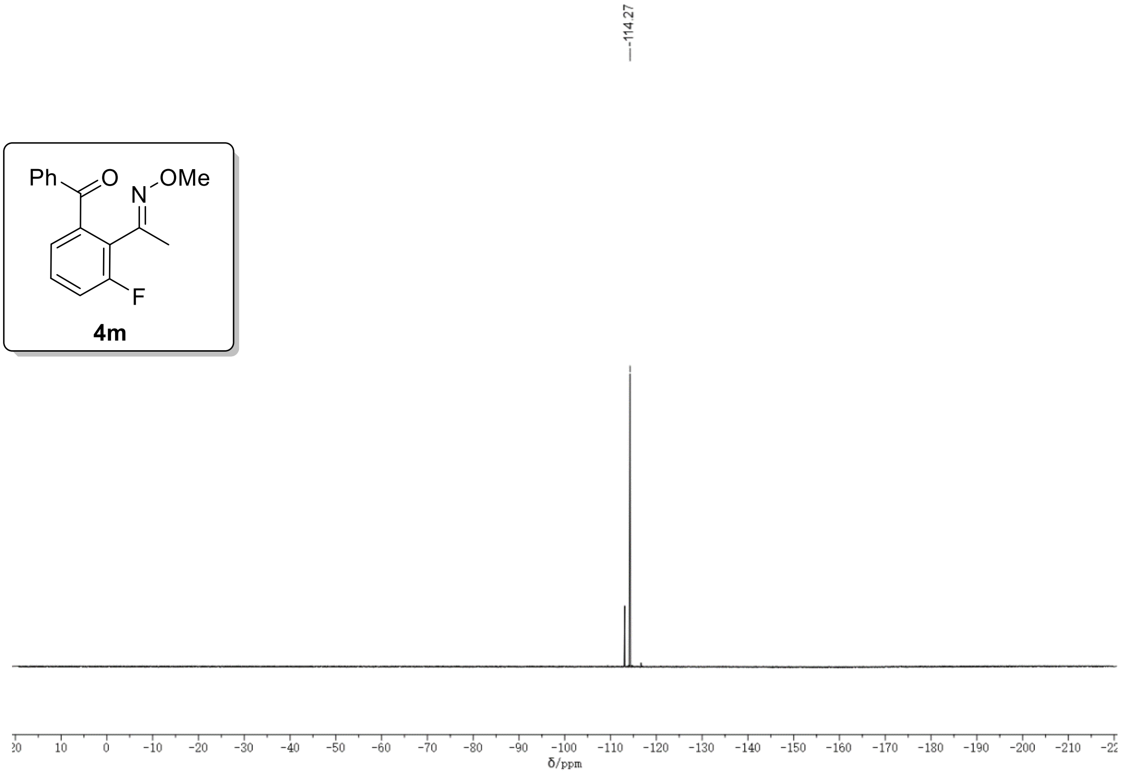

^{13}C NMR of compound **4m**

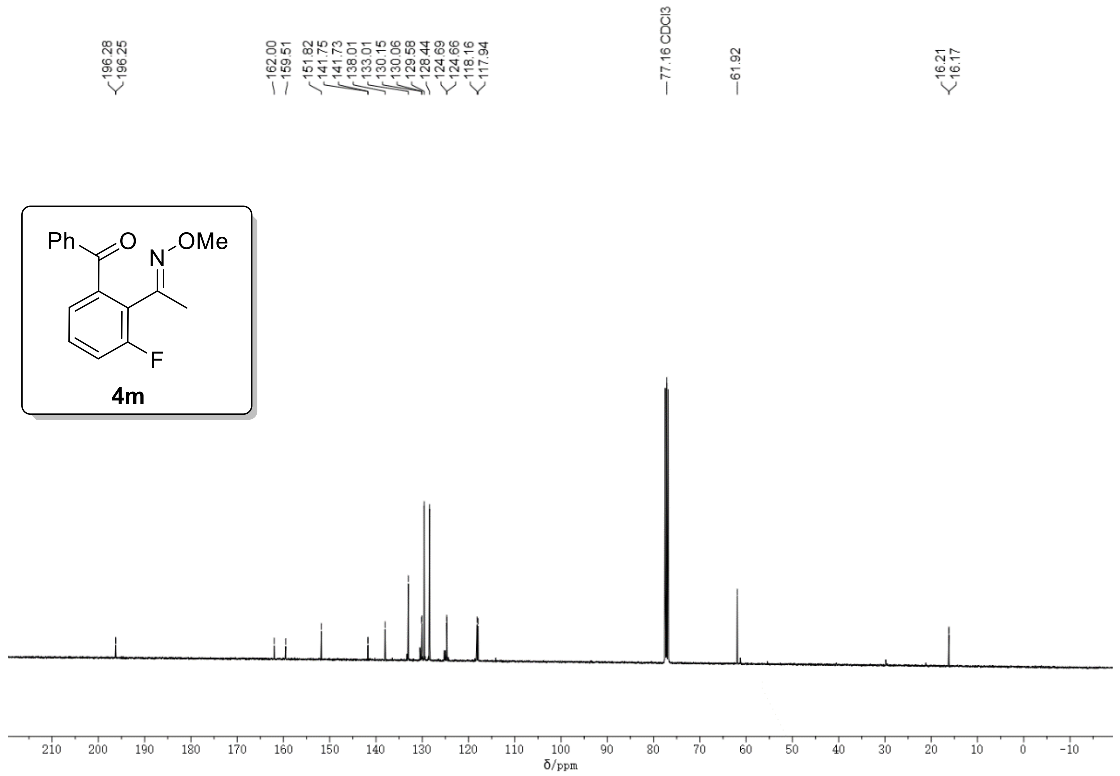

^{1}H NMR of compound **4n**

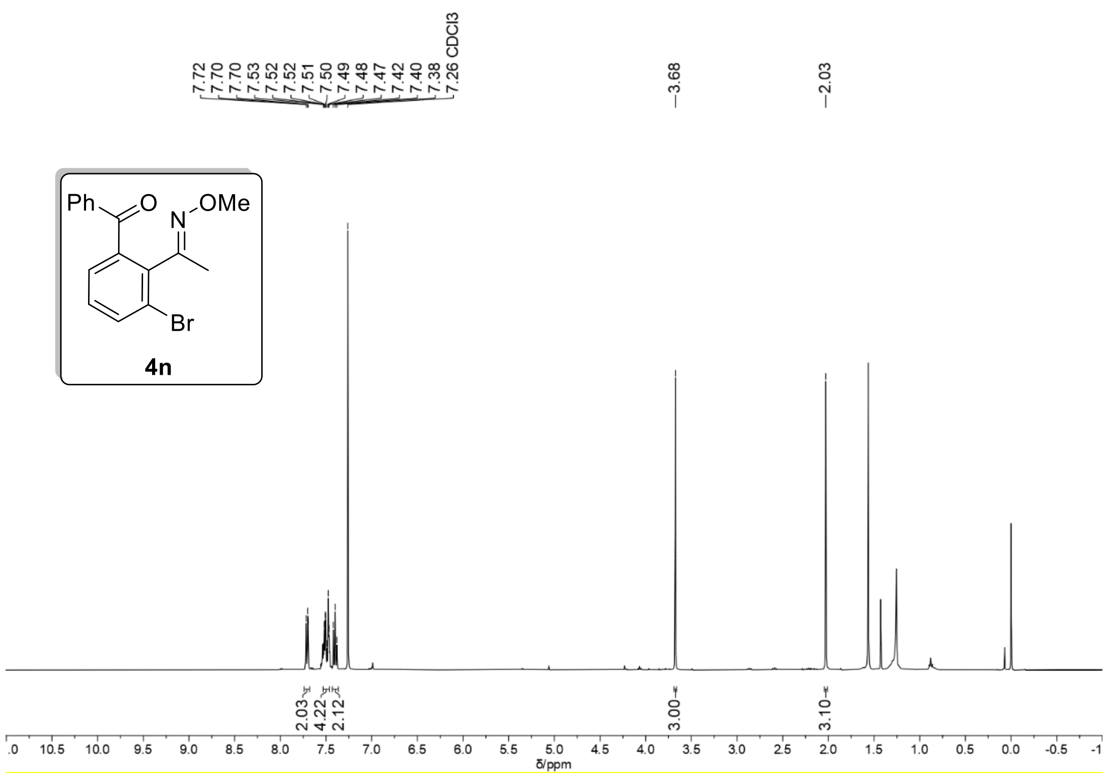

^{13}C NMR of compound **4n**

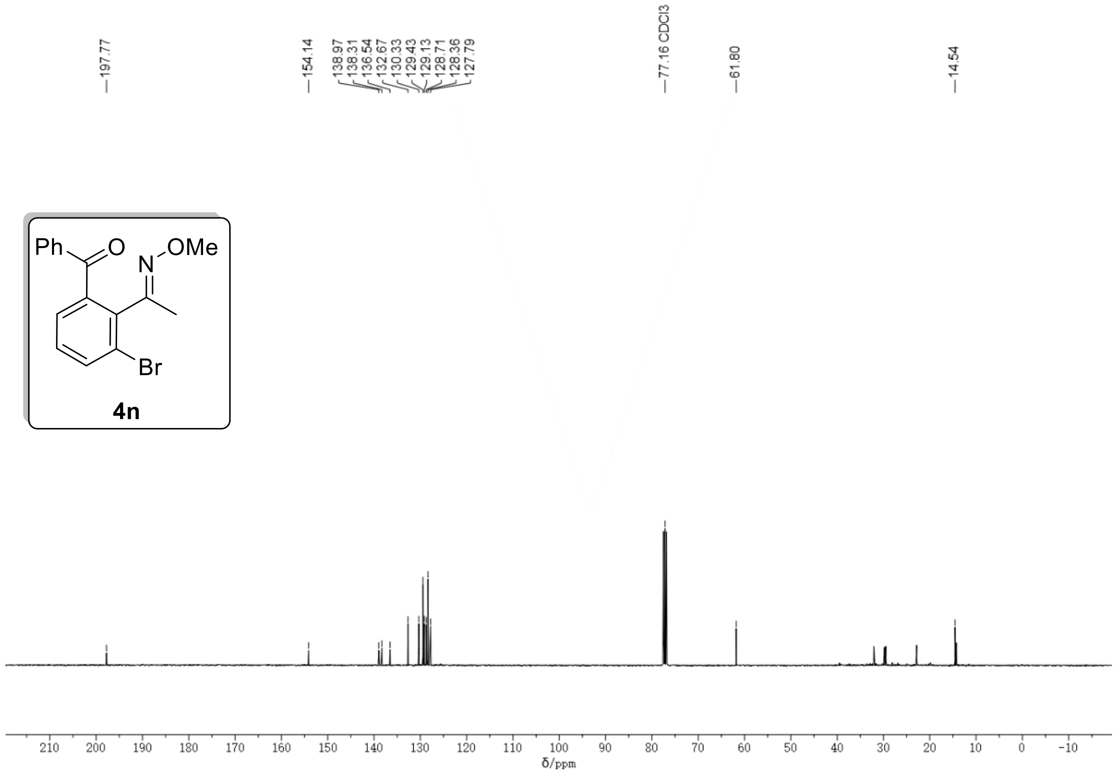

^{1}H NMR of compound **4o**

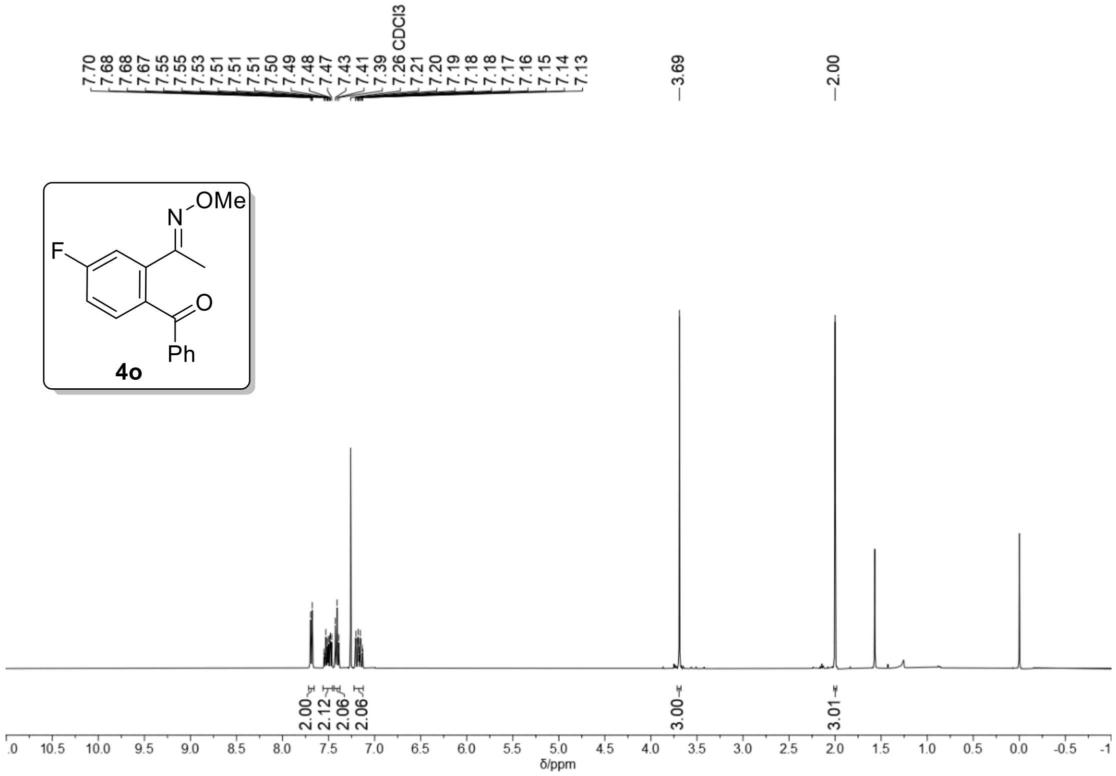

^{19}F NMR of compound **4o**

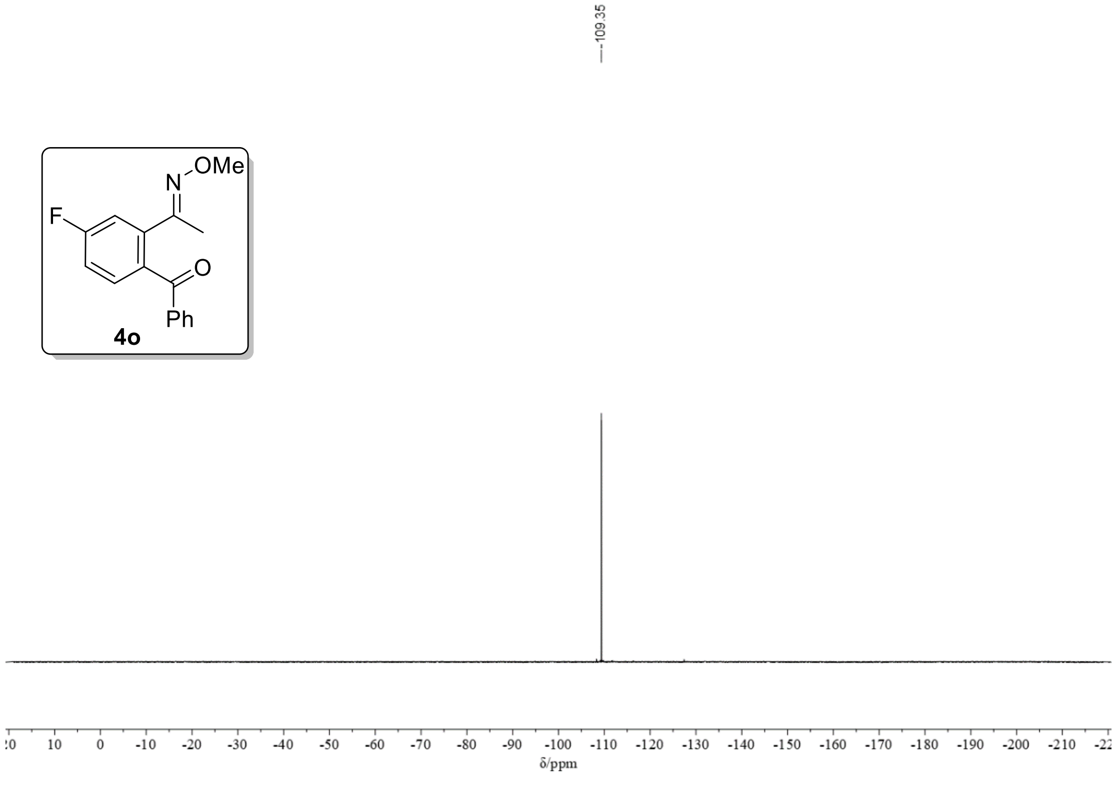

^{13}C NMR of compound **4o**

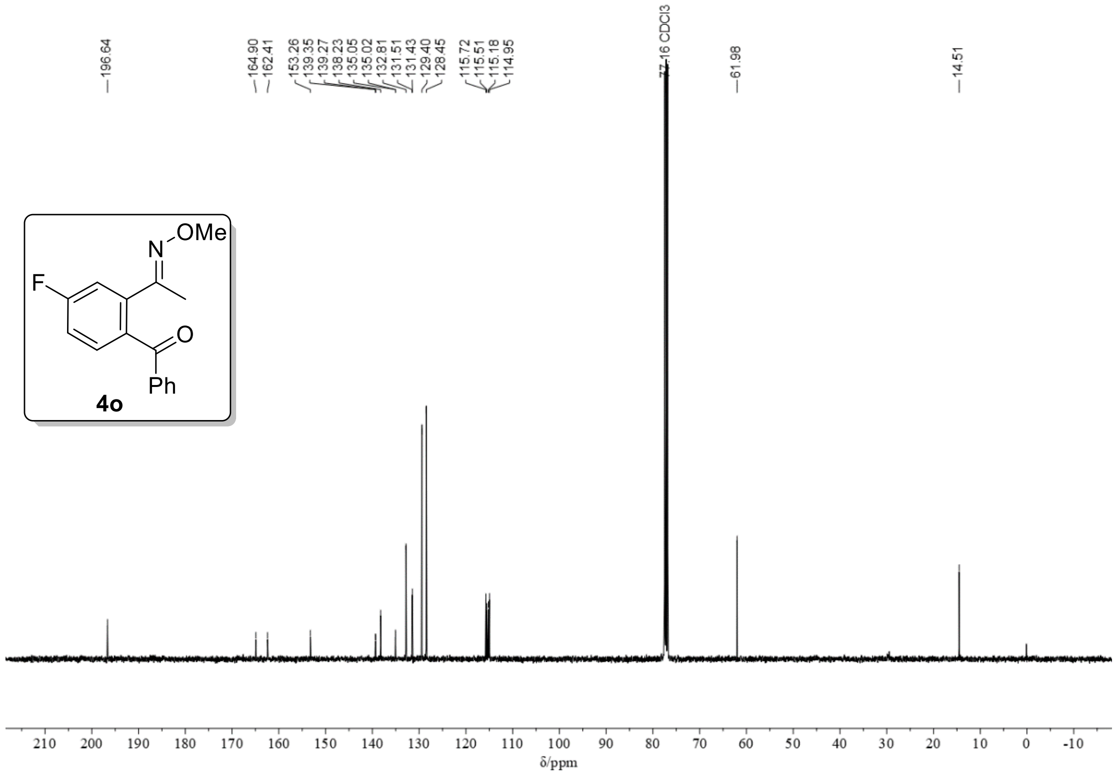

^{1}H NMR of compound **4o'**

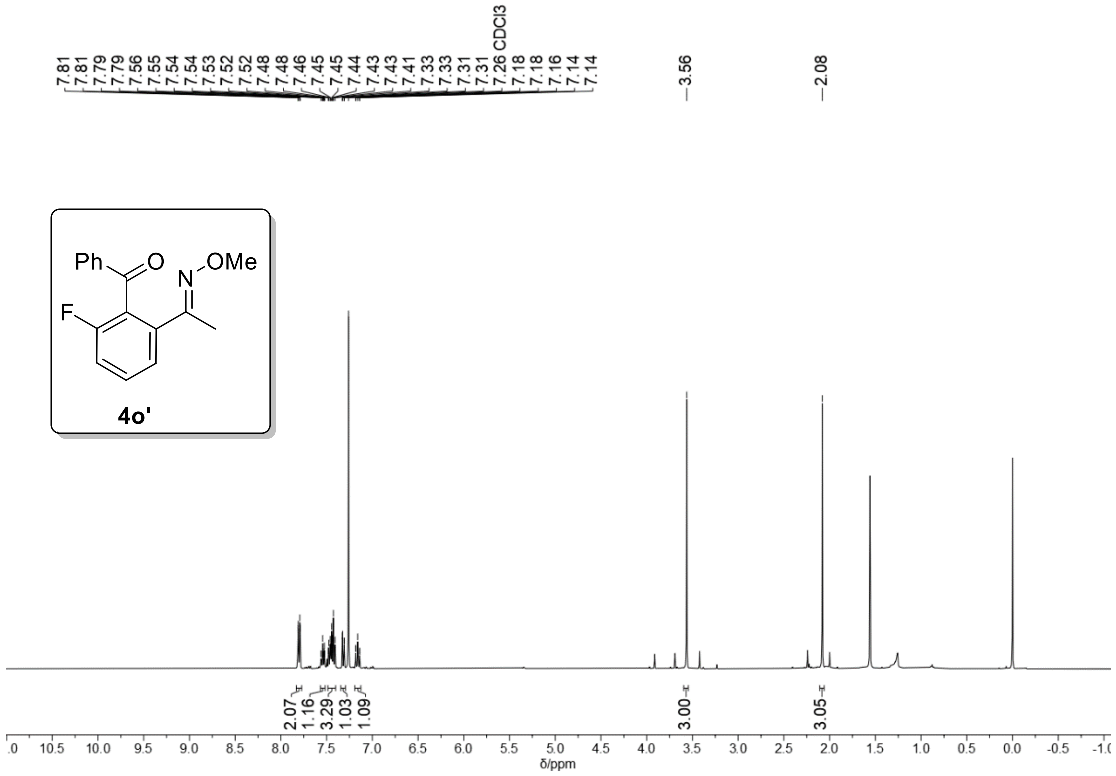

^{19}F NMR of compound **4o'**

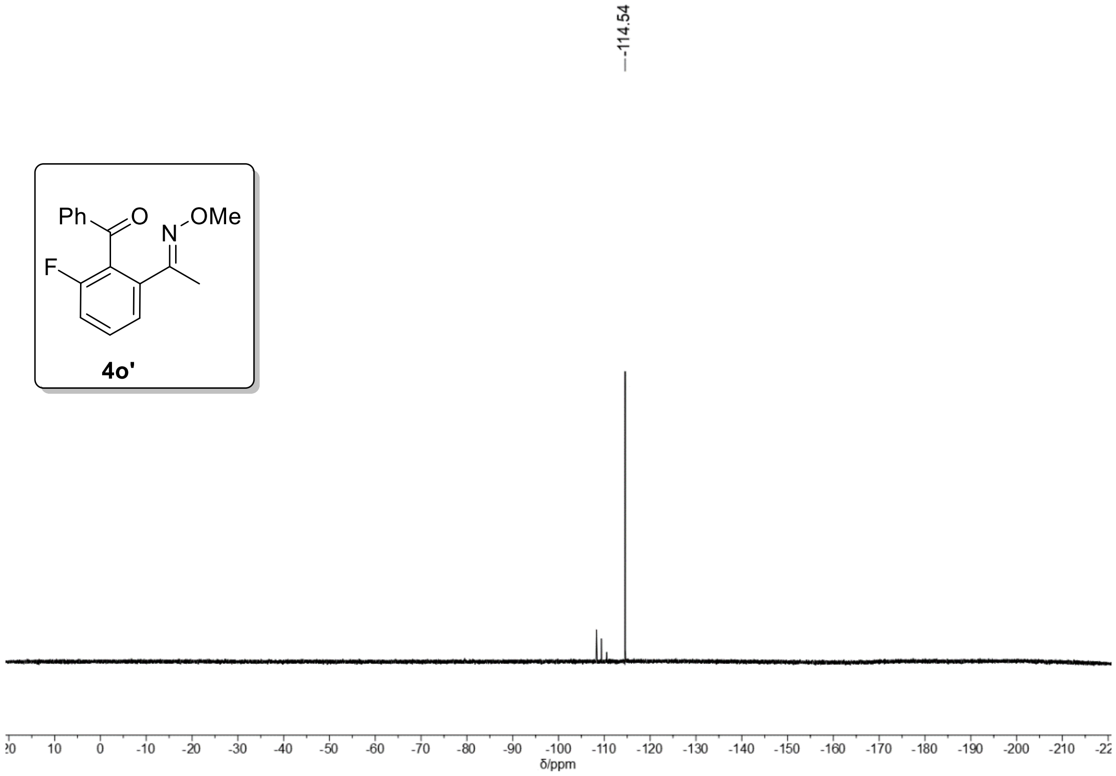

^{13}C NMR of compound **4o'**

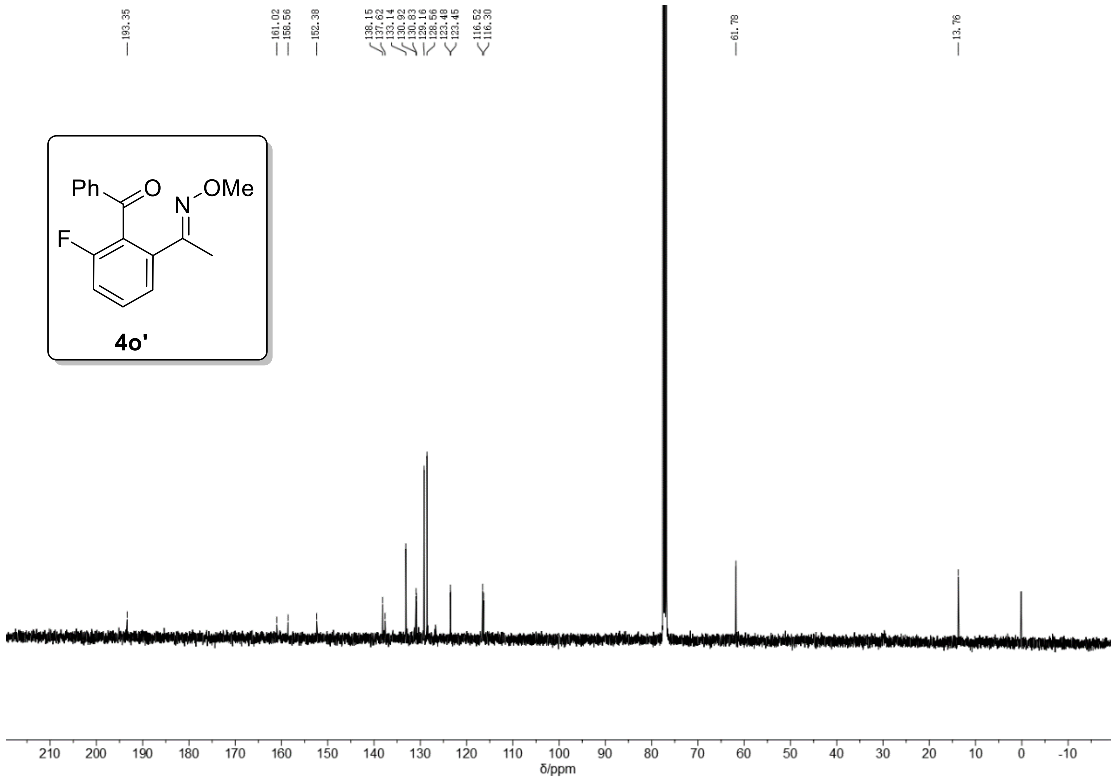

^{1}H NMR of compound **4p**

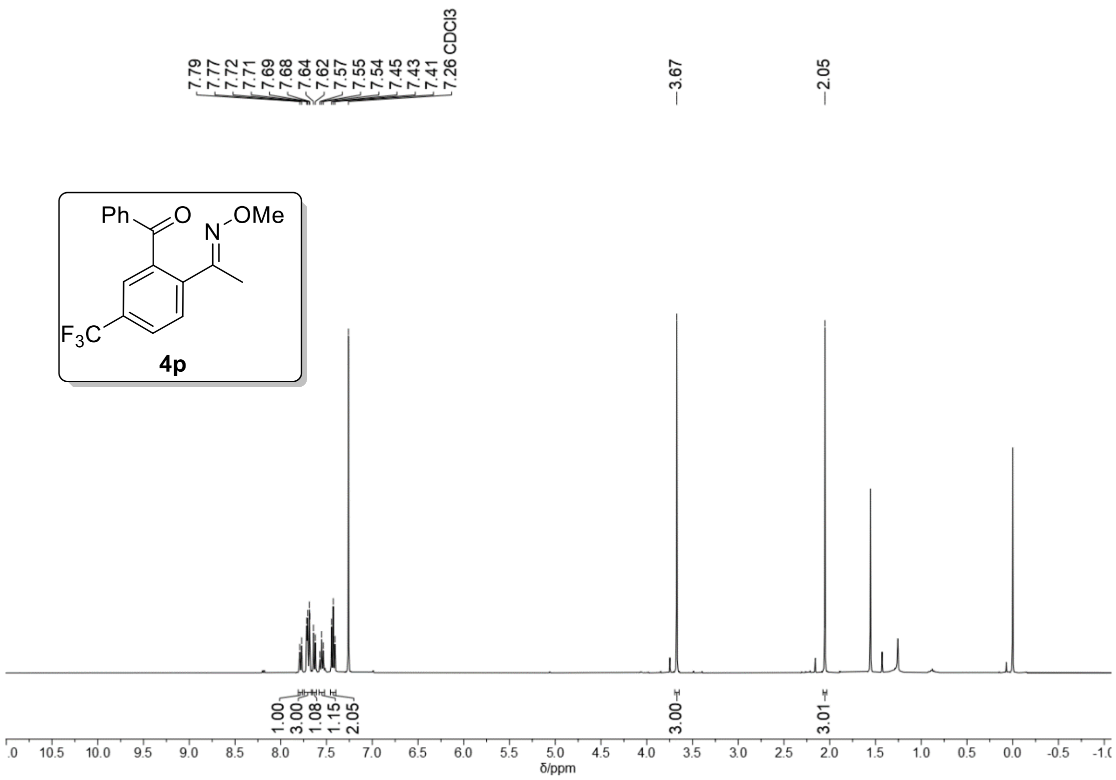

^{19}F NMR of compound **4p**

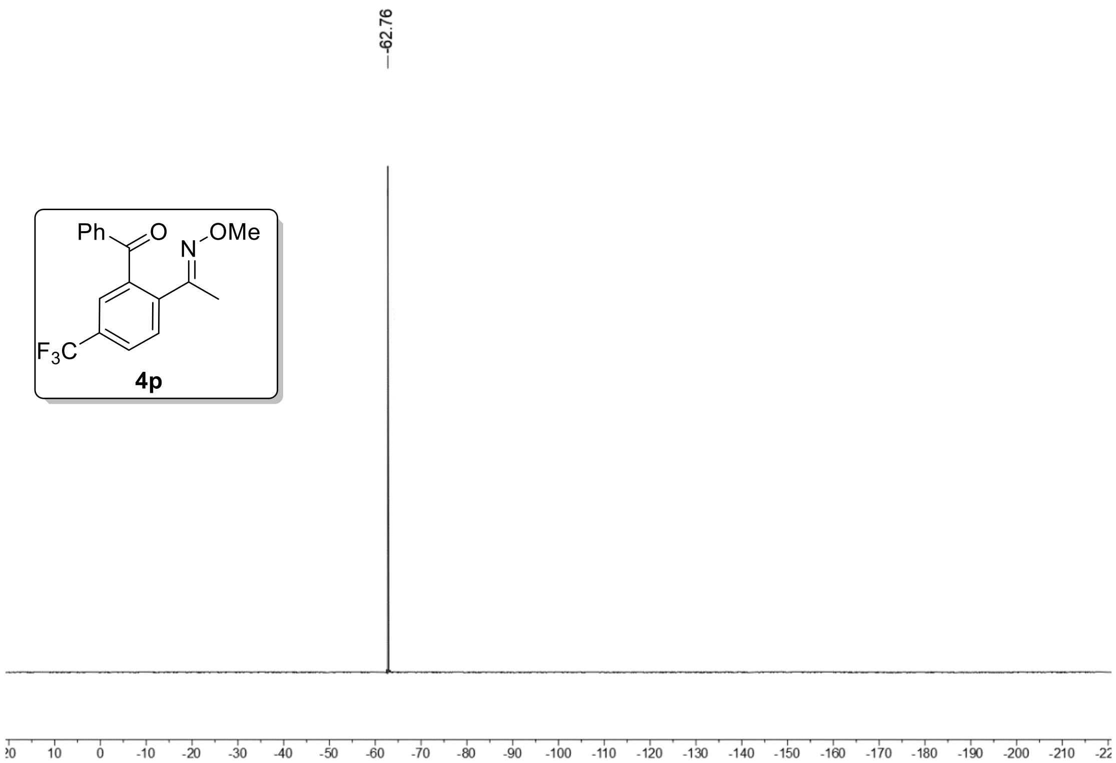

^{13}C NMR of compound **4p**

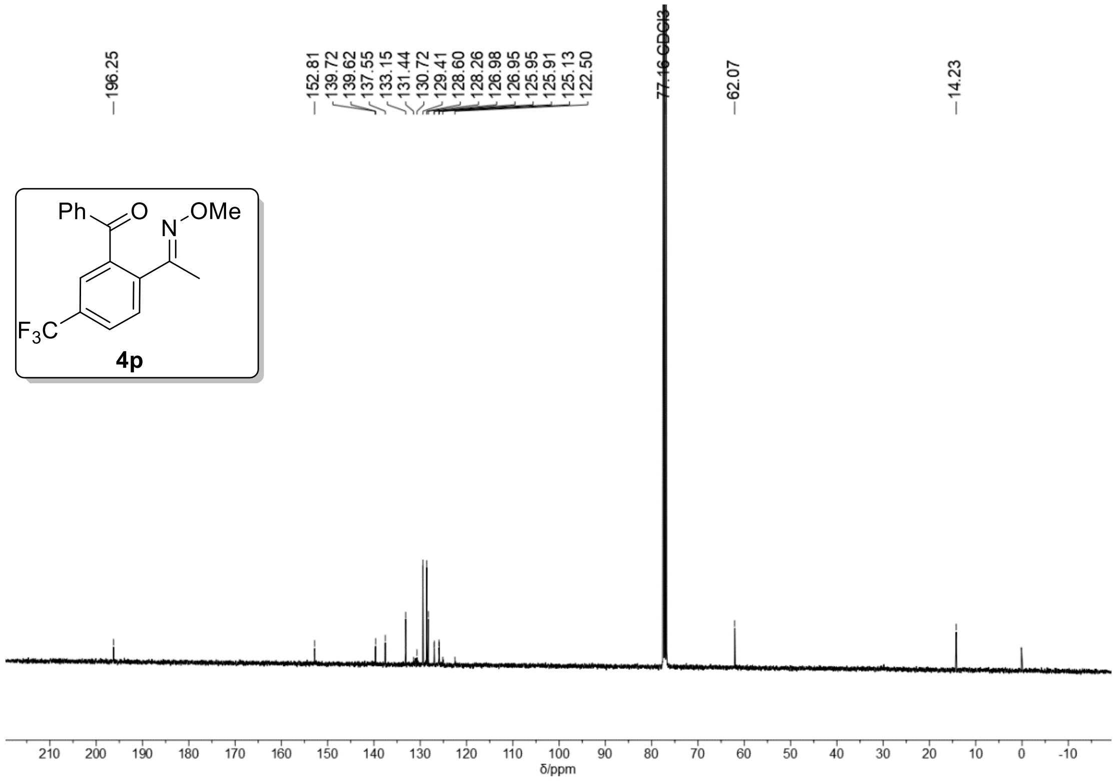

^{1}H NMR of compound **4q**

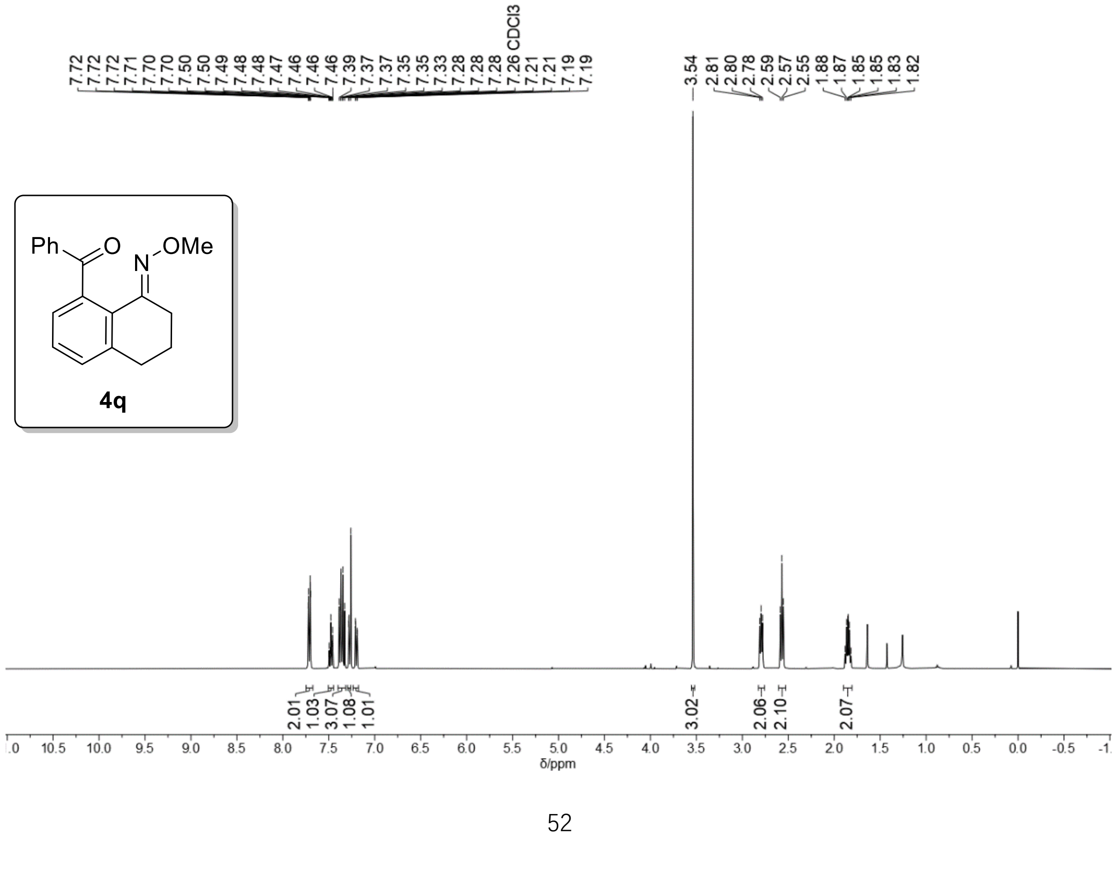

^{13}C NMR of compound **4q**

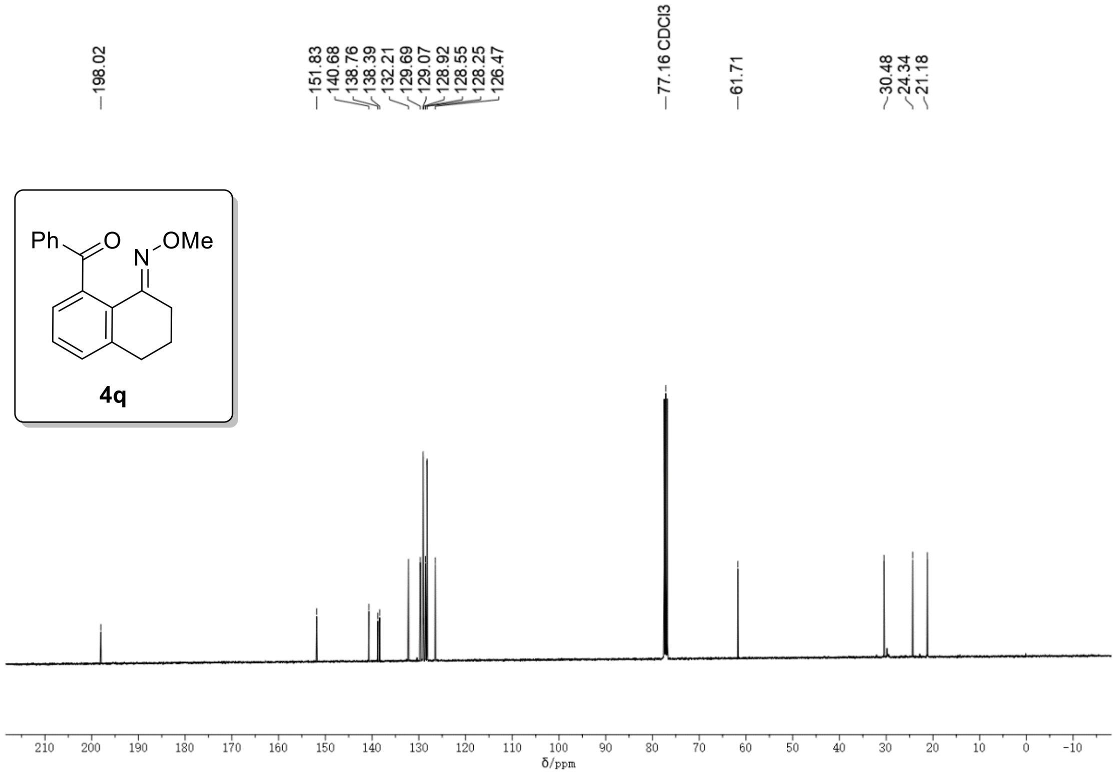

^{1}H NMR of compound **4r**

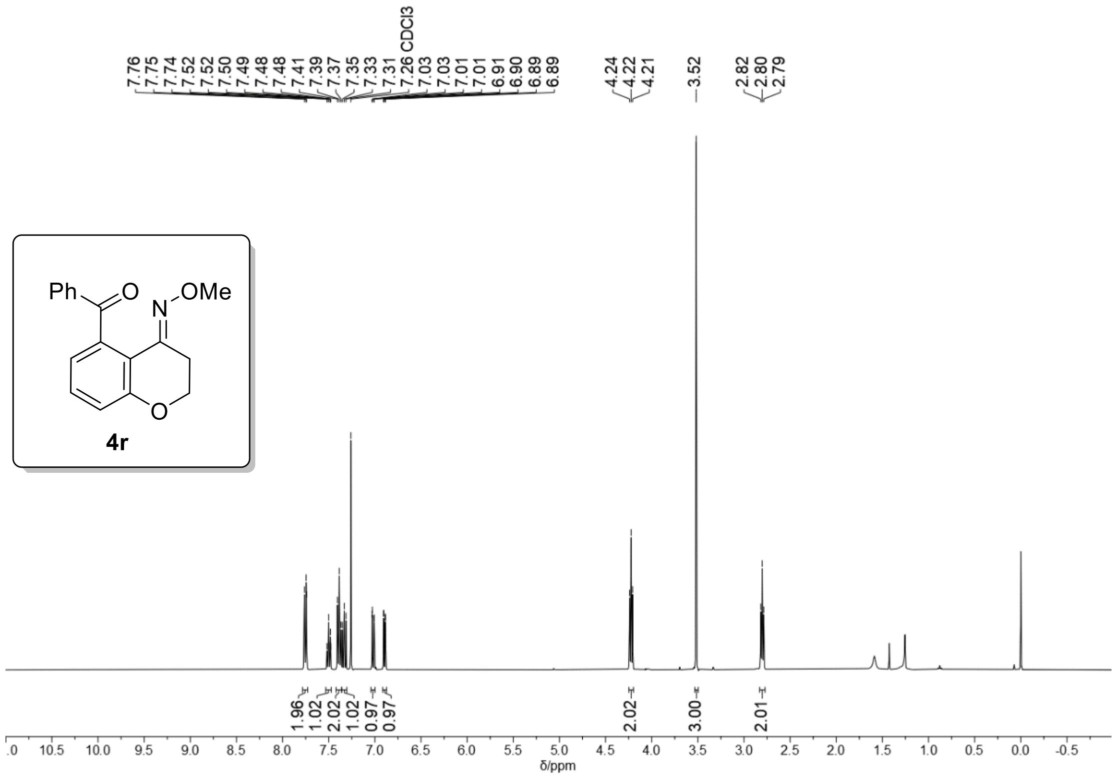

^{13}C NMR of compound **4r**

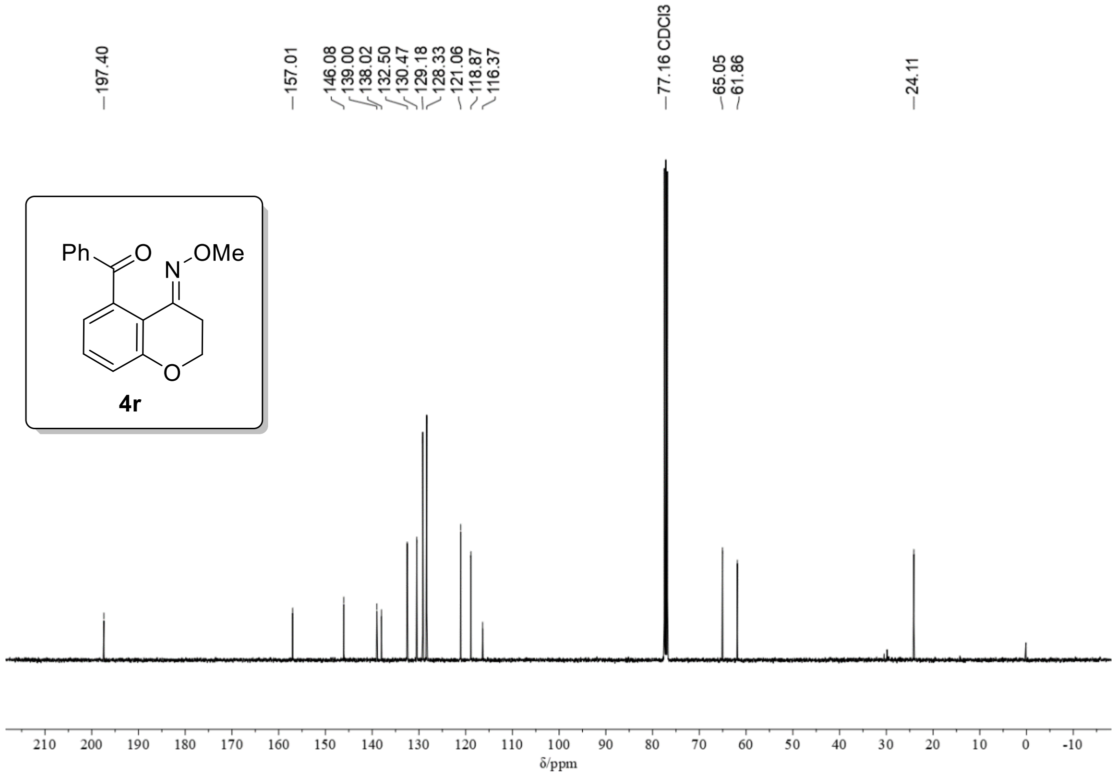

^{1}H NMR of compound **4s**

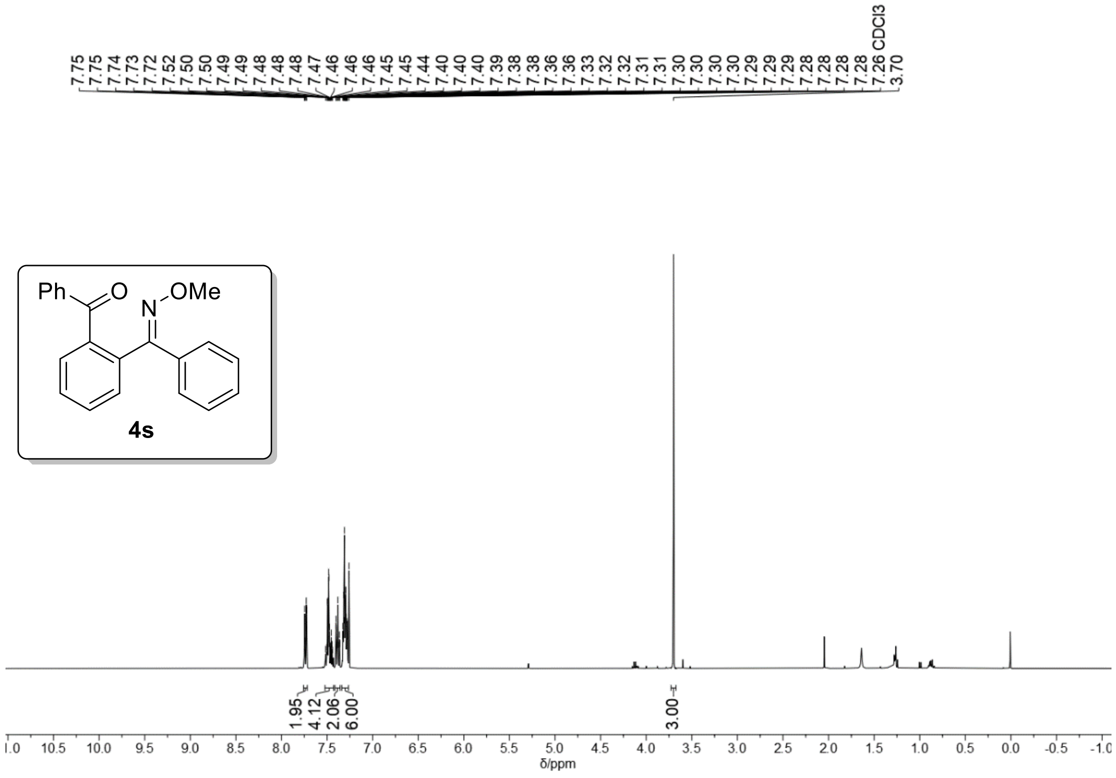

^{13}C NMR of compound **4s**

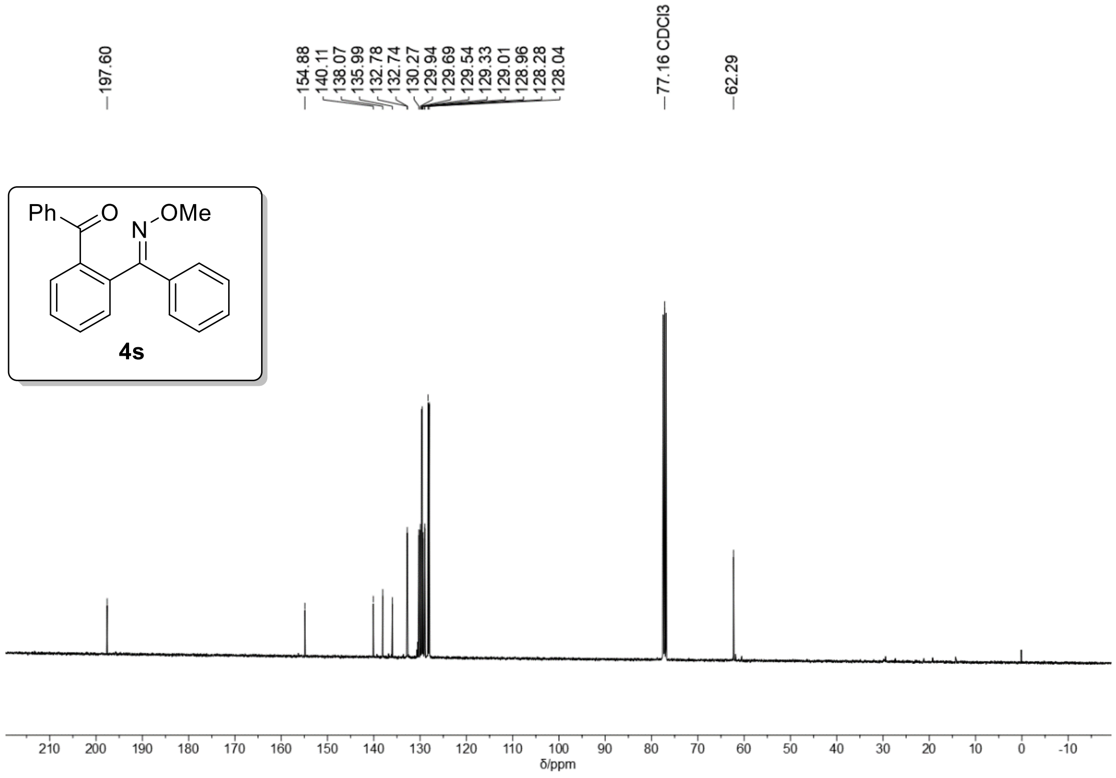

^{1}H NMR of compound **4t**

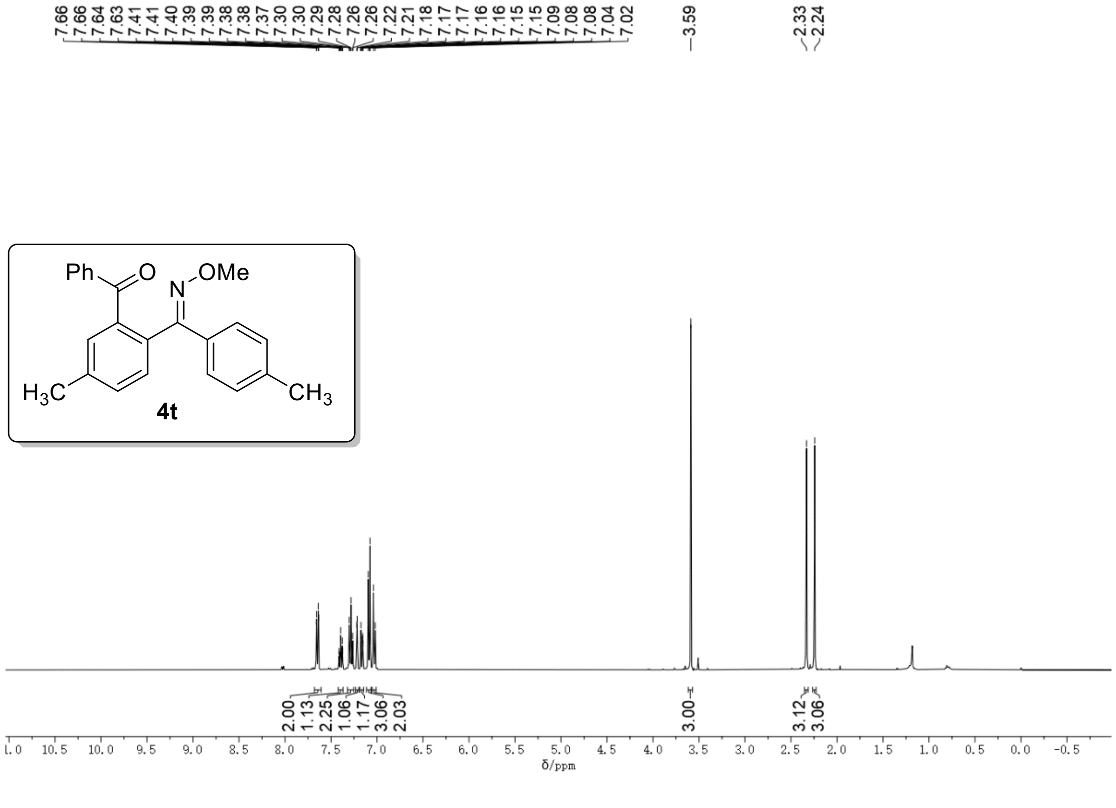

^{13}C NMR of compound **4t**

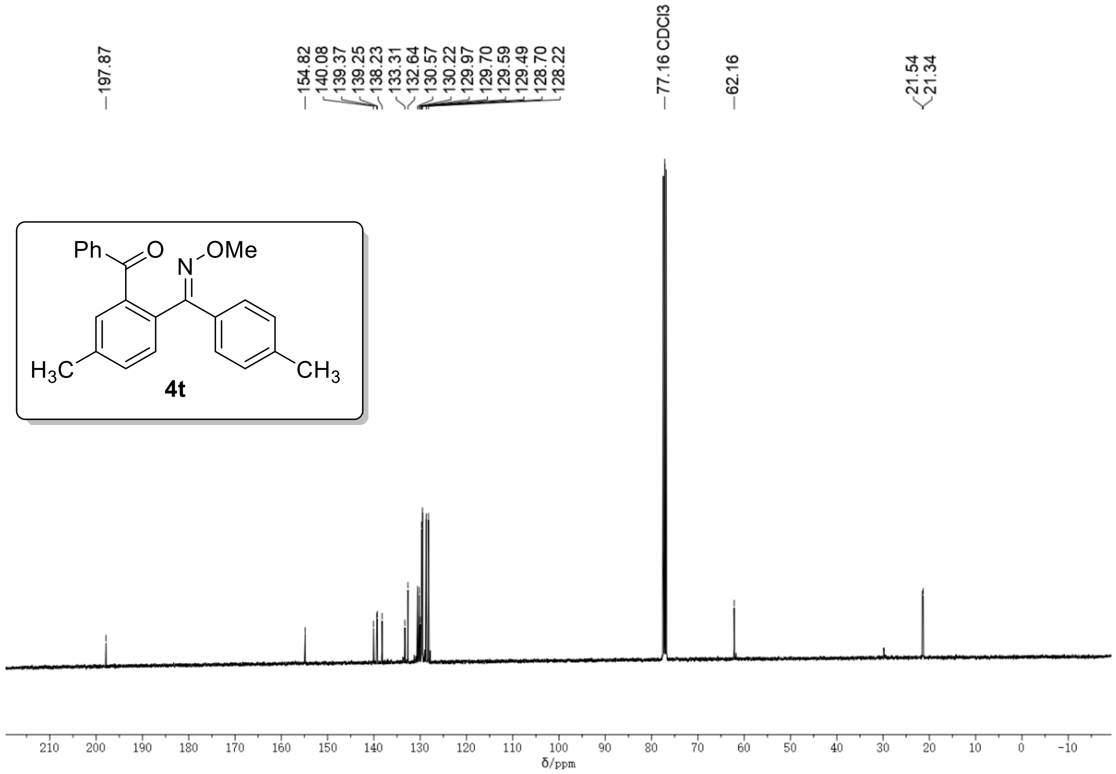

^{1}H NMR of compound **4u**

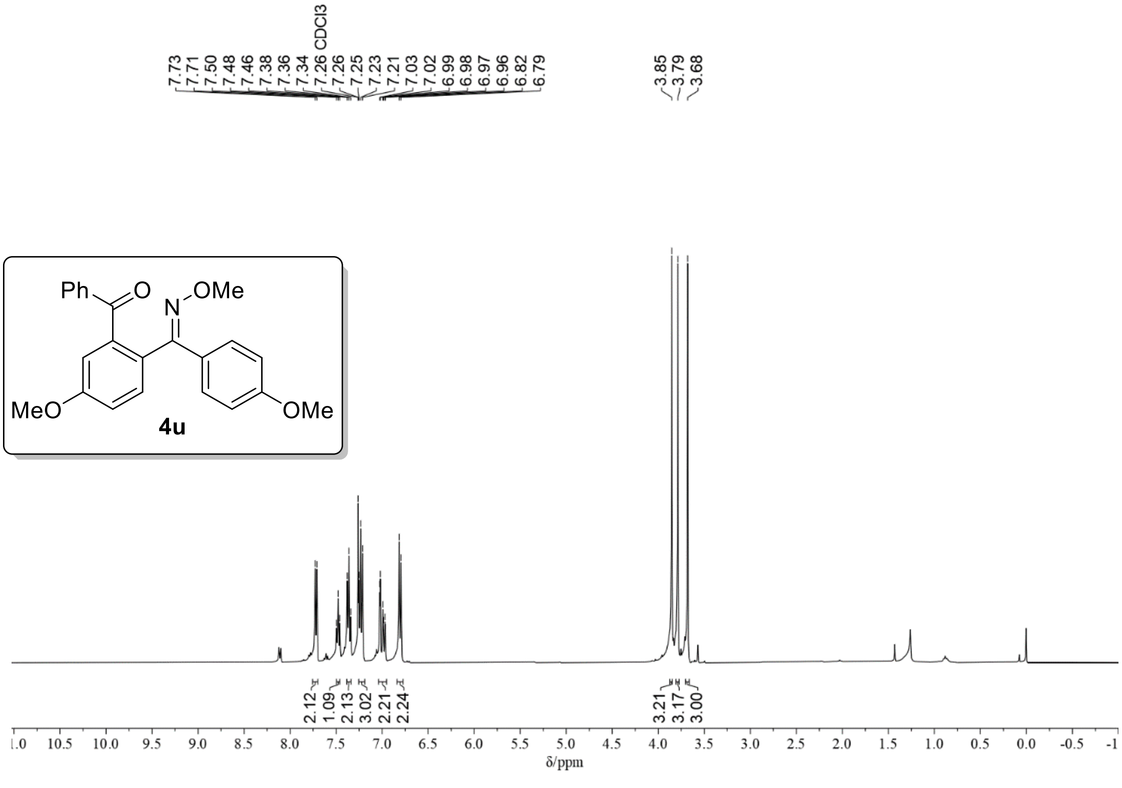

^{13}C NMR of compound **4u**

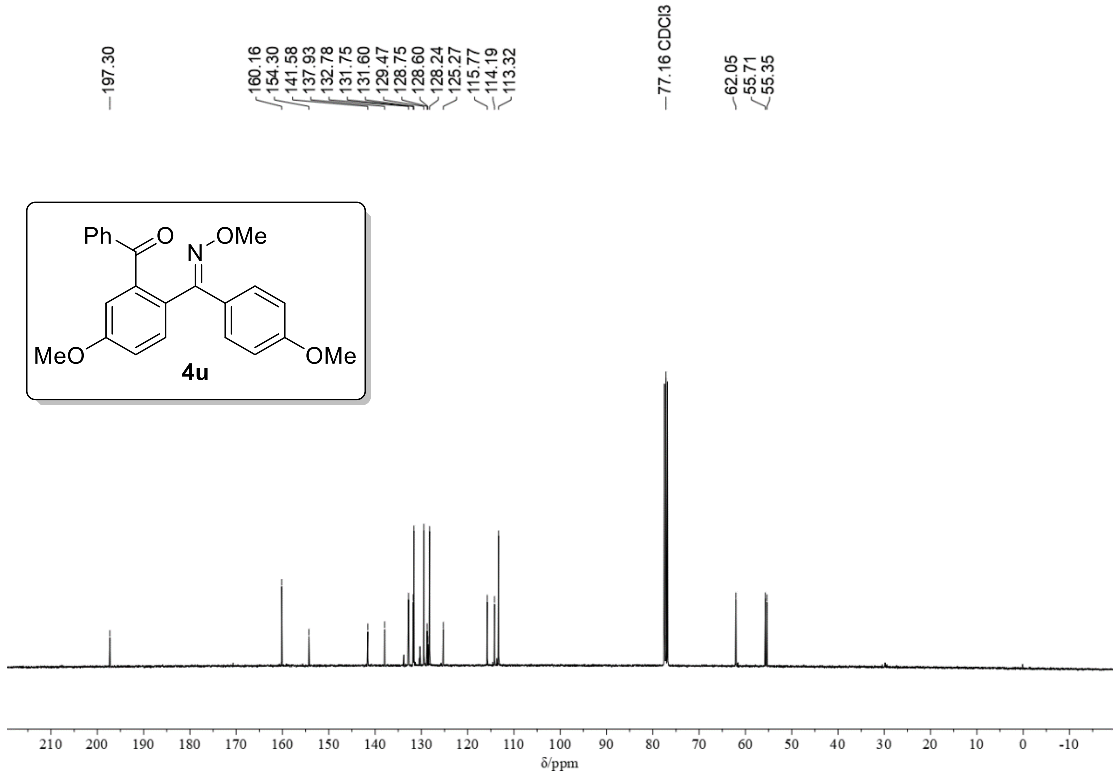

^{1}H NMR of compound **4v**

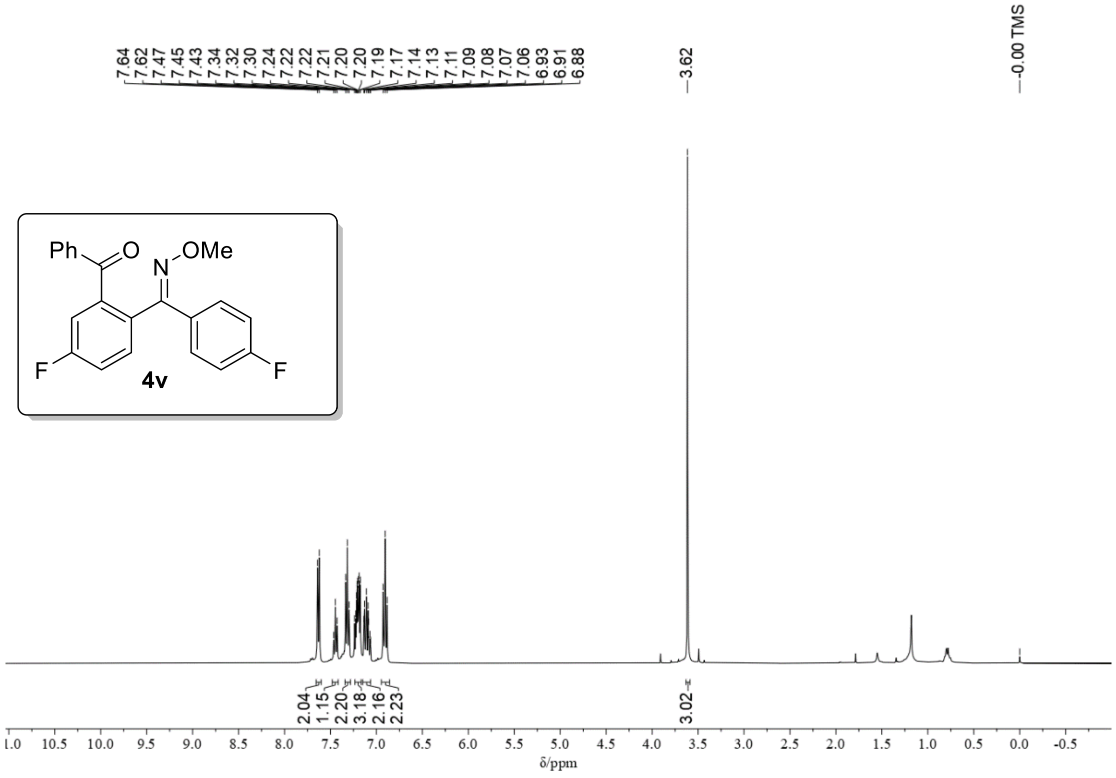

^{19}F NMR of compound **4v**

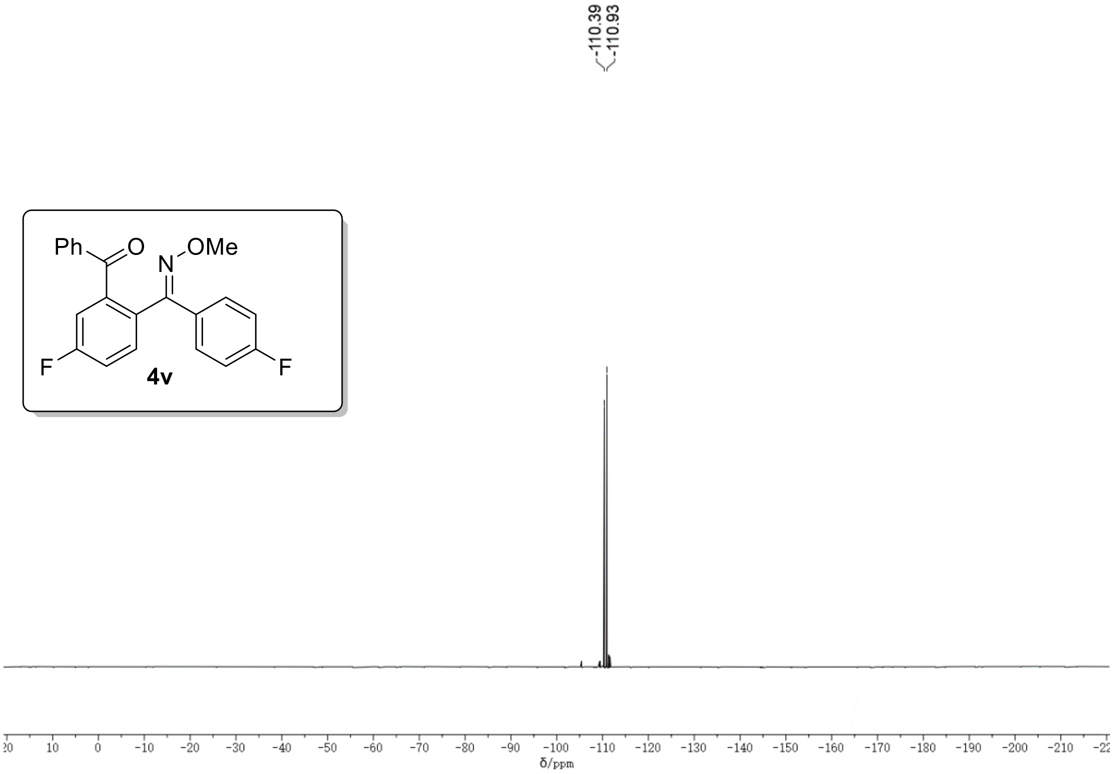

^{13}C NMR of compound **4v**

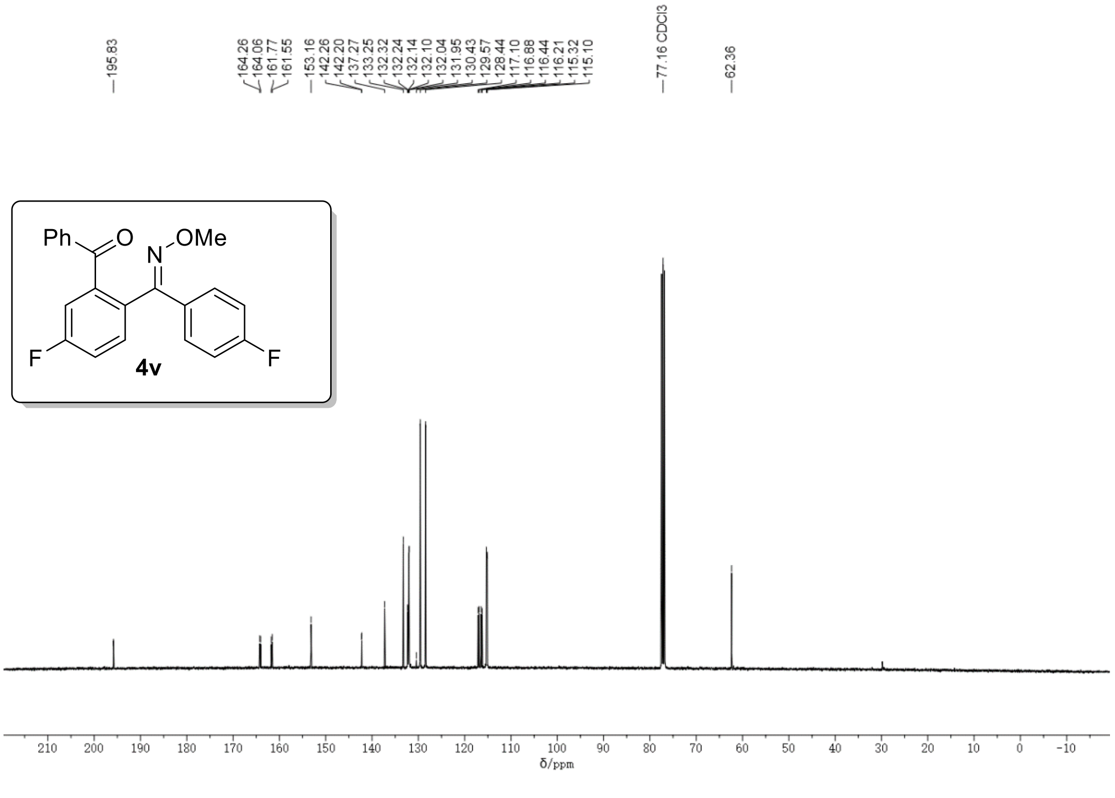

^{1}H NMR of compound (**4w+4w'**)

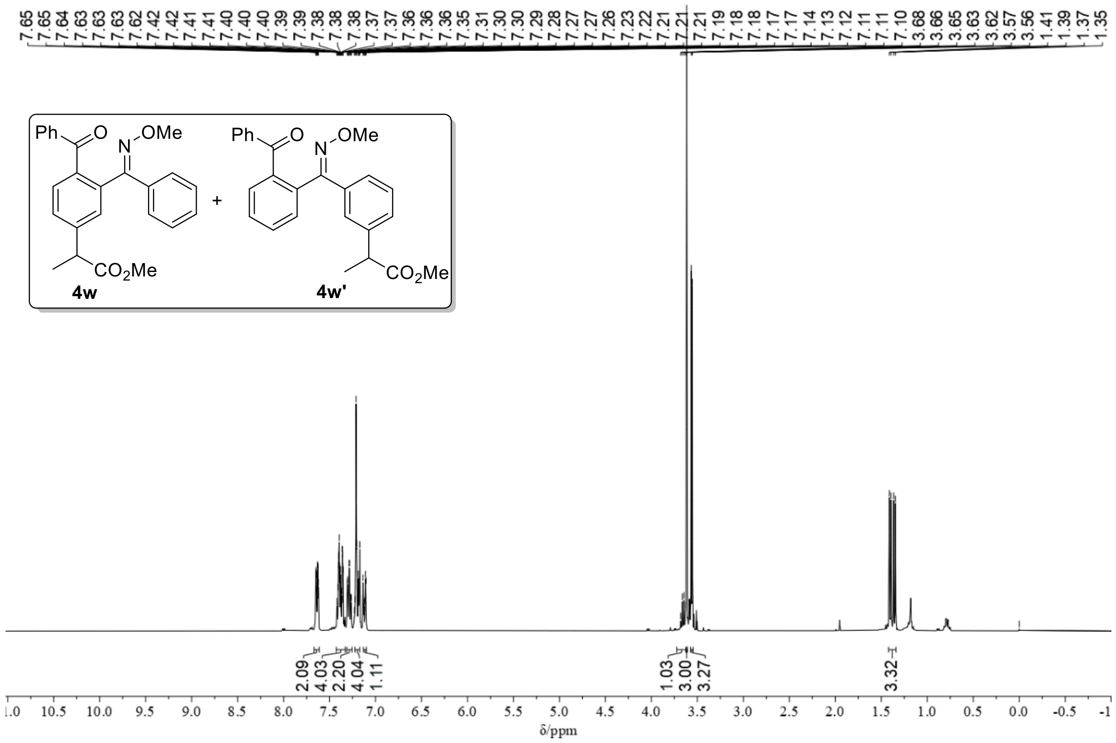

^{13}C NMR of compound (**4w+4w'**)

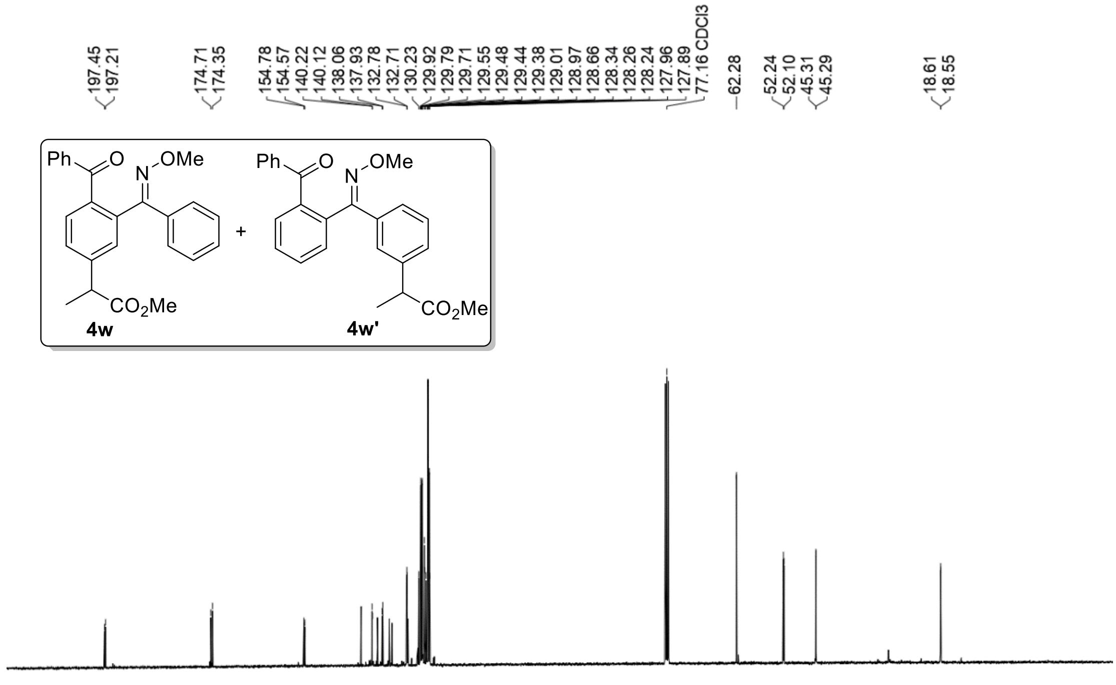

^{1}H NMR of compound **5**

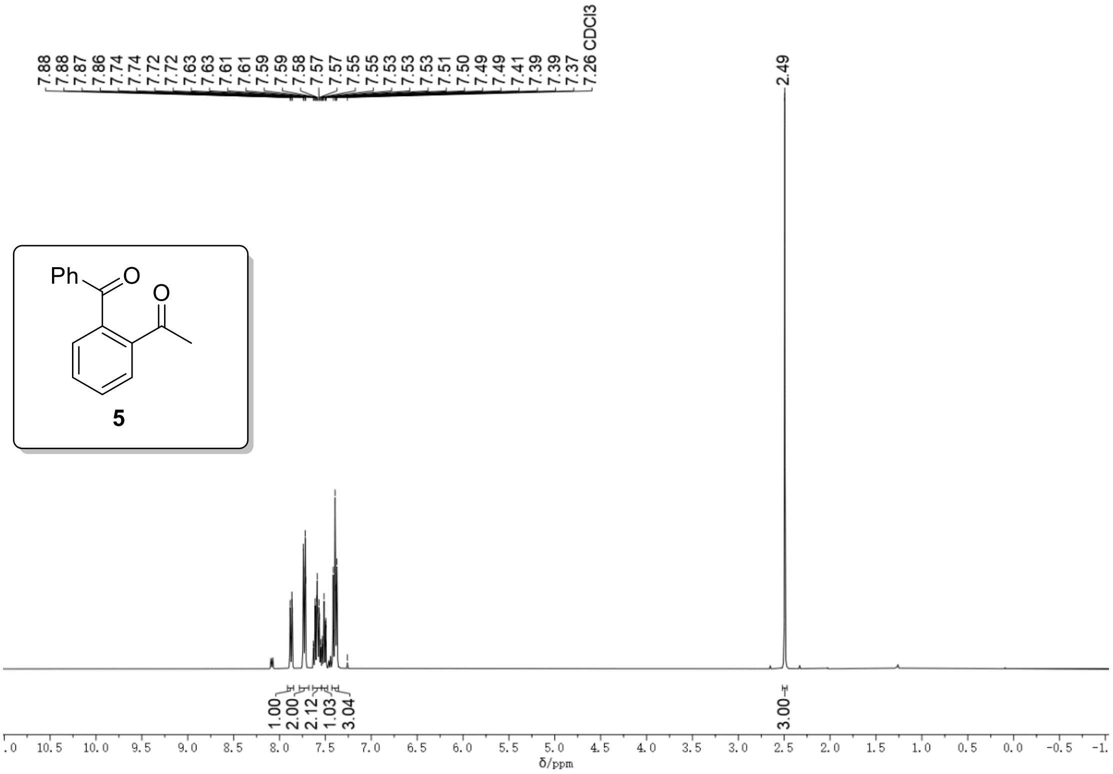

^{13}C NMR of compound **5**

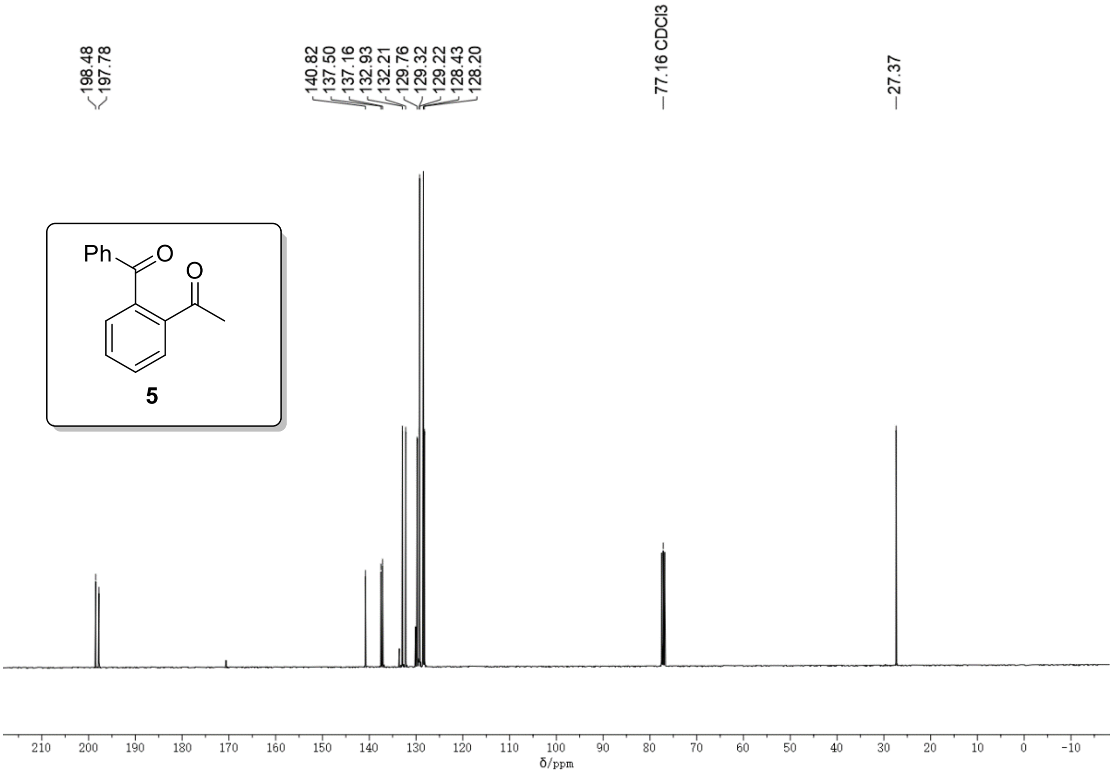

^{1}H NMR of compound **6**

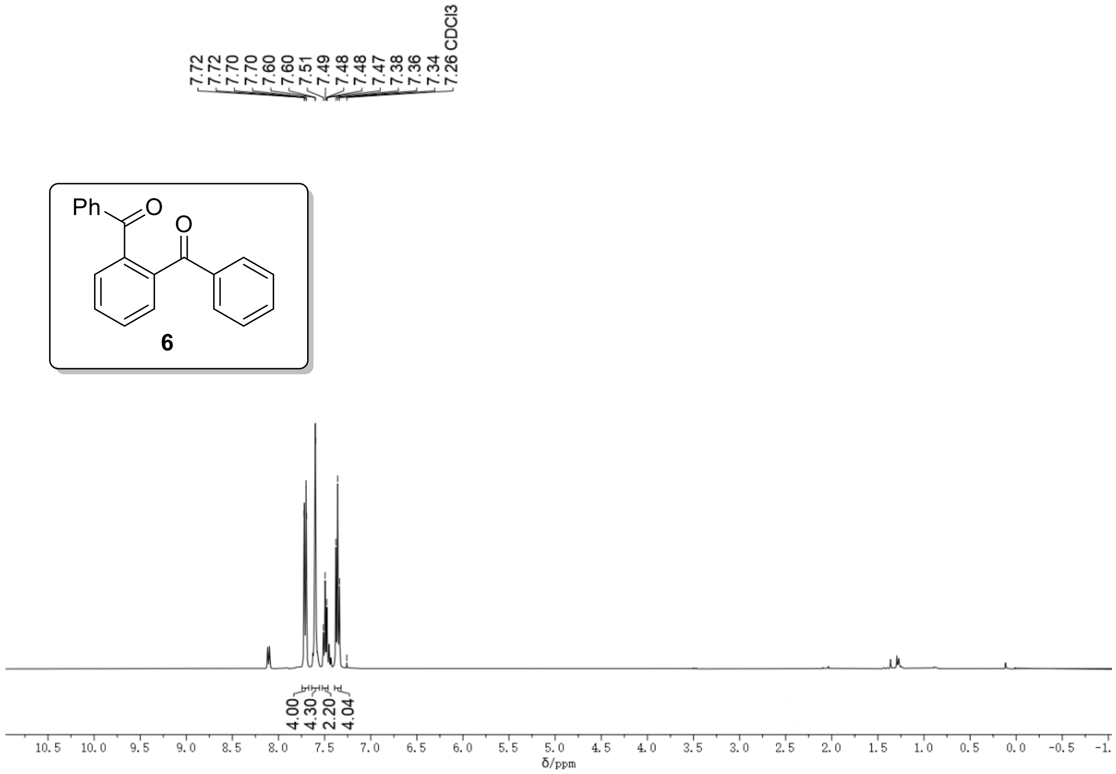

^{13}C NMR of compound **6**

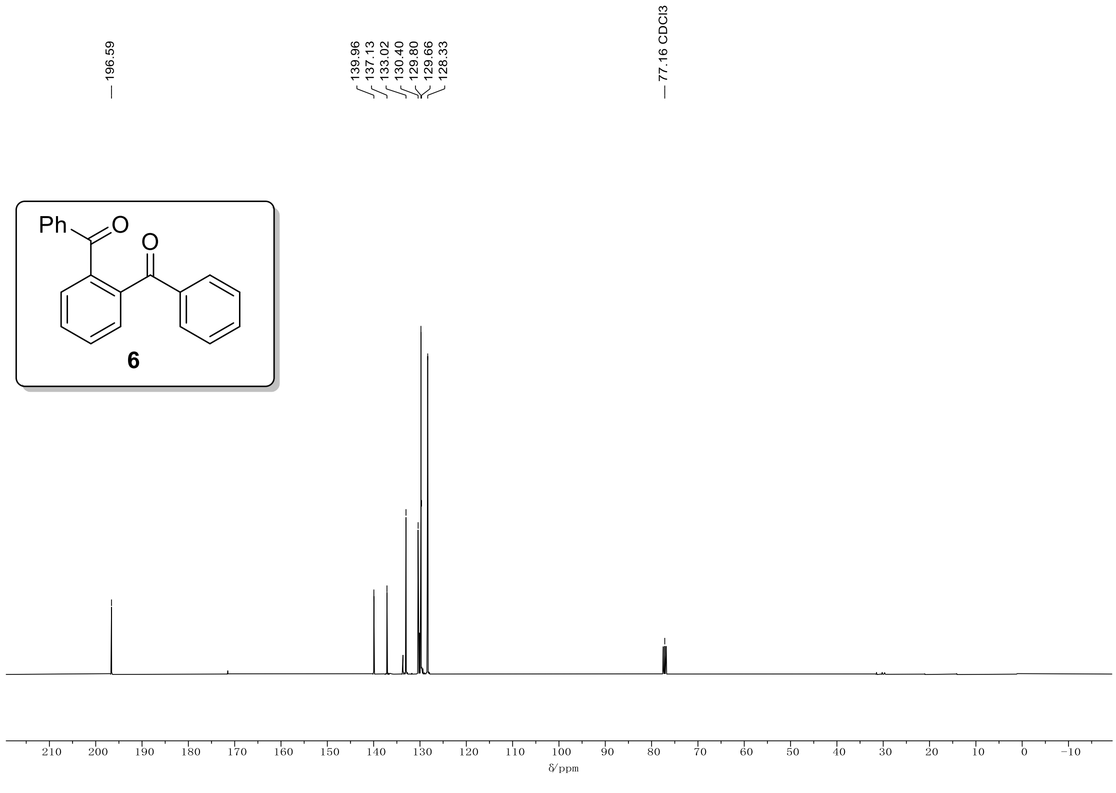

^{1}H NMR of compound **7**

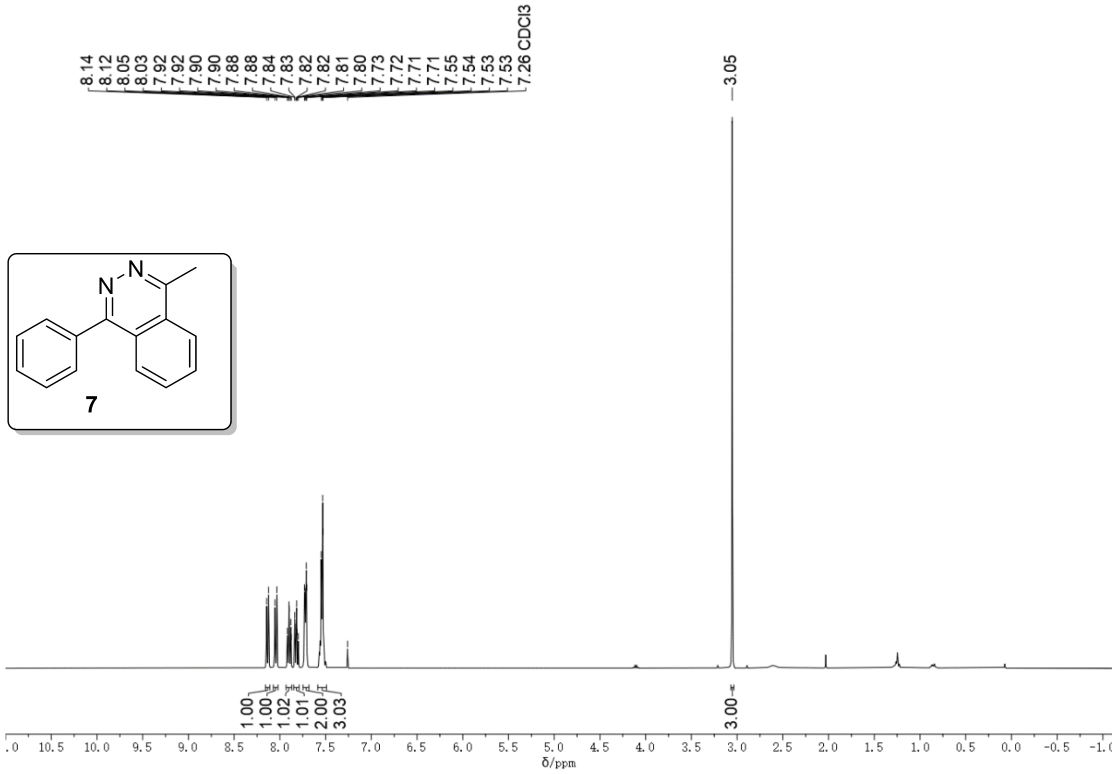

^{13}C NMR of compound **7**

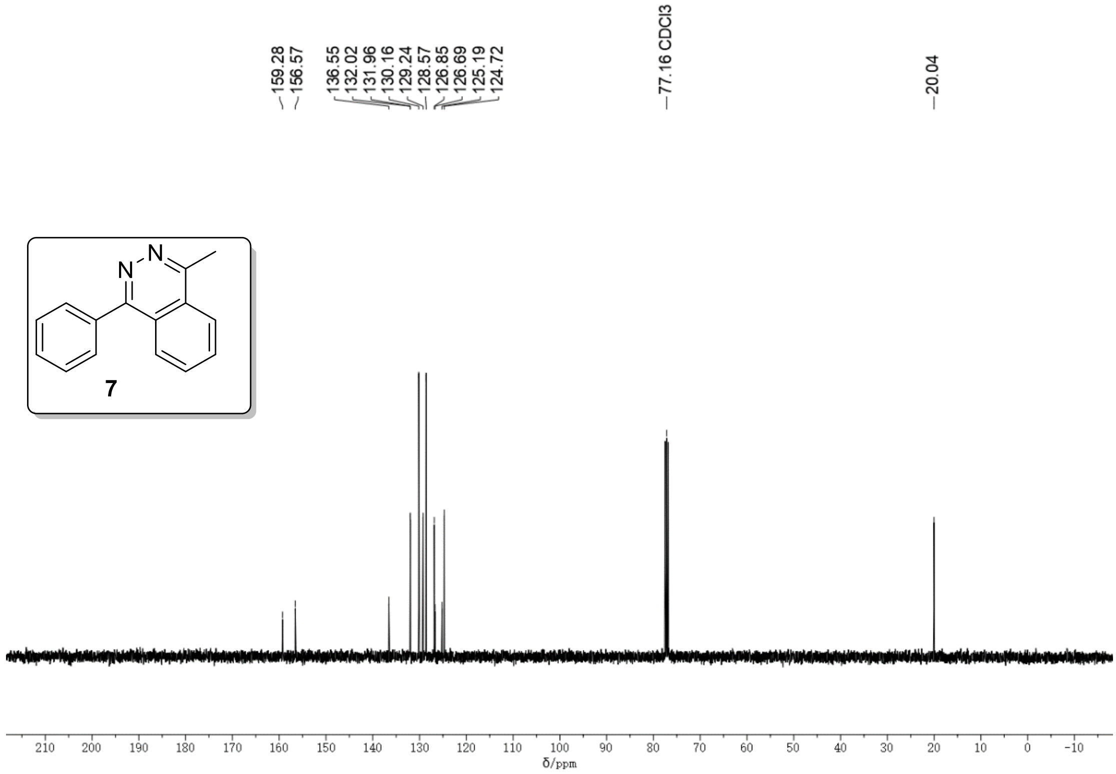

^{1}H NMR of compound **8**

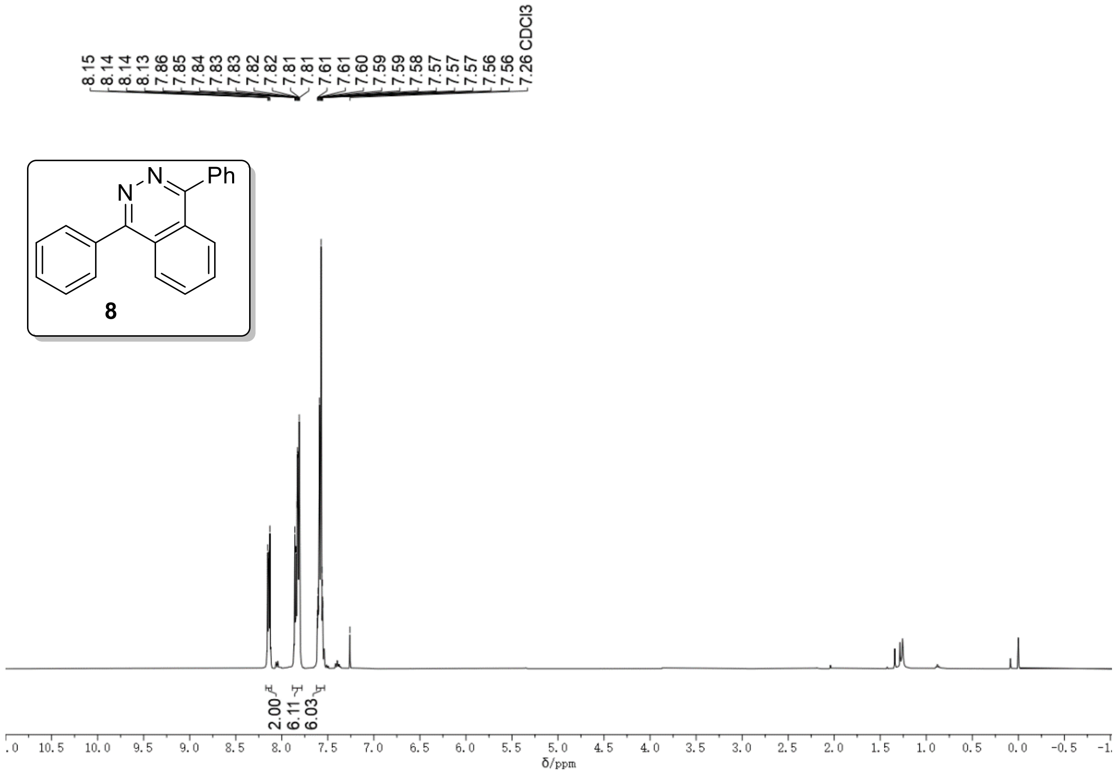

^{13}C NMR of compound **8**

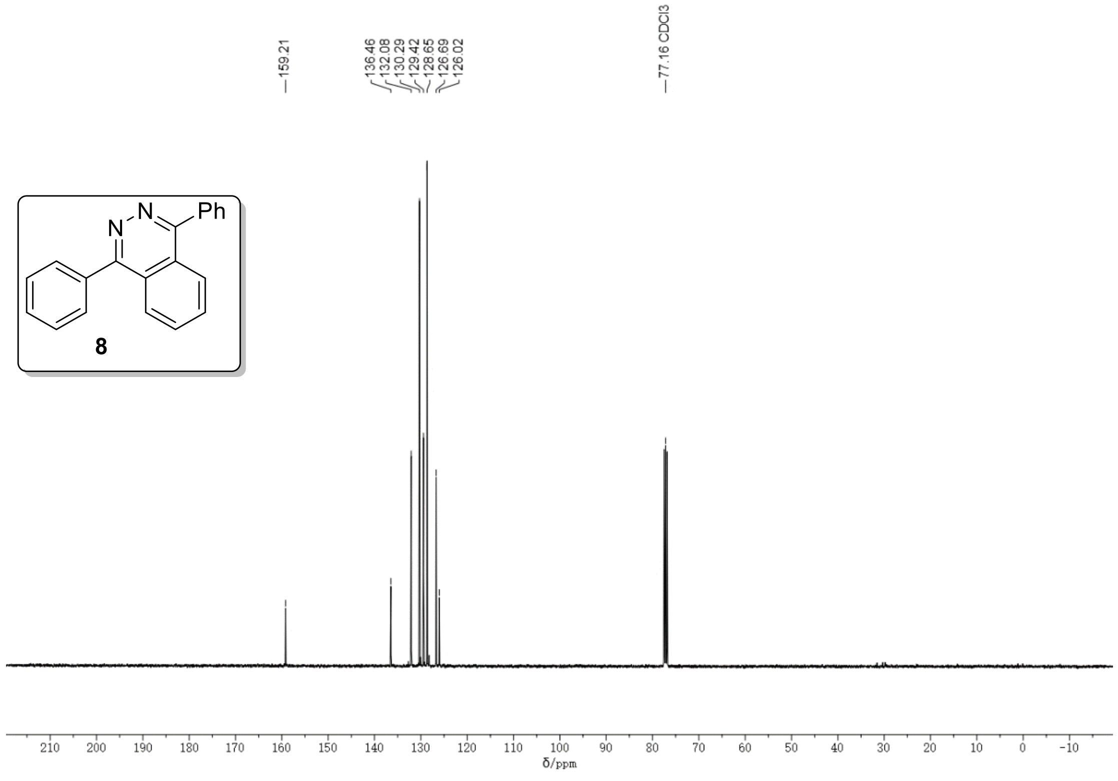

^{1}H NMR of compound **9**

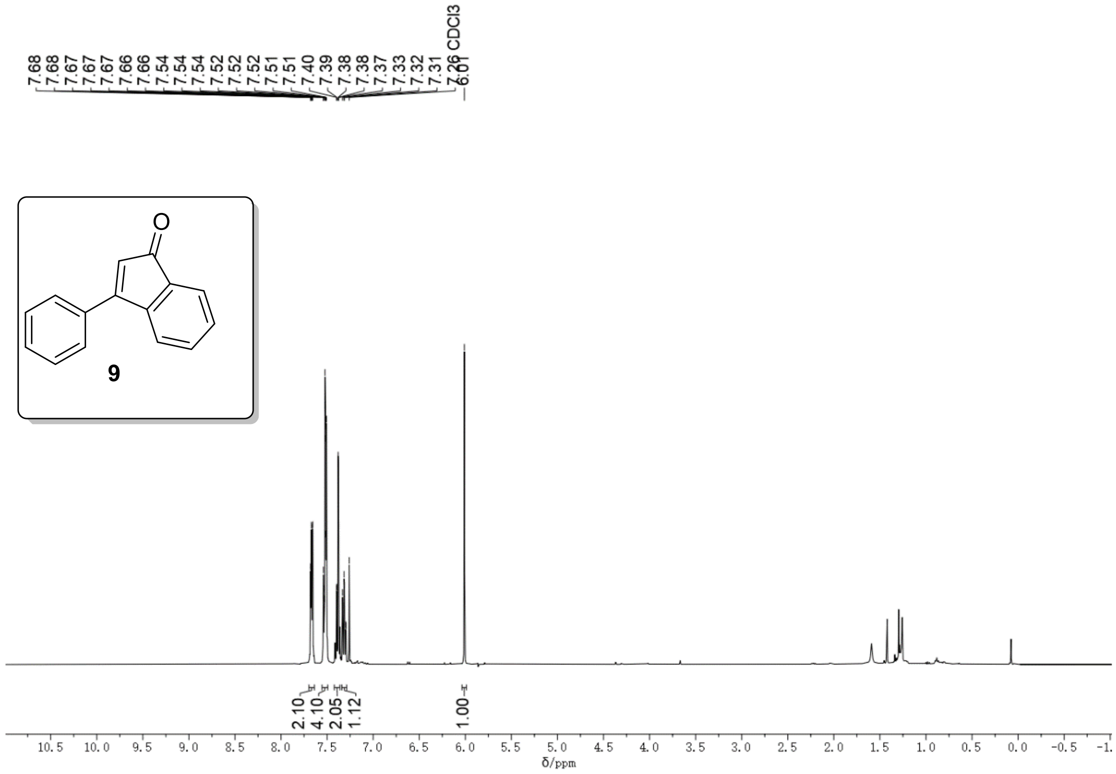

^{13}C NMR of compound **9**

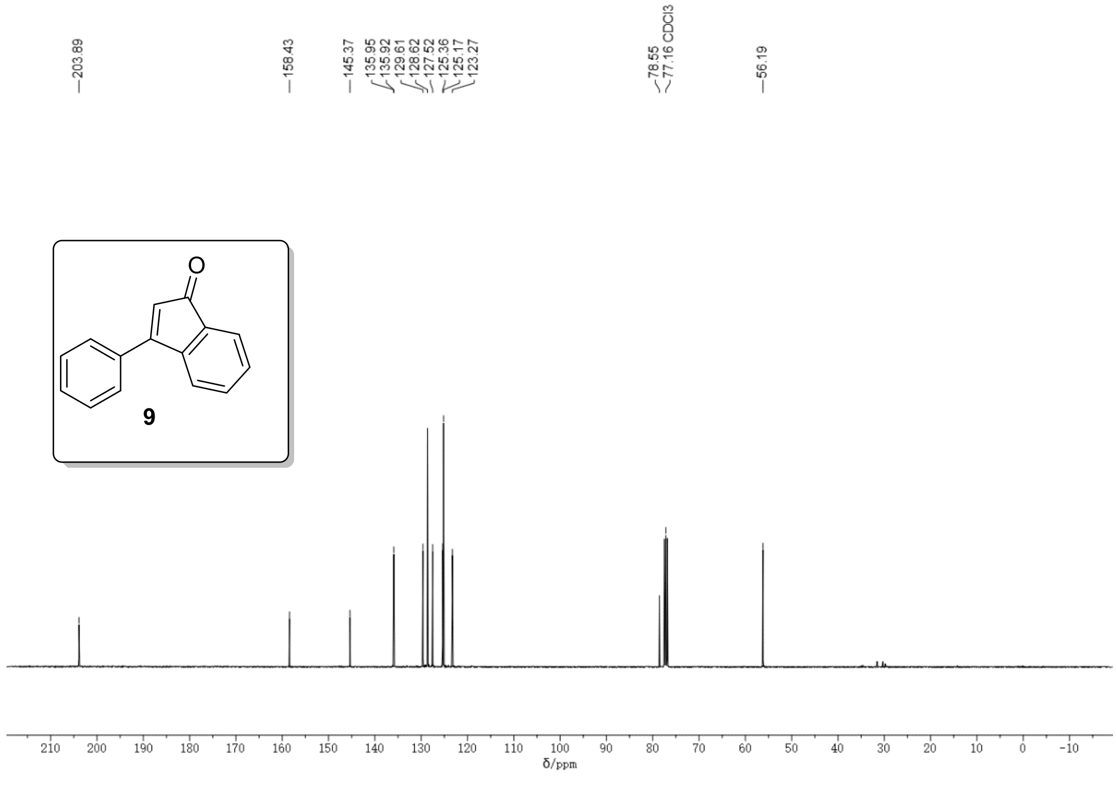

^{1}H NMR of compound **10**

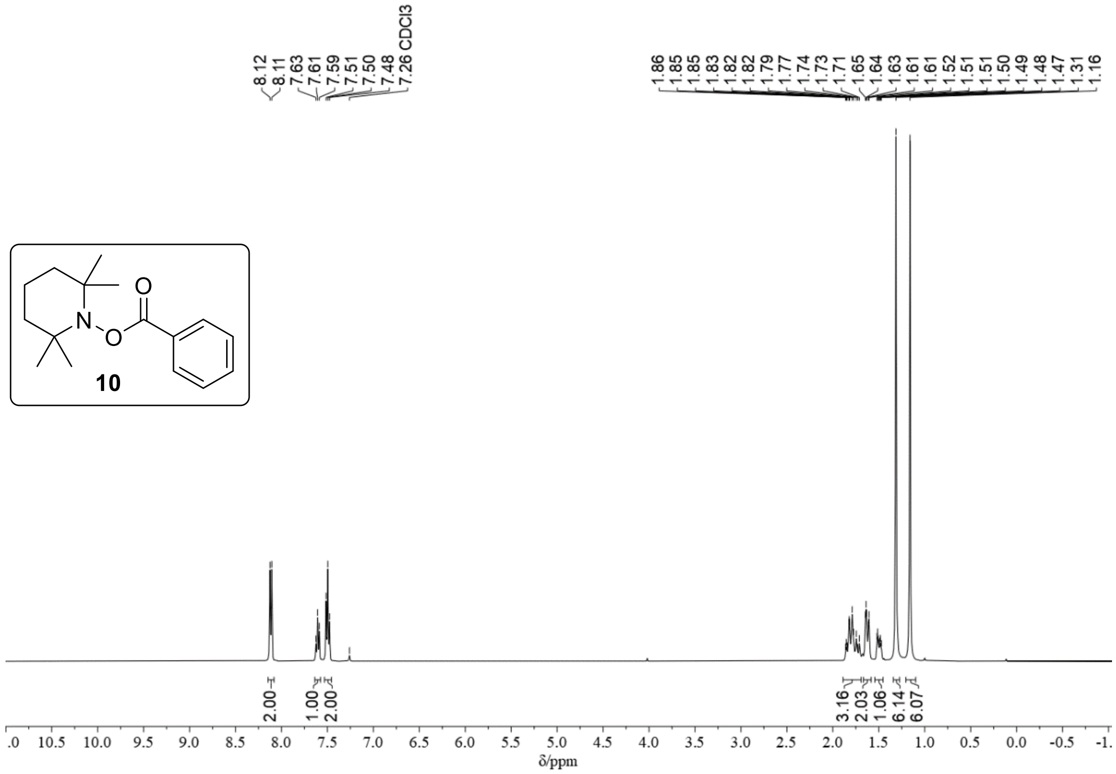

^{13}C NMR of compound **10**

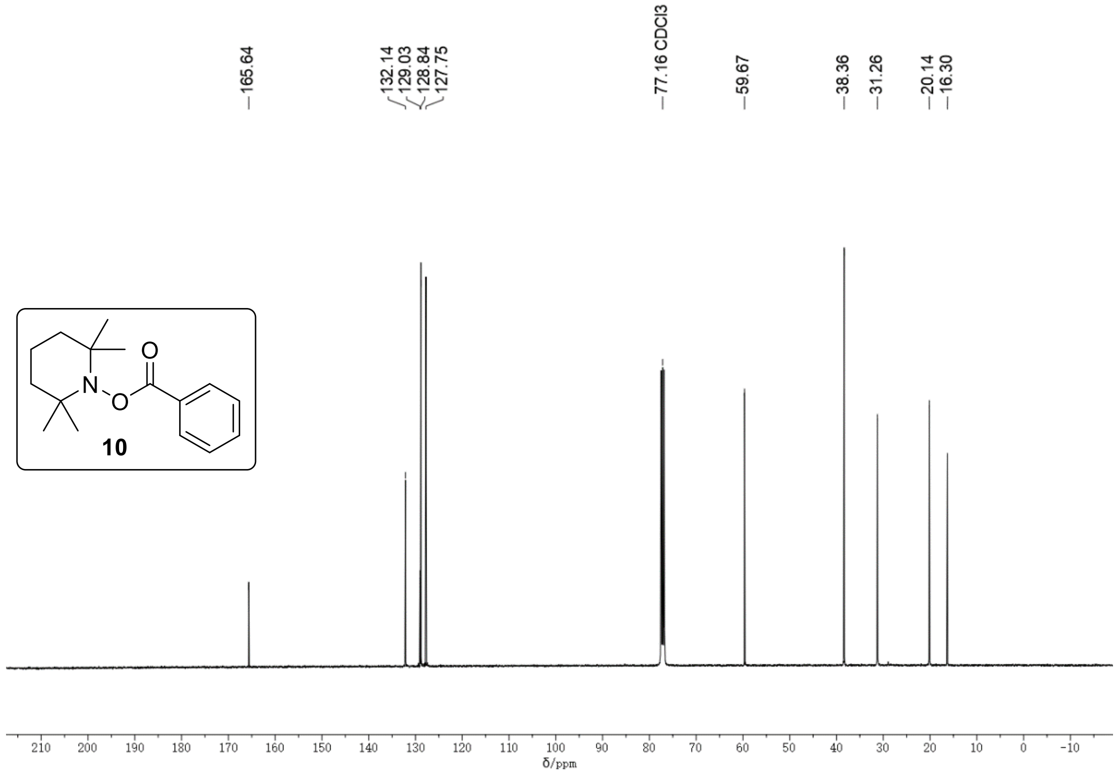

Supplement: Supplementary file 1 [file molecules-27-01980-s001.zip › molecules-1625747-supplementary.pdf]
